# A Review of Zoonotic Disease Threats to Pet Owners: A Compendium of Measures to Prevent Zoonotic Diseases Associated with Non-Traditional Pets Such as Rodents and Other Small Mammals, Reptiles, Amphibians, Backyard Poultry, and Other Selected Animals

**DOI:** 10.1089/vbz.2022.0022

**Published:** 2022-06-17

**Authors:** Kate Varela, Jennifer A. Brown, Beth Lipton, John Dunn, Danielle Stanek, Casey Barton Behravesh, Helena Chapman, Terry H. Conger, Tiffany Vanover, Thomas Edling, Stacy Holzbauer, Angela M. Lennox, Scott Lindquist, Suzan Loerzel, Shelley Mehlenbacher, Mark Mitchell, Michael Murphy, Christopher W. Olsen, Cody M. Yager

**Affiliations:** ^1^ One Health Office, Centers for Disease Control and Prevention, Atlanta, Georgia, USA.; ^2^ National Association of State Public Health Veterinarians.; ^3^ Indiana Department of Health.; ^4^ Seattle & King County Public Health.; ^5^ Tennessee Department of Health.; ^6^ Florida Department of Health.; ^7^ Division of Infectious Diseases and Global Medicine, University of Florida College of Medicine.; ^8^ American Association for the Advancement of Science at NASA Applied Sciences.; ^9^ U.S. Department of Agriculture Animal (USDA) and Plant Health Inspection Service (APHIS) Veterinary Services.; ^10^ International Poultry Retail Consultant.; ^11^ American Humane.; ^12^ Pet Advocacy Network Consultant.; ^13^ Minnesota Department of Health.; ^14^ CDC Preparedness and Response Career Epidemiology Field Officer Program.; ^15^ Avian and Exotic Animal Clinic (Indianapolis).; ^16^ Washington State Department of Health.; ^17^ USDA APHIS Animal Care.; ^18^ U.S. Animal Health Association.; ^19^ Louisiana State University School of Veterinary Medicine, Veterinary Clinical Sciences.; ^20^ Food and Drug Administration Center for Veterinary Medicine.; ^21^ AVMA Council on Public Health.; ^22^ Department of Pathobiological Sciences, University of Wisconsin-Madison School of Veterinary Medicine.

## Table of Contents

Introduction.......................................................................304
Background.........................................................................305
  I. Rodents and other small mammals..........................................309
  II. Reptiles (turtles, snakes, and lizards).................................312
  III. Fish, reptiles, amphibians (frogs, toads, salamanders, and newts) and invertebrates that live in aquatic environments.............................................................................316
  IV. Backyard poultry........................................................318
  V. Feeder animals...........................................................321
Recommendations....................................................................323
  I. Overview.................................................................323
  II. General recommendations for all partners................................324
    A. Awareness and education (*suggested messaging for NTP owners and the general public*)................................................................................................324
    B. Handwashing..............................................................328
    C. Antimicrobial use, resistance, and stewardship...........................329
    D. Occupational health......................................................331
  III. Partner–specific recommendations................................334
    A. Industry: breeders, distributors, retailers..............................334
    B. Human healthcare and veterinary care providers: a One Health approach....337
    C. Industry groups, associations, and other affiliated organizations........338
    D. Government agencies......................................................338
  IV. Summary.................................................................339
  V. Acknowledgments..........................................................339
Bibliography.......................................................................340
Appendices.........................................................................348
    A. NTP literature review and NORS/ACOSS data request results................348
    B. Table of selected zoonoses...............................................349
    C. Glossary.................................................................354
    D. Industry layout..........................................................355
    E. Handwashing..............................................................356
    F. Guidelines for animals in schools, childcare settings, and long-term care and assisted living facilities.......................................................................................357
    G. Selected recommendations, standards, guidelines, and regulations for non-traditional pet species..........................................................................................358
    H. Selected educational resources...........................................360

## Introduction

As people and a wide variety of animal species have increasingly close contact in diverse settings, guidance on preventing zoonotic diseases, caused by pathogens that spread between animals and people, is urgently needed. According to data (Annual Report of Animal Contact Outbreaks, 2021; Reptiles and Amphibians, 2022; Salmonella from Small Mammals, 2021; US Outbreaks of Zoonotic Diseases Spread between Animals & People, 2021) from the Centers for Disease Control and Prevention (CDC), three major groups of animals have repeatedly been associated with local, regional, and national outbreaks of zoonotic diseases in people in the United States: rodents, backyard poultry, and reptiles. This Compendium presents information on these and other non-traditional pet (NTP)* animal species associated with a high risk of zoonotic disease transmission in any setting. Other animal species covered in this Compendium include non-rodent small mammals (e.g., hedgehogs, ferrets), amphibians (e.g., African dwarf frogs), and other aquatic species (e.g., fish, coral) that are less frequently linked to illness or outbreaks, but nonetheless pose a risk of zoonotic disease transmission.

Many zoonotic disease exposures occur at home through direct or indirect contact with pets, agricultural animals, or feeder animals. Zoonotic diseases also affect workers employed in various segments of the pet industry, including animal breeders, pet store and other retail employees, and pet importers and distributors, as well as volunteers working closely with animals. This Compendium will provide guidance on addressing the zoonotic disease risks related to NTPs that are specific to these groups and settings, which may be different from those for other settings, populations, and animals. For guidance on preventing zoonotic diseases associated with animals in public settings (e.g., agricultural fairs, educational farms, petting zoos, schools, and other public venues), see the *Compendium of Measures to Prevent Disease Associated with Animals in Public Settings* and the *Compendium of Measures to Control* Chlamydia psittaci *Infection among Humans (Psittacosis) and Pet Birds (Avian Chlamydiosis), 2017*. For guidance on preventing occupational exposures to veterinary professionals see *Compendium of Veterinary Standard Precautions for Zoonotic Disease Prevention in Veterinary Personnel*.

*For the purposes of this document, the animals in this Compendium will be referred to as “non-traditional pets,” even though some of the animals listed are categorized as food-producing animals or livestock.

Recommendations and best practices in this Compendium were developed using a One Health approach with the goal of preventing zoonotic disease transmission between NTP species and people and reducing zoonotic disease risks in environments with animals and people (One Health, 2022). The intended audience for these recommendations includes employers, animal workers and leaders in the pet industry, NTP owners, human and animal healthcare professionals, public health officials, animal health officials, and others involved in controlling disease and reducing health risks.

**ONE HEALTH** means a collaborative, multisectoral, and transdisciplinary approach—working at the local, regional, national, and global levels—with the goal of achieving optimal health outcomes that recognize the interconnection between people, animals, plants, and their shared environment (One Health, 2022).

## Background

### Non-traditional pet (NTP) ownership in the United States

Pet ownership in the United States is increasing, with approximately 57–70% of households owning one or more pets (AVMA Pet Ownership and Demographics Sourcebook, 2017; “Pet Industry Market Size,” 2021) The majority of pets in the United States are dogs and cats; however, ownership of companion animal species other than dogs and cats, defined here as “non-traditional pets” (NTPs), is increasing. For the purpose of this document, these animals will be referred to as NTPs, even though some of the animals listed are categorized as food-producing animals or livestock. NTPs may include those species generally thought of as wildlife, species that are imported or rare in the United States, or livestock and poultry not typically kept as pets such as backyard poultry, which are not considered pets in most states. The number of backyard poultry owners climbed 23% in five years, with 1.1% of all US households owning backyard poultry in 2016 (AVMA Pet Ownership and Demographics Sourcebook, 2017). During the COVID-19 pandemic, agricultural stores and media outlets reported record purchasing of poultry during 2020 for egg and meat consumption and to have as pets (Nichols et al, 2021). Ownership of NTP species is increasing at an annual rate of 4% (“Pet Industry Market Size,” 2021). More than 13% of US households owned an NTP at the end of 2016, a 25% increase from 2011 (AVMA Pet Ownership and Demographics Sourcebook, 2017).

**Non-Traditional Pet (NTP) species covered in this Compendium:**

Rodents and other small mammals

Backyard poultry

Reptiles

Amphibians

Aquatic species

Pet ownership is associated with mental and physical health benefits in people (Allen et al, 2001; Baun and Mccabe, 2003; Carr et al, 2020; Ein et al, 2018; Kertes et al, 2017;^,^Maugeri et al, 2019). Pets can increase feelings of well-being, encourage more active lifestyles, and play a role in forming social support networks (Arhant-Sudhir et al, 2011; Wood et al, 2015). According to the American Heart Association, pet ownership, particularly dog ownership, may be associated with decreased cardiovascular disease risk (Levine et al, 2013). Asking about pets during a medical exam was shown in a recent study to have positive effects on practice and patient-provider relationships (Hodgson et al, 2017). The National Association of State Public Health Veterinarians (NASPHV) and the Centers for Disease Control and Prevention (CDC) recognize the many benefits of pets and support safe, healthy, and responsible pet ownership. Responsible pet ownership can prevent pet injury to people, reduce animal stress, and improve animal health. This includes avoiding impulsive decisions when selecting a pet, researching what the pet needs to be healthy, choosing a pet appropriate for a particular household, and providing appropriate husbandry, food, housing, handling, veterinary medical care, and socialization for the pet to keep it healthy. Responsible pet ownership also includes preventing zoonotic disease transmission from people to pets; it is important to separate sick people from pets to avoid transmission of germs such as human (seasonal) influenza viruses or severe acute respiratory syndrome coronavirus 2 (SARS-CoV-2) (Animals and COVID-19, 2022).

In addition to the individual benefits, pet ownership benefits local, regional, and national economies. The amount spent on pets, pet supplies, veterinary medical care, and other services is increasing, with an estimated $97.1 billion spent in 2019 and $103.6 billion spent in 2020 (“Pet Industry Market Size”, 2021). The increasing trends in NTP ownership and spending are expected to continue over the next several years (“Pet Industry Market Size”, 2021).

### NTPs and zoonotic diseases

Contact with NTPs increases the risk of exposure to zoonotic pathogens (i.e., pathogens spread between animals and people). NTPs can appear healthy while carrying zoonotic pathogens, putting pet owners and others at risk, especially those in high-risk groups (People at Higher Risk for Illness from Animals, 2022). NTPs should be recognized as potential primary or intermediary hosts in novel zoonotic pathogen emergence. As new NTP species become popular and the number of imported pets from around the globe increases, people and animals are at increased risk for emerging or reemerging zoonotic diseases, including those that have not previously been found in the United States (Smith et al, 2012). People can infect NTPs with zoonotic diseases as well, such as seasonal influenza virus transmission from humans to ferrets (Belser et al, 2018).

### Transmission of zoonotic pathogens

Most pathogens described in this Compendium do not cause illness in the animal host. NTPs can appear healthy while carrying a pathogen that can cause illness in people, putting the people in contact with them at risk. Transmission and infection mechanisms of some zoonotic pathogens associated with NTPs are not well-described. A better understanding of routes of transmission and time periods of shedding (the time the animal is infectious and spreading the pathogen in its environment), immune responses, and prevention strategies (e.g., vaccination) for people and animals would enhance the ability to control zoonotic pathogens in NTPs and protect the health of people who come into contact with these species.

**People at higher risk of illness**

Anyone can become sick from a zoonotic disease, including healthy people. However, some people are at higher risk for serious illness or death from these infections. People at higher risk of illness include:

- Children younger than 5 years old
- Adults 65 years and older,
- People with weakened immune systems due to illness (e.g., HIV/AIDS, cancer, diabetes, liver disease, kidney disease, and multiple sclerosis)
- People taking certain medications including those that weaken the immune system (e.g., steroids, cancer chemotherapy, and drugs used to treat autoimmune diseases like rheumatoid arthritis or psoriasis)
- Pregnant people

Zoonotic pathogens associated with NTP species and other animals can be transmitted by direct or indirect animal contact. Direct transmission of zoonotic pathogens can occur when people pet, touch, or kiss animals via accidental ingestion of feces from contaminated fur, feathers, scales, or spines; contaminated saliva from feeding an animal by hand or being licked by animals; or contact with other body fluids (Daly et al, 2017). Direct transmission of zoonotic pathogens can also occur via animal bites and scratches. Though rare, rabies, tetanus, and other highly pathogenic infections such as infections with *Streptobacillus moniliformis* (rat bite fever), *Francisella tularensis* (tularemia), or *Pasteurella* can be associated with NTP bite and scratch injuries, depending on the NTP species involved (Daly et al, 2017). Some NTP species such as venomous arthropods (e.g., scorpions and centipedes), arachnids (e.g., spiders), reptiles, amphibians, and fish can cause envenomation through stings, bites, or other contact. NTPs may also carry parasites (e.g., ticks, fleas) that may transmit vector-borne diseases to people (Daly et al, 2017; Mendoza-Roldan et al, 2020; Smith et al, 2012).

**Common risk factors that lead to illness associated with NTP species:**

- Not washing hands after handling animals or their food,waste, habitats, or other supplies (e.g., toys, leashes)
- Engaging in risky forms of contact (snuggling, kissing, or holding near face)
- Eating or drinking around NTPs
- Cleaning habitats in the kitchen or in other food preparation areas that leads to cross-contamination of kitchenware used for people (e.g., baby bottles)
- Allowing animals to roam freely in the home or in food preparation areas

Zoonotic disease transmission can also occur indirectly via contact with a surface or environment contaminated by an animal's urine, feces, blood, saliva, nasal secretions, or other body fluids; via contamination of food preparation areas and items; or less commonly through infectious droplets or aerosols (Daly et al, 2017). In multiple outbreaks of human illness linked to NTP species, contamination of food preparation surfaces by the animals themselves, contaminated items (e.g., by cleaning animal dishes or habitats in kitchen sinks), and allowing NTP species to freely roam indoors have been identified as important preventable risk factors for human infection (Gaffga et al, 2012; Loharikar et al, 2013; Loharikar et al, 2012; Zarecki et al, 2013).

Proper handwashing can interrupt many of the common routes of zoonotic disease transmission. Handwashing is discussed further in the Recommendations section of this document and Appendix E. Handwashing.

### Transmission of zoonotic diseases from people to animals

The COVID-19 pandemic has shown that diseases in wildlife, such as coronavirus infection in animals, can spread from animals to people and then between people (Animals and COVID-19, 2022) Some pathogens, including influenza viruses (Influenza in Animals, 2018) and SARS-CoV-2, can also spread from people to animals in some situations, especially during close contact. This is a public health concern because of the potential for pathogen mutation or reassortment that can result in new strains that may be capable of adapting to new hosts or ecologic environments (Ghai et al, 2021). As of September 2021, farmed mink, zoo and aquaria animals (including large cats, otters, gorillas, hippos), companion animals (including dogs, cats, and a ferret), and wildlife (including free ranging white-tailed deer (Animals and COVID-19, 2022) in the United States have been naturally infected with SARS-CoV-2 as a result of close contact with people with COVID-19 including farm workers, caretakers or keepers, pet owners (Cases of SARS-CoV-2 in Animals in the United States, 2022). Wild white-tailed deer have been confirmed to be infected with SARS-CoV-2 in several US states (Animals and COVID-19, 2022). Ferrets are susceptible to influenza viruses, including human seasonal influenza A viruses, and avian and swine influenza A viruses (Ferrets, 2021). People can protect pets and other animals by avoiding contact with animals including pets, livestock and other production animals, and wildlife when they have any illness (e.g. influenza virus), or are suspected or confirmed to be infected with SARS-CoV-2.

Transmission of zoonotic pathogens associated with NTP contact can occur in settings inside and outside the home. Transmission in public settings, such as petting zoos and other public animal exhibits, is addressed in the *Compendium of Measures to Prevent Disease Associated with Animals in Public Settings, 2017* (Daly et al, 2017).

**Special contact settings with non-traditional pets**

People may have contact with non-traditional pets in a variety of settings. Here are some examples (Daly et al, 2017):

- **Home** - contact with pets; farm animals; breeding animals; feeder animals
- **Group settings** - classrooms; child care facilities; long-term care facilities
- **Retailers** - retail pet stores; agricultural and feed stores;online sales; hobby or trade conventions; live animal markets and auctions; flea markets; street vendors; souvenir shops; roadside stands; toy stores or other retail stores
- **Special events -** seasonal events (e.g., Easter egg hunts); swap meets; hobbyist meetups; rat races; barn-hunting; birthday parties
- **Exhibitions** - petting zoos; agritourism venues; animal exhibition shows (e.g., those put on by 4H or National FFA Organization); animal shelter/rescue fundraising events; performing animal shows
- **Occupational settings** - veterinary clinics; commercial and in-home breeders; distributors; retail workers at pet, agricultural, or other stores where NTPs are sold; diagnostic laboratories; mail carriers; animal transporters; animal shelters and rescue organizations; and volunteers in those workplaces

NTPs can also pose **occupational health risks**. Zoonotic disease outbreaks linked to NTPs in recent years have affected workers in a variety of occupations, including workers at animal breeding and rearing facilities; those involved in animal distribution (e.g., postal workers); workers at retail locations such as pet stores and feed stores; and veterinary clinic staff. Examples of worker-related illnesses related to NTPs include rat bite fever in a pet shop employee (Shvartsblat et al, 2004); lymphocytic choriomeningitis virus (LCMV) infection in employees of a feeder-rodent operation (Knust et al, 2014); Seoul virus infection in the owners of a rat-breeding facility (Kerins, 2019) ; and monkeypox infections in veterinarians, pet shop employees, and animal distributors (Monkeypox in the United States, 2022; Croft et al, 2007). Postal workers, feed store employees, and others involved in the distribution and selling of backyard poultry have acquired *Salmonella* infections (Behravesh et al, 2014). Additional occupational health considerations and recommendations will be covered throughout this Compendium.

### Summary of identified outbreaks and case reports associated with NTP species in the United States from 1996 through 2017

CDC conducted a literature review to characterize the number of outbreaks, case reports, and types of pathogens associated with NTP species in the United States from 1996 through 2017. Key search terms included scientific pathogen names (e.g., “*Salmonella”*), NTP species of interest (e.g., “poultry,” “turtles”), “pet ownership,” “outbreak,” and other related terms (Appendix A. NTP literature review and NORS/ACOSS data request results). In addition to the literature review, outbreak reports were retrieved via data request from CDC's National Outbreak Reporting System (NORS), a web-based platform launched in 2009 for local, state, and territorial health departments to report to CDC domestic outbreaks of foodborne and waterborne disease as well as outbreaks of enteric disease transmitted by contact with environmental sources, infected persons or animals, or unknown modes of transmission (National Outbreak Reporting System (NORS), 2019).

Animal contact outbreaks reported through NORS are captured by the Animal Contact Outbreak Surveillance System (ACOSS) (Animal Contact Outbreak Surveillance System, 2020). Reports retrieved from ACOSS through NORS were reviewed in detail if they described outbreaks (2 or more cases) that occurred during 2009–2017 that were associated (suspected or confirmed) with contact with an identified NTP species covered in this Compendium. Outbreaks or case reports that reported multiple species were included so long as there was an NTP species reported. Outbreaks and case reports retrieved from the literature search and outbreak-associated illnesses from ACOSS that met the criteria for further review were recorded in a database for analysis. If an outbreak or case report appeared in both the literature and in ACOSS, then only the data from ACOSS were recorded in the database because they are updated in real time as reported by the jurisdiction. ACOSS only captures enteric illnesses associated with animal contact, whereas the literature review included enteric illnesses and non-enteric illnesses.

*Salmonella* and NTP species

**The pathogen most commonly identified across all NTP-associated outbreaks and case reports were *Salmonella* bacteria.**

**What is *Salmonella*?**

*Salmonella* are zoonotic bacteria found in the intestinal tract of many healthy animals, including rodents, reptiles, amphibians, and backyard poultry (Behravesh et al, 2014)

**Why is it important?**

*Salmonella* is well-described as the **most common** source of bacterial infection in people during outbreaks associated with NTPs. Salmonellosis linked to all animal contact is estimated to cause >100,000 illnesses annually (Hale et al, 2012).

**Findings**

*Salmonella* bacteria caused 81% of 243 NTP-associated outbreaks and case reports during 1996–2017.

- Rodents & other small mammals (17 outbreaks)
- Reptiles (62 outbreaks, 6 case reports)
- Fish & amphibians (8 outbreaks)
- Backyard poultry (105 outbreaks)

A total of 9,798 individual NTP-associated salmonellosis cases were detected.

112 *Salmonella* outbreaks were multistate

**CDC estimates that for each human *Salmonella* infection reported, 29 additional cases occur**.

Using these methods, a total of 243 outbreaks and case reports were identified that described disease linked to contact with NTP species. These were largely attributable to four major pathogens: *Salmonella* bacteria (Behravesh et al, 2014; Hale et al, 2012), lymphocytic choriomeningitis virus (LCMV), *Streptobacillus moniliformis* bacteria (rat bite fever), and Seoul virus (a member of the hantavirus family). When combined, the literature review and ACOSS reports described 9,875 human illnesses attributable to these four pathogens, with 1,461 (15%) occurring in children younger than 5 years old. A total of 1,752 (18%) were hospitalized and 33 (0.3%) deaths were associated with these illnesses. Small numbers of human illnesses linked to contact with NTP species and associated with other pathogens, including *Campylobacter* species, monkeypox virus, and *Francisella tularensis*, were also captured in the literature review.

A limitation of this literature review and ACOSS data request is that reporting through NORS is voluntary and agencies may experience limited ability to investigate and report these outbreaks, therefore infections with these pathogens are likely underreported (Hale et al, 2012). For example, national reporting is not required for rat-bite fever (Kache et al, 2020) or LCMV (“Notes from the Field: Congenital Lymphocytic Choriomeningitis,” 2021), and infections are required to be reported by few states. ACOSS only includes enteric pathogens associated with animal contact, so does not include some of the pathogens captured in the literature review.

### Practicing antimicrobial stewardship in NTPs helps prevent antimicrobial resistance

Antimicrobial resistance (AMR) is an increasingly recognized One Health issue affecting both human and animal health, including NTP species. Antimicrobials are valuable tools used to fight bacterial, viral, parasitic, and fungal infections in both animals and people, but improper use or overuse of these medications can create drug resistance and reduced effectiveness of antimicrobials for the treatment of animal and human illnesses (Antibiotic/Antimicrobial Resistance, 2021). CDC estimates that more than 2.8 million antibiotic-resistant and antifungal-resistant infections occur in the United States each year and more than 35,000 people die as a result (Antibiotic/Antimicrobial Resistance, 2021). When bacteria, viruses, and fungi are exposed to antimicrobials, strains that are able to survive can multiply. These strains can then share the mechanisms or genes that confer resistance or resistance determinants with other organisms that may not have been exposed to the drug. Antimicrobial-resistant bacteria may spread between colonized or infected people and animals. Contact with the environment (e.g., water, soil) may also play a role in the spread of antimicrobial-resistant bacteria. AMR is one of the biggest threats currently facing public health and is an important One Health issue requiring collaboration across disciplines and sectors (Antibiotic/Antimicrobial Resistance, 2021; Antimicrobial Resistance, 2021; World Health Organization, 2017). In CDC's 2019 *Antimicrobial Resistance Threats in the United States* report (2019 AR Threats Report, 2019), drug-resistant *Salmonella* and drug-resistant *Campylobacter* strains were listed as serious public health threats that require prompt and sustained action. Drug-resistant *Salmonella* and *Campylobacter* strains associated with NTPs and other animals also pose a threat to human health. Recent outbreaks in NTP species highlight the importance of antimicrobial stewardship for human and animal health (Outbreak of Salmonella Infections Linked to Backyard Poultry, 2020).

Antimicrobial stewardship refers to a framework for judicious antimicrobial use in both people and animals, including NTPs (Antimicrobial Stewardship Definition and Core Principles, n.d.). All participants in an animal's care throughout its lifespan, including breeders, distributors, retailers, pet owners, and veterinarians, play essential roles in implementing antimicrobial stewardship and preventing the spread of resistant bacteria. This includes providing appropriate housing, a nutritious diet, routine and emergent veterinary care, and infection prevention measures. The Federal Task Force for Combating Antibiotic-Resistant Bacteria, established in 2014, is co-chaired by the US Departments of Health and Human Services (HHS), Agriculture (USDA), and Defense (DOD). The Task Force released the *National Action Plan for Combating Antibiotic Resistant Bacteria (CARB), 2020–2025* describing the actions the federal government will take to reduce the impact of antibiotic resistance using a One Health approach (National Action Plan for Combating Antibiotic Resistant Bacteria 2020–2025, 2020). The Food and Drug Administration (FDA) Center for Veterinary Medicine's (CVM) 5-year plan to support antimicrobial stewardship in veterinary settings includes measures to improve stewardship for both food-producing and companion animals (“FDA Releases Draft Guidance,” 2021). These cooperative efforts will help to ensure that these important medications continue to be available and effective for both people and animals. Antimicrobial stewardship will be discussed further in the Recommendations section of this document.

### A One Health approach is necessary to prevent zoonotic diseases associated with NTPs

Prevention and control of zoonotic diseases and antimicrobial resistance requires a One Health approach. This approach includes identifying of host, pathogen, and environmental factors that influence the risk of disease; promoting animal husbandry and welfare; understanding the industry operations and regulatory frameworks; and developing interventions and educational campaigns that can be implemented in the many settings, at home or away from home, where people come in contact with NTPs. Partners involved in a One Health approach to preventing NTP zoonoses are government agencies including public health and animal health officials; NTP industry including breeders distributors,and retailers including pet and agricultural industry employers and employees; industry groups, professional associations, and affiliated non-governmental organizations; human and animal healthcare providers; nontraditional pet owners; and other relevant One Health partners.

### Contact between wildlife and NTPs can contribute to the emergence of zoonotic diseases

Several of the zoonotic diseases referenced in this Compendium have a confirmed or suspected wildlife source that led to disease first in NTP species and then in people. For example, comingling of wfild African mammals with prairie dogs in the NTP retail distribution system led to a human monkeypox outbreak in 2003 which marked the first time that human monkeypox was reported outside of Africa (Monkeypox in the United States, 2022); contact between wild and captive-bred rodents was suspected as a potential source of human outbreaks of Seoul virus in the United States (Kerins, 2019); and contact between captive-bred rodents and wild rodents was associated with multiple human lymphocytic choriomeningitis virus (LCMV) outbreaks (Amman et al, 2007; Biggar et al, 1975; Deibel et al, 1975; Gregg, 1975; Rousseau et al, 1997; Knust et al, 2014). During the 2014–2015 highly pathogenic avian influenza (HPAI) outbreak in the United States, the virus was detected on the premises of twenty-one backyard flocks in addition to 211 commercial premises (Final Report for the 2014–2015 Outbreak of Highly Pathogenic Avian Influenza (HPAI) in the United States, 2016). Migratory birds from the Pacific Flyway were the most likely source of introduction of HPAI, causing the largest animal health incident in United States history to date (Final Report for the 2014–2015 Outbreak of Highly Pathogenic Avian Influenza (HPAI) in the United States, 2016). These examples illustrate the importance of keeping NTPs and other pets separated from wildlife to prevent transmission of disease to pets and people. To further avoid potential wildlife disease transmission, it is also important that people do not keep native wildlife as pets (e.g., squirrels, racoons, skunks, opossums, foxes, mink).

To prevent transmission of zoonotic diseases, people should **not** keep wildlife (e.g., racoons, opossums, foxes, mink) as pets and should keep NTPs and other pets **away** from wildlife.

### Compendium organization and audience

This Compendium provides information and practical recommendations on preventing and controlling zoonotic diseases in NTPs using a One Health approach. Recommendations are targeted to partners at each stage of the NTP lifecycle.

For the purposes of this Compendium, audience sectors have been defined as follows* (Appendix C. Glossary):

- **Industry:**
  - **Breeder:** A breeder is any operation, regardless of size, that maintains a population of animals for the purpose of commodification (e.g., sale, trade, or swap) of the animals or their offspring.
  - **Distributor/importer:** A distributor is any operation, regardless of size, that purchases animals from breeders, other domestic distributors, or international sources and houses them on a short-term basis for distribution to retailers and direct sales to owners.
  - **Retailer:** A retailer is a commercial entity that purchases animals from breeders, distributors, and/or importers for subsequent sale at a physical (“brick-and-mortar”) storefront or via the internet. Some retailers are increasingly also selling directly to owners via the internet or operating exclusively through online sales.
- **Non-traditional pet owner:** An NTP owner is a person who purchases or otherwise acquires non-traditional animals for the purpose of keeping them as pets.
- **Human healthcare and veterinary care providers:**
  - **Human healthcare providers**: Physicians, nurses, and allied health professionals.
  - **Veterinary care providers**: Veterinarians, veterinary technicians (including veterinary nurses or pet nurses), clinic assistants, kennel staff, and other support staff.
- **Government agencies:** Government agencies include relevant local, state, tribal, or federal public health, animal health, agriculture, wildlife, or environment agencies.
- **Other relevant One Health partners:** Other relevant One Health partners include industry groups, professional associations, affiliated non-governmental organizations, and others associated with managing NTP species in public settings such as schools, childcare facilities, long-term care and assisted living facilities, zoos, and aquaria.

*Occupational health considerations apply for those audience sectors that are also employers where employees may have direct or indirect contact with NTPs (e.g., retailers, distributors, veterinarians, and others).

### I. Rodents and other small mammals

#### Overview

In 2017, 5 million small mammals were kept as pets in the United States (“Pet Industry Market Size,” 2021). Among small mammal pets, the majority are rodents such as rats, mice, hamsters, gerbils, and guinea pigs (Pet Ownership and Demographics Sourcebook, 2017). Rodents are popular pets due to their small size, portability, and relative ease of care (“Selecting a pet rodent,” n.d.).

**Small mammal species including:**

○ Rodents (rats, mice, hamsters, gerbils, guinea pigs, prairie dogs)
○ Hedgehogs
○ Sugar gliders
○ Rabbits
○ Ferrets

A total of 434 documented human illnesses associated with small mammal contact occurred from 1996 through 2017 according to case reports and outbreak data from the literature review and ACOSS data. *Salmonella* bacteria were the pathogens identified in most illnesses, but cases of infections with LCMV, monkeypox virus, Seoul virus, and *Streptobacillus moniliformis* (rat bite fever) were also identified. Zoonotic *Franciscella tularensis* (tularemia) and *Streptococcus* infections were rarely reported.

#### Common risk factors for illness from rodents and other small mammals

In addition to the common risk factors among all NTP species (for more information, refer to the Background section of this document on transmission of zoonotic pathogens), additional risk factors specific to rodents and other small mammal species include bites or scratches from the animals themselves and exposure to contaminated surfaces (“Notes from the Field: Fatal Rat-Bite Fever in a Child,” 2013; Shvartsblat et al, 2004; Small Mammals, 2019). Exposure to contaminated urine, feces, blood, saliva, nasal secretions, or other body fluids, or material within the animals' habitat can transmit bacterial or viral infections from these animals (Small Mammals, 2019).

In a 2003 monkeypox virus outbreak associated with infected prairie dogs, risk factors identified included touching an infected animal or receiving a bite or scratch that broke the skin. Other important risk factors were cleaning the habitat or touching or handling the bedding of an infected animal.

In areas where plague (*Yersinia pestis* infection) is endemic, handling wild-caught prairie dogs, wild rabbits, rodents, and other small mammals (von Reyn et al, 1976) is a risk factor for human infection, which may also be acquired via flea bites or other types of contact (Campbell et al, 2019; Melman et al, 2018).

#### Bites and zoonotic disease transmission

Small mammal NTP species with outdoor access should be protected from contact with wildlife to prevent disease transmission.

Bites from rodents and other small mammals are an important mechanism of zoonotic disease transmission. Bites from these animals generally do not cause extensive injury, but they are common and can transmit many different bacterial or viral pathogens (Kache et al, 2020; Langley et al, 2014). The bacterial species most commonly found in small mammal bite wounds include *Pasteurella multocida*, *Staphylococcus aureus*, *Pseudomonas* species, *Streptococcus viridans*, and other anaerobes (Iyengar et al, 2013). Additional considerations include rarer infections that can cause severe illness and even death (e.g., infections with *Clostridium tetani, Francisella tularensis,* hantaviruses, lymphocytic choriomeningitis virus, *Streptobacillus moniliformis, Yersinia pestis).*

Rabies is uncommon in pet rodents and rabbits (Fitzpatrick et al, 2014). Rabies testing and prophylaxis are not routinely recommended for provoked bites from apparently healthy pet rodents and rabbits but should be considered on a case-by-case basis. Rabies has been documented in rabbits and guinea pigs that had outdoor access or were housed outside in areas with epizootic raccoon variant rabies (Fitzpatrick et al, 2014; Eidson et al, 2005). Small mammal NTP species with outdoor access should be protected from contact with wildlife (Compendium of Animal Rabies Prevention and Control, 2016). Small mammal NTP species with outdoor access should be protected from contact with wildlife (Compendium of Animal Rabies Prevention and Control, 2016). Off-label use of rabies vaccination for small mammal NTP species housed outdoors or with outdoor access may be considered under the guidance of a veterinarian (Compendium of Animal Rabies Prevention and Control, 2016). If evidence of wildlife contact is detected, local or state public health authorities should be consulted for further guidance.

Ferrets, which are distinct from other small mammal NTPs because they are predators, are also capable of transmitting rabies (Compendium of Animal Rabies Prevention and Control, 2016). NASPHV recommends that ferrets are vaccinated for rabies; recommendations for rabies vaccination of ferrets and for management of ferrets that have bitten people are available in the *Compendium of Animal Rabies Prevention and Control*. Ferrets can inflict severe injury on infants and young children, with multiple reports describing unprovoked attacks to the face and head and associated puncture wounds, lacerations, and/or tissue loss (Applegate and Walhout, 1998; Ferrant et al, 2008; Kizer and Constantine, 1989; Paisley and Lauer, 1988; Patronek and Slavinski, 2009). NASPHV recommends that ferrets not be kept as pets in households with children younger than 5 years old. In some states and US territories, ferret ownership is illegal, and in others, rabies vaccination is compulsory (State Rabies Vaccination Laws, 2021).

#### Zoonotic pathogens

##### I. *Salmonella*

*Salmonella* bacteria can spread through many ways such as contact with feces of infected small mammals or contaminated surfaces. A diversity of NTP species were identified in the literature and ACOSS data, including rats, mice, guinea pigs, and hedgehogs, with multiple outbreaks or case reports associated with each of these. Rodents were associated with over half of the small mammal salmonellosis illnesses identified (refer to the Background section of this document on *Salmonella* for more detailed information on *Salmonella*).

***Salmonella* infections associated with rodents and other small mammals**

Seventeen reported outbreaks or case reports of human salmonellosis associated with rodent and other small mammal contact were reported during 1996–2017 resulting in:

- 391 illnesses
- 46 hospitalizations
- 1 death

Seven of these outbreaks or case reports were associated with live and frozen feeder rodents (Section V. Feeder Animals).

Most of the outbreaks identified in the CDC literature review and ACOSS data request were multistate (Appendix C. Glossary), however, this may reflect the type of illness reports that get investigated and published versus a true reflection of frequency. Single state outbreaks or individual cases may not be as likely to be investigated or published. The distribution pathways of these species from breeder to distributor, retailer, and NTP owner affect geographic distribution of outbreaks (Appendix D. Industry layout).

##### II. Lymphocytic choriomeningitis virus (LCMV)

Lymphocytic choriomeningitis virus (LCMV) is a rodent-borne arenavirus and a rare cause of human illness. The primary reservoir for LCMV is the house mouse (*Mus musculus*) (Jay et al, 2005). Most human LCMV infections are associated with wild rodent exposure (Charrel et al, 2006; Foster et al, 2006; Talley, 2019); however, human LCMV infection has also been associated with exposure to pet or commercial rodent species that have been exposed to wild rodents, particularly hamsters (Amman et al, 2007; Biggar et al, 1975; Deibel et al, 1975; Gregg, 1975; Rousseau et al, 1997) and feeder mice (Knust et al, 2014). LCMV has also been detected in other pet rodent species, such as guinea pigs (Amman et al, 2007).

**LCMV illnesses associated with rodents**

Four reported outbreaks and 7 case reports of LCMV associated with mice and pet hamsters were reported during 1996–2017 resulting in:

- 32 human illnesses
- 12 deaths

Exposures occurred in private homes, schools, and commercial breeding facilities.

Congenital infections with LCMV are a potential cause of birth defects in babies born to mothers exposed to LCMV during pregnancy (Barton et al, 1995; Wright et al, 1997).

***Occupational health risk:*** Exposures and illnesses have occurred in commercial rodent breeding facility employees and veterinarians.

Zoonotic transmission of LCMV can occur via rodent bites, direct contact with rodents and their urine, feces, saliva, bedding or nesting materials, or inhalation of aerosolized infectious materials in the environment (Jay et al, 2005).

LCMV infections in people can be asymptomatic or cause a mild febrile illness; in high-risk groups more severe infections can cause meningitis, encephalitis, myelitis, or acute hydrocephalus (Charrel et al, 2006; Wilson and Peters, 2014). Person-to-person transmission can occur via organ transplantation (“Brief Report: Lymphocytic Choriomeningitis,” 2008; Fischer et al, 2009; MacNeil et al, 2012) or from a pregnant person to their fetus (Barton and Mets, 2001). Congenital infections can result in fetal death or severe neurologic and/or ophthalmologic birth defects (Barton and Mets, 2001; Barton et al, 1995; Wright et al, 1997). Congenital infection with LCMV has been reported in the infant child of a veterinarian with direct occupational exposure to pet rodents and indirect home exposure to wild rodents (J. Brown, personal communication, Indiana Department of Health). Contact between wild and domestic rodents was suspected as a potential source of previous outbreaks of LCMV infections in the United States (Knust et al, 2014; Talley, 2019).

##### III. *Streptobacillus moniliformis* (the cause of rat bite fever)

Rat bite fever (RBF) is a rare bacterial disease transmitted by bites or scratches from colonized rodents, typically rats, although mice, gerbils, guinea pigs, and squirrels can also carry the bacteria that cause it (Infection in Animals, Rat-Bite Fever (RBF), 2019). *S. moniliformis* can also spread through exposure to rodent urine or saliva, or surfaces contaminated with these fluids, including water or food. Ingestion of *S. moniliformis* causes the disease Haverhill fever (Abdulaziz et al, 2006). Rats colonized with the bacteria may appear healthy, although some may develop arthritis, skin infections, pneumonia, and swollen lymph nodes. Clinical signs in people include fever and joint pain or rash that can progress to systemic involvement of the heart, liver, lungs, or brain and sepsis if untreated (Hryciw et al, 2018; Kache et al, 2020). Children and young adults are more commonly affected (“Notes from the Field: Fatal Rat-Bite Fever in a Child,” 2013) and infection is increasingly associated with pet rodent ownership (Kache et al, 2020). RBF surveillance is not routinely conducted in the United States, and analysis of nationwide administrative datasets suggests that it may occur more commonly than is reported (Kache et al, 2020). Further research is needed to characterize the true prevalence and associated risk factors (Shvartsblat et al, 2004).

**Rat bite fever illnesses associated with small mammals**

Seven reports of rat bite fever were identified in the literature review during 1996 – 2017, including:

- 29 human illnesses reported
- 5 deaths reported

Exposures occurred in private homes and a pet store.

***Occupational health risk:*** A pet store employee became sick and died after scratching himself on a cage that housed an infected rat (Shvartsblat et al, 2004).

##### IV. Hantaviruses (Seoul virus and others)

Seoul virus is an Old World hantavirus with worldwide distribution. It is carried and spread by rodents, specifically the brown or Norway rat (*Rattus norvegicus*), including pet and wild rat populations. People can become infected through contact with infected rodents or their urine, feces, saliva, or contaminated nesting materials (Kerins, 2019). Infection in people can progress to hemorrhagic fever with renal syndrome (HFRS) which can be fatal.

**Seoul virus illnesses associated with rodents**

Two case reports and 1 outbreak were identified in the literature review during 1996–2017, including:

- 19 human illnesses
- 1 death

**A 2016**–**2017 outbreak marked the first time Seoul virus in humans was attributed to pet rats in the United States** (Kerins, 2019)

- Associated with small, private breeders
- 31 facilities in 11 states with human or rat Seoul virus infections
- 17 human cases identified

***Occupational health risk:*** At least one patient owned and operated an in-home rattery in the 2016–2017 outbreak (Kerins, 2019).

Other hantaviruses include Sin Nombre virus (SNV), a New World hantavirus and rare cause of hantavirus pulmonary syndrome (HPS), a severe pulmonary disease associated with exposure to wild rodents (the SNV reservoir is the deer mouse *Peromyscus maniculatus*). SNV may be a concern in cases where wild rodents have contact with domestic rodents, such as those in breeding facilities. Contact between wild and domestic rodents is suspected as a potential source of previous outbreaks of Seoul virus in the United States (Kerins, 2019).

##### V. Monkeypox virus

Monkeypox is a rare viral disease usually found in central and western Africa (Baum, 2018). Monkeypox does not occur naturally in the United States, but cases have happened that were associated with international travel and importation of animals from places where the disease is more common. Monkeypox virus can infect rats, mice, rabbits, and prairie dogs, but may not cause clinical signs of disease in infected animals. A 2003 monkeypox outbreak in the United States highlights the effect that co-mingling wildlife and domestic animals can have on public health through the emergence of zoonotic diseases (monkeypox call out box) (Croft et al, 2007; Monkeypox in the United States, 2022; Reed et al, 2009). Exposures to infected prairie dogs occurred among several groups including 26 children attending in-home childcare, resulting in 5 confirmed infections (Kile et al, 2005). Healthcare workers attending to 3 patients were also exposed, with no cases reported.

**Monkeypox in the United States, 2003** (Croft et al, 2003; Monkeypox in the United States, 2022)

**Transmission of monkeypox virus via co-mingling of wildlife and domestic animals**

- **Wild mammals, including giant pouched rats (*Cricetomys* sp.), imported from Ghana introduced monkeypox virus to prairie dogs and other small mammals being sold as pets at a US distributor.**
- The prairie dogs were sold as pets before developing signs of infection.
**Some animals only had minimal signs of illness and recovered, while others died.**
  Signs of illness in prairie dogs included fever, cough, discharge from the eyes, and enlarged lymph nodes, accompanied by the development of lesions, lethargy, and not eating or drinking.
- All people infected with monkeypox became sick after contact with infected prairie dogs.
- **Certain activities** were more likely to lead to monkeypox infection – touching a sick animal or receiving a bite or scratch that broke the skin, cleaning the cage, or touching the bedding of a sick animal.

This outbreak resulted in 47 human cases of monkeypox, including 14 hospitalizations, in people who had contact with infected prairie dogs purchased as pets.

This outbreak highlights the potential public health threat posed by importation of wildlife for the US pet trade.

##### VI. Leptospira species

Leptospirosis is a bacterial disease that affects people and animals and is widespread globally (Leptospirosis, 2019). It is caused by spirochete bacteria of the genus *Leptospira*, with 22 known species divided into more than 300 serovars (e.g., *Leptospira interrogans* serogroup Icterohaemorrhagiae) (Boey et al, 2019). The bacteria can spread through the urine of infected animals including rodents, cattle, pigs, horses, dogs, and other wild animals) (Leptospirosis, 2019). Wild rats (*Rattus* species), especially the Norway/brown rat (*Rattus norvegicus*) and the black rat (*R. rattus*) are the most important sources of *Leptospira* infection in humans and animals as they are abundant in urban and peridomestic environments (Gaudie et al, 2008; Himsworth et al, 2013). *Leptospira* incidence increases after heavy rainfall and has been associated with natural disasters with large outbreaks occurring after hurricanes, typhoons, and floods (Himsworth et al, 2013). Rodents may be infected with the bacteria and show no clinical signs of disease (Leptospirosis, 2019). Infected animals may excrete the bacteria into the environment continuously or periodically over a few months up to several years (Leptospirosis, 2019). Infections in people have been associated with pet mice and rats (Day, 2016; Friedmann et al, 1973; Gaudie et al, 2008).

People can become infected with the bacteria through coming in to contact with the urine (or other body fluids, except saliva) of infected animals or contact with water, soil, or food contaminated with the urine of infected animals. Drinking contaminated water can cause infection. Infection can also occur through contact with contaminated water via cuts in the skin or mucous membranes in the nose, eyes, or mouth. People may be asymptomatic or have non-specific clinical signs including high fever, headache, chills, muscle ache, rash, or jaundice.

To help prevent leptospirosis infection in pets, it is important to keep wild rodents (rats, mice, or other animal pests) from mingling with pet rodents, and to keep rodent pest problems under control (Leptospirosis, 2019). People should not keep wild rats as pets.

##### VII. *Trichophyton*, *Microsporum*, and other dermatophytes

Dermatophytosis, colloquially known as ringworm, is a fungal skin infection that can be transmitted from small mammals to people either through direct contact or contaminated items such as bedding. Caused by a variety of genera, including *Trichophyton* and *Microsporum*, these dermatophytes are commonly associated with rabbits and guinea pigs (Cafarchia et al, 2012; Day, 2016; Donnelly et al, 2000; Kraemer et al, 2013). Infections in small mammals are normally mild or asymptomatic and consist of areas of crusting alopecia (hair loss). Hair loss and other skin lesions on animals should be properly diagnosed and treated by a veterinarian, and all pets in an affected household should be examined for ringworm. In addition, areas where the animal has spent time should be appropriately disinfected, if possible.

#### Distribution and purchase

People can purchase pet rodents from retail pet stores, online retailers, informal rodent swaps, or through private networks of hobbyists. Following the monkeypox outbreak, importation of African rodents was banned in 2003, but it is currently legal to sell captive-bred African rodents and prairie dogs in the United States. Wild-caught prairie dog pups are legal to sell as pets in many states. Some of these animals may be captured in areas where *Francisella tularensis* or *Yersinia pestis* are endemic. At least one identified prairie dog vendor sold wild-caught prairie dog pups advertised as captive-bred, potentially putting the NTP owner at increased risk of disease (Danielle Stanek, personal communication, Florida Department of Health).

See Appendix D. Industry layout for more detailed information on distribution and purchase of these animals.

#### Special exposure settings and occupational exposures

Potential occupational exposures should be considered for workers in laboratory settings, pet stores, commercial and in-home breeders, distributors, transporters, exhibitors of small mammals, veterinarians and veterinary medical staff, healthcare workers, and other relevant workers (Kile et al, 2005). Exposure to rodents and other small mammals may also occur in settings such as schools, childcare facilities, and long-term care facilities (Croft et al, 2007; Fleischauer et al, 2005). See Appendix F. Guidelines for animals in schools, childcare settings, and long-term care and assisted living facilities for information on preventing outbreaks associated with NTPs in these facilities.

#### Industry regulations

See Appendix G. Recommendations, Standards, and Guidelines for Non-Traditional Pet Species for more information on regulations related to small mammals, including information on the African Rodent Import Ban that was a result of the 2003 monkeypox outbreak.

### II. Reptiles (turtles, snakes, and lizards)

#### Overview

Reptile ownership has doubled over the last 20 years, with a 37% increase in the number of reptiles kept as pets in the United States from 2006 to 2016 (AVMA Pet Ownership and Demographics Sourcebook, 2017). Reptiles are popular pets because they are relatively quiet, unique, and can be entertaining to observe. They also typically require less space than other pets. However, many of these ectothermic (dependent on outside sources for heat) animals require specialized environments with specific light, temperature, and humidity requirements, and thus husbandry issues are common. Issues such as malnutrition, thermal burns, or ingestion of non-digestible materials such as bedding or substrate can lead to a variety of health problems in reptiles. Movement of animals (e.g., shipping from breeder to retailer or to the owner) can also cause stress that affects the health of the animal. These and other sources of stress may increase shedding of zoonotic pathogens such as *Salmonella* bacteria, which healthy reptiles can carry in their intestinal tracts.

More than 6 million turtles, snakes, lizards, and other reptiles were kept as pets in the United States in 2016, representing 2.9% of US households (AVMA Pet Ownership and Demographics Sourcebook, 2017)

Escape or unintentional release of reptiles (such as tegu lizards, pythons, Cayman crocodiles, monitor lizards, chameleons, geckos, and iguanas) and other pets not native to an area can result in establishment of breeding populations and potential negative impacts on native wildlife, people, and the environment (Albeck-Ripka, 2017; Florida's Non-Native Fish and Wildlife, n.d.; Hoyer et al, 2017, Pettit, 2018; Sweeney, 2018). Imported reptiles can also introduce tick species not naturally found in the United States (Albeck-Ripka, 2017; Burridge and Simmons, 2003; Pietzsch et al, 2005). Proper husbandry and consultation with a veterinarian are important to enhance animal health and welfare, and to reduce stress on pet reptiles.

#### Most common pet reptile species

This list represents the most common pet reptile species sold at medium and large chain pet retail stores. There are many more species that people buy and sell outside of these stores through independent retailers, reptile expos, and other venues (T. Edling, personal communication, American Humane/Pet Advocacy Network).

**Table:** 

Lizards	Snakes	Turtles/Tortoises
Leopard geckos (*Eublepharis macularius*) Bearded dragons (*Pogona* spp.) Blue-tongue skink (Tiliqua scincoides intermedia) Crested gecko (Correlophus ciliates) Green iguana (*Iguana iguana*) Veiled chameleon (*Chamaeleo calyptratus*) Fat tailed gecko (*Hemitheconyx caudicinctus*) Chinese water dragon (*Physignathus cocincinus*) Green anole (*Anolis carolinensis*) Savannah monitor (*Varanus exanthematicus*) Argentine black and white tegu (*Salvator merianae*) Uromastyx (*Uromastyx spp*.) Tokay gecko (*Gekko gecko*) New Caledonian giant gecko (*Rhacodactylus leachianus*)	Ball python (*Python regius*) Corn snake (*Pantherophis guttata*) King snake (*Lampropeltis* spp.) Hognose snake (*Heterodon nasicus*) Common boa constrictor (*Boa constrictor*) Rat snake (*Pantherophis obsoletus*) Green tree python (*Morelia viridis*) Rosy Boa (*Lichanura trivirgata*) Gopher Snake (*Pituophis spp.*)	Red-eared slider (*Trachemys scripta elegans*) Eastern box turtle (*Terrapene carolina carolina*) Western painted turtle (*Chrysemys picta bellii*) Map turtle (*Graptemys geographica*) Wood turtle (*Glyptemys insculpta*) African sideneck turtle (*Pelusios castaneus*) Peninsula cooter (*Pseudemys peninsularis*) Russian tortoise (*Testudo horsfieldii*) Greek tortoise (*Testudo graeca*) Leopard tortoises (*Stigmochelys pardalis*) Red-footed tortoise (*Chelonoidis carbonaria*) Indian star tortoises (*Geochelone elegans*) African spur-thighed tortoise (*Geochelone sulcata*) Hermann's tortoise (*Testudo hermanni*)

#### Common risk factors for illness from reptiles

In addition to common risk factors associated with all NTPs species (see Background section), there are additional risks specific to reptiles. People may incorrectly perceive some reptiles, including turtles, to be safe for children. Children may handle small turtles more easily and kiss, snuggle, and put the animals in their mouths, as is sometimes the case with turtles with a shell length under 4 inches/10cm. Small turtles pose a unique risk to children and are responsible for illness outbreaks each year. Water where reptiles are kept, such as aquariums, small bowls housing small turtles, swimming pools, sinks, or bathtubs, can also become heavily contaminated with pathogens (Harris et al, 2010). Please refer to the Aquatic animal section for more specific information on risk behaviors associated with reptiles and other animals living in an aquatic environment.

#### Zoonotic pathogens

##### I. *Salmonella*

The most common zoonotic disease acquired from pet reptiles is salmonellosis (Bosch et al, 2016). *Salmonella* bacteria can live in reptile gastrointestinal tracts, and generally do not cause signs of disease in reptiles (Bosch et al, 2016; Schlossberg, 2016). Reptiles can shed *Salmonella* bacteria intermittently in their feces, and some reptiles, such as snakes, may carry more than one serotype of *Salmonella* at a time (DuPonte et al, 1978; Goupil et al, 2012). Reptiles can acquire *Salmonella* strains in several was, including transovarially (transmission of the bacteria from the reptile to its offspring), via direct contact with other infected reptiles, or through contamination of their environment (Schlossberg, 2016; Schröter et al, 2006). Snakes and carnivorous reptiles may acquire *Salmonella* strains through food sources such as feeder animals, whether live or frozen, which can carry *Salmonella* bacteria (Section V. Feeder Animals) (“Notes from the Field: Infections with Salmonella,” 2012; “Salmonella Typhimurium Infections,” 2014).

***Salmonella* infections associated with reptiles:**

***Salmonella* bacteria are commonly spread between pet reptiles and people without direct contact.** Infections have been documented in infants and young children who never touched or interacted with a reptile but were infected through **cross contamination** in the household (Kiebler et al, 2020; “Multistate Outbreak of Human Salmonella,” 2008; “Reptile-Associated Salmonellosis,” 2003).

Sixty-eight outbreaks of *Salmonella* infections were associated with reptile contact identified from 1996-2017.

**Increasing public awareness about risk of *Salmonella* infection continues to be important (**Corrente et al, 2017)

In one study only 38% of interviewees were aware of risk of *Salmonella* infection from reptiles and 18% were aware of the infection risk from amphibians.

Transmission of *Salmonella* strains from reptiles to people can occur through direct contact with the animal and indirect contact with a contaminated environment. Indirect contact can occur through cross-contamination of the environment, including the home, where animals roam, or where people cared for or stored animal supplies. Contact with contaminated terrarium or aquarium water can occur with aquatic reptiles, where splashes from tanks and touching the water have led to *Salmonella* infections in infants and children (Bosch et al, 2016). Salmonellosis has also been acquired through swimming in a pool housing turtles with a shell length less than 4 inches (10.16cm) (“Multistate Outbreak of Human Salmonella Infections,” 2008). *Salmonella* infections in infants and children have resulted from cross contamination of sinks and bathtubs used to bathe pet reptiles or to clean reptile supplies and also to clean household items such as baby bot tles or to prepare infant formula or other food (Kiebler et al, 2020; “Reptile-Associated Salmonellosis,” 2003)

Multiple outbreaks of *Salmonella* infections in people have been associated with turtles marketed as *“Salmonella-*free” (Bosch et al, 2016; Harris et al, 2010; Walters et al, 2016).

*Salmonella* exposure and cross-contamination through food, water, other animals, or the environment makes raising *Salmonella*-free turtles impossible.

Certain husbandry practices can increase the risk of contracting a *Salmonella* infection from reptiles, including housing multiple reptiles in the same enclosure and infrequently changing the water in the habitat (Corrente et al, 2017). Terrarium substrates may act as a reservoir for *Salmonella* bacteria*,* which are extremely resilient in the environment (Winfield and Groisman, 2003). Viable *Salmonella* specimens have been isolated from dried reptile stool in cages 6 months after removal of the reptile and from aquarium water 6 weeks after removal of a turtle (Corrente et al, 2017; Mermin et al, 2004; Schlossberg, 2016). Washing eggs in a detergent solution may reduce *Salmonella* burden in turtle hatchlings (Mitchell et al, 2007), however, these animals may be subsequently exposed to *Salmonella* through food, water, other animals, and the environment, therefore they may be re-infected at any time (Harris et al, 2010). Using antibiotics to treat or wash eggs is never recommended because of the potential for development of antimicrobial resistance in turtles and their environment (Mitchell et al, 2007). Multiple outbreaks of *Salmonella* infections in humans have been associated with turtles marketed as *Salmonella*-free but were still shedding the bacteria (Bosch et al, 2016; Walters et al, 2016).

**Notable outbreaks associated with reptile contact and environmental contamination**

Below are selected outbreaks associated with small turtles, bearded dragons, and iguanas due to contamination of the home environment where the animals roam, where people cared for animals or stored their supplies, or through indirect contact. Visit CDC's *Salmonella* website for up-to date outbreak information.

**Small turtles**

- A 2010 turtle-associated outbreak of *Salmonella* infections resulted in 135 illnesses in 25 states and the District of Columbia*(“Multistate Outbreak of Human Salmonella Cotham,” 2014).
  - Approximately 45% of illnesses were in children younger than 5 years old.
  - At least 9 children attending 3 different **childcare facilities** acquired infection from index cases (all under 2 years old) who acquired their infections through turtle exposure.
  - Most of the turtles associated with illnesses had shell lengths of less than four inches in size and acquired from sources other than pet stores such as flea markets or street vendors.
- Four separate multistate outbreaks of *Salmonella* infections were linked to exposure to small turtles and their environments, such as tank water, between January 2015 and April 2016. Human S*almonella* infections were also documented in Luxembourg and Chile in connection with international small turtle exports from the United States during this time period (Gambino-Shirley et al, 2018).
  - Over 40% of the US cases were in children younger than 5 years old.
  - Nearly 70% of US cases were in people who reported Hispanic ethnicity.
  - Many people sickened from this outbreak were not aware of the risk reptiles pose for *Salmonella* infection.
  - An estimated 44% of turtles were purchased from flea markets, carnivals, or other transient vendors.

**Bearded dragons**

- A 2012–2014 outbreak of *Salmonella* infections involving 36 states with 166 human illnesses was linked to direct and indirect contact with pet bearded dragons (Kiebler et al, 2020; “Multistate Outbreak of Human Salmonella Cotham,” 2014).
  - An estimated 59% of illnesses were reported in children younger than 5 years old or younger.
  - At least 44 infections resulted in hospitalization.
  - **Interviews with patient guardians confirmed that in multiple instances of infant illnesses, the infant had no direct contact with the bearded dragon. Examples of indirect contact revealed holding a bearded dragon prior to breastfeeding, allowing the bearded dragon to roam in the household on the floor where the infant also crawled and keeping the bearded dragon terrarium on the same table used to change the infant's diaper**
  - *Salmonella* bacteria matching the outbreak strains were identified in multiple bearded dragon breeding facilities.

**Iguanas**

- *Salmonella* bacteria have spread between pet iguanas and infants who had **no direct contact** with the pets (“Reptile-Associated Salmonellosis,” 2003).

**These and other reports highlight:**

- The risk of **indirect transmission** of *Salmonella* from pet reptiles to their owners and others in the household, including infants, or workplace such as the childcare setting.
- The importance of **education** for all turtle owners on *Salmonella* infection risk and prevention measures, including in multiple languages.
- The need for **clear guidelines** for appropriate animal species in certain public settings, especially those places attended by people in high-risk groups (e.g., daycares, long-term care facilities, and schools).
- The ease with which zoonotic pathogens can move globally.
- The difficulty in conducting epidemiologic investigations and enforcing regulations when animals are purchased from non-traditional vendors (such as flea markets and street vendors).

##### II. Other zoonotic pathogens and causes of illness or injury to people

Less common potential zoonotic pathogens that can cause infections in reptiles include West Nile virus (WNV), *Aeromonas* spp., *Mycobacterium* spp., *Vibrio* spp., *Chlamydia* spp., *Burkholderia pseudomallei* (Melioidosis, 2021), and *Armillifer* (pentastomiasis) (Ebani et al, 2008). Reptiles have also been shown to be amplifying hosts for some arboviruses including chikungunya virus, WNV (crocodilians), and eastern equine encephalitis virus (snakes), with evidence of fecal shedding for WNV (Bosco-Lauth et al, 2018; Graham et al, 2012; White et al, 2011). There is some evidence that WNV might also spread from reptiles to other animals this way (Divers, 2020). Imported reptiles may harbor exotic ticks which can carry diseases that infect people. Four species of *Amblyomma* ticks, parasitizing lizards, and tortoises were introduced into Florida and were found to be infected with *Ehrlichia ruminantium*, which causes ‘Heartwater’ disease, and *C. burnetti*, the agent of Q fever (Mendoza-Roldan et al, 2020). Imported reptiles may also carry antimicrobial-resistant bacteria, contributing to the global spread of multidrug-resistant bacteria (Unger et al, 2017). Bites from reptiles can result in trauma and infection from a broad array of microbes, and some reptile species are venomous (Abrahamian and Goldstein, 2011). Large reptiles such as mature boa constrictors or crocodilians can pose a risk for severe injuries or even death if not housed or handled properly (Smith et al, 2012).

#### Antimicrobial-resistant pathogens are emerging in pet reptiles

Antimicrobial resistant pathogens are becoming more common in people, particularly quinolone-resistant *Salmonella* infections. A US study found that reptile and amphibian exposure and food were likely sources of domestically-acquired *Salmonella* infections with plasmid-mediated quinolone resistance during 2008–2014 (Karp et al, 2018). Unpublished data suggest that people have acquired resistant infections from turtles, snakes, and bearded dragons (B. Karp, personal communication, CDC National Antimicrobial Resistance Monitoring System).

Treating reptiles with antimicrobials in an attempt to render them *Salmonella*-free is not effective and has contributed to antimicrobial resistance (Mitchell et al, 2007). Antimicrobial use associated with attempting to raise *Salmonella*-free reptiles may have the unintended consequence of selecting for resistant strains of bacteria, which has led to antimicrobial-resistant infections in people and in turtles where antimicrobials were used on eggs (D'Aoust et al, 1990). Gentamicin resistance was found in *Salmonella*, *E. coli*, and other bacteria isolated from pet turtle farms that attempted to eradicate *Salmonella* using gentamicin (Díaz et al, 2006). Limited data are available regarding the efficacy of other potential methods to suppress or eliminate *Salmonella* bacteria on egg surfaces and hatchlings, such as using a combination of sodium hypochlorite (bleach) and polyhexamethylene biguanide (antiseptic) as an egg wash for red-eared slider turtles (Mitchell et al, 2007). While washing eggs in a detergent solution may reduce *Salmonella* burden in turtle hatchlings (Mitchell et al, 2007), these animals may be subsequently exposed to *Salmonella* through food, water, other animals, and the environment, therefore they may be re-infected at any time (Harris et al, 2010). Multiple outbreaks of *Salmonella* infections in people have been associated with turtles marketed as *Salmonella*-free but that were still shedding the bacteria (Bosch et al, 2016; Walters et al, 2016).

Limited data are available on the prevalence of antimicrobial resistance in wild-caught reptiles and amphibians that were specifically collected for the pet trade. Wild-caught Tokay geckos (*Gekko gecko*) are popular NTPs that are routinely imported to the United States from Indonesia. One study of these imported animals found that commensal (not harmful to the geckos) enteric bacteria (e.g., *Enterobacteriaceae*) in Tokay geckos were resistant to several antimicrobials (Casey et al, 2015). This is a public health concern because pet owners exposed to these animals could acquire antimicrobial-resistant bacteria (Casey et al, 2015). This finding was also a concern because pet owners in the United States often release geckos into the environment, to the point that they have become an invasive species in several states. Thus, there is the potential for released geckos to spread antimicrobial-resistant bacteria to native wildlife and ecosystems (Casey et al, 2015).

Imported reptiles may also contribute to the global spread of antimicrobial resistance. Researchers found that bacterial isolates collected from Asian grass lizards imported to Germany were resistant to multiple classes of antibiotics, including those used to treat salmonellosis and extremely drug-resistant infections in people (Guerra et al, 2010; Unger et al, 2017).

#### Distribution and purchase

People buy reptiles from a variety of sources, including pet shops, other retailers, breeders, trade shows, animal swap meets, beach souvenir shops, flea markets (Gambino-Shirley et al, 2018), street vendors, and online. Despite the 1975 FDA ban on the sale of turtles less than 4 inches (10.16cm) in shell length, these small turtles can still be purchased from numerous sources and are readily available online, so stronger enforcement of the ban is needed (Basler et al, 2014; Gambino-Shirley et al, 2018). People also may find and bring home turtles from the natural environment in the United States.

In general, reptiles are sourced from captive breeding or from the capture of wild animals. With increasing regulation on the import and export of these species, in the 1990's the US reptile industry shifted significantly towards domestic captive breeding (Collis et al, 2011). Many pet turtles in the United States are produced on turtle farms, which grow these animals in large outdoor pools at high densities (Harris et al, 2010). The majority of turtle farms are in Louisiana, which together produce over 50% of the world's pet turtles (Gambino-Shirley et al, 2018).

See Appendix D. Industry layout for more detailed information on distribution and purchase of these animals.

#### Special exposure settings and occupational exposures

Occupational exposures should be considered for workers and volunteers, including:

- commercial and in-home breeding operations;
- pet stores and other retailers selling reptiles;
- distributors;
- transporters;
- veterinary clinics;
- laboratories;
- schools;
- and reptile exhibitions

Some reptiles, particularly small turtles, may be more likely to be sold at transient venues such as flea markets, street vendors, fairs, carnivals, and sporting events, or at non-pet stores such as beach souvenir shops (“Investigation Details,” 2021). The staff at these locations often have limited or no training about reptiles or zoonotic diseases. Recent outbreaks have also identified sales in pet stores resulting in human illness, indicating there may be a concerning trend in increasing small turtle sales in pet stores and not just transient venues (“Investigation Details,” 2021). Reptiles are more likely than other NTPs to be sold online and to require international transport (through imports and exports). Effective outreach, investigation, and enforcement of the small turtle ban require multiagency partnerships (Bosch et al, 2016; Gambino-Shirley et al, 2018; Harris et al, 2010).

#### Industry regulations

See Appendix G. Selected recommendations, standards, guidelines, and regulations for non-traditional pet species for more information on regulations related to reptiles.

### III. Fish, reptiles, amphibians (frogs, toads, salamanders, and newts) and invertebrates that live in aquatic environments

#### Overview

Fish and some amphibians, reptiles, and invertebrates (such as crabs, shrimp, snails, clams, aquatic worms, and corals) require immersion in fresh, brackish, or saltwater for survival or to maintain good health. Providing an optimal environment, including maintaining water temperature and quality, providing appropriate types and amounts of food, and controlling disease are critical to these animals' health. Please refer to the Reptile section for reptile-specific information for the remainder of the Aquatic Environment section.

**Aquatic animals are popular pets**

**Fish are one of the fastest-growing segments of the pet population:**

- A total of 8% of all US households owned fish in 2016, up from 6.5% in 2011 (AVMA Pet Ownership and Demographics Sourcebook, 2017)
- A total of 14.7 million US households have pet fish according to the 2021-2022 APPA National Pet Owners Survey
- The global pet fish industry is valued at over $900 million at wholesale and $3 billion at retail

#### Common risk factors for illness from aquatic animal species

Aquatic animals bathe, excrete, and ingest the water they live in, and these processes circulate and even amplify pathogens found in the water. Anyone can get sick from direct or indirect contact with the animals, water, or habitat substrate in a terrarium or aquarium (“Multistate Outbreak of Human Salmonella Infections,” 2008; Lowry and Smith, 2007; Zarecki et al, 2013). Activities associated with increased risk of zoonotic disease transmission from turtles, frogs, and other aquatic animals include direct and indirect contact with the animal, tank, water, filtration equipment, or other tank contents. Contact with potentially contaminated water has implications for risk of infection from aquatic exhibits (e.g., aquariums and aquatic touch tanks). Ingestion of terrarium or aquarium water can transmit zoonotic pathogens to NTP owners and anyone in contact with the tank. Those who work with aquatic animals are at risk for occupational exposure (Lowry and Smith, 2007).

#### Zoonotic pathogens

##### I. *Salmonella*

Amphibians have been linked to a multistate outbreak and case reports of *Salmonella* infection in the United States. A 2008 multistate outbreak of infections with S*almonella enterica* serotype Typhimurium demonstrated the first known illnesses linked to African dwarf frogs in the United States (Zarecki et al, 2013). This outbreak spanned at least 3 years and caused illnesses predominantly among children (see call out box).

**2008 Multistate outbreak of *Salmonella* Typhimurium linked to african dwarf frogs** (Zareki et. al, 2013)

Exposure to habitat water was the most important risk factor identified among human cases. **Only 27% of infected people had direct contact with a frog, while 60% had contact with aquarium water, demonstrating the risk of indirect contact for *Salmonella* exposure.**

- A total of 376 cases were reported in 44 states including 56 hospitalizations.
- Median patient age was 5 years old, with 51% of patients under 5 years old, and nearly 70% under 10 years old.
- Contact setting and exposure risks for some sick infants included exposure at a **daycare**, cross-contamination of kitchen items including baby bottles, and keeping a frog tank on a changing table.
- The animals were ultimately traced back to a single breeding facility.
- Although there is no regulatory oversight for this industry, the breeder worked cooperatively with public health to control the outbreak. Ongoing biosecurity measures were recommended.

**This outbreak highlights the importance of pediatricians routinely inquiring about pet ownership or potential exposure away from home. Pediatricians should advise families about illness risks associated with NTPs, including that exposure may be indirect, such as through contact with aquarium water containing frogs, and simple hygiene precautions.**

##### II. *Mycobacterium* species—the causative agent of “Fish-handlers disease”

“Fish-handlers disease,” also called “fish tank granuloma,” is caused by infection with environmental *Mycobacterium* bacteria (see box). Mycobacteriosis is a bacterial, systemic granulomatous disease that occurs in aquarium and cultured food fish and can be zoonotic. It can result from infection by several *Mycobacterium* species (Disease Information—Mycobacteriosis, 2020). *Mycobacterium marinum* is one of the most important species found in salt and freshwater. People, especially fish owners, can be exposed through direct contact with animals or their environment, including contaminated water sources, such as aquarium water. Cases of infection with *Mycobacterium marinum* in the United States are uncommon but are likely underreported (Disease Information—Mycobacteriosis, 2020). Progression of infection depends on immune competence of the patient, usually occurring due to injury or non-intact skin (Aubry et al, 2002; Hashish et al, 2018). Rare reports of dissemination such as joint and bone infection have been documented in severely immunocompromised patients (Bhatty et al, 2000; Hashish et al, 2018; Petrini, 2006). Infections have also been reported in reptiles housed in aquaria previously used for fish (Disease Information—Mycobacteriosis, 2020).

**“Fish-handlers disease” or “fish tank granuloma” (caused by *Mycobacterium marinum*)**

Exposure occurs most commonly during cleaning or maintenance of aquariums.

- Breaks in the skin can serve as an entry point.
- Leads to localized skin lesions, usually fingers or hands
- Systemic disease is rare, though more common in high-risk groups.
- Cases are uncommon, but likely underreported nationally.
- Delayed diagnosis may lead to complications.

***Occupational risk:*** Breeders, distributors, or or retailers of aquatic species (such as pet store employees), aquaculture workers, and fish processors, as well as aquatic veterinarians and aquaria workers.

##### III. Other zoonotic diseases and causes of illness or injury to people

Wound infections can be caused by bacteria associated with aquatic animals and their environments. These bacteria may be associated with freshwater (*Aeromonas* spp., *Comamonas* spp., *Edwardsiella ictaluri*, *E. tarda, Burkholderia pseudomallei*); water sources with higher salinity, e.g., brackish or marine water, (*Vibrio* spp.) (Finkelstein and Oren, 2011)^;^ or water sources varying in salinity, e.g., fresh and salt water (*Mycobacterium* spp., *Streptococcus iniae*, *Erysipelothrix rhusiopathiae*) (Lowry and Smith, 2007; Weinstein et al, 2009; Weir et al, 2012b; Yacisin et al, 2017). In 2019, a freshwater home aquarium was recognized as the source of possible transmission of *Burkholderia pseudomallei,* the causative agent of melioidosis, to a person confirmed with the infection who had no history of travel to endemic areas (Dawson et al, 2021).

Bite or puncture injuries can also occur with aquatic species (Abrahamian and Goldstein, 2011). Some aquatic animals can produce venoms or toxins (Abrahamian and Goldstein, 2011; Warwick and Steedman, 2012). Palytoxin is an example of an emerging invertebrate toxin that has been associated with exposure to marine aquariums containing certain species of invertebrates such as *Palythoa* soft corals, *Ostreopsis* dinoflagellates, and *Trichodesmium* cyanobacteria. Palytoxin is unique in that exposure can occur through inhalation, ocular or skin contact, or ingestion and has caused human illnesses (Pelin et al, 2016). Palytoxin exposures commonly occur while cleaning aquaria or intentionally removing zoanthid corals to control populations from overrunning an aquarium.

Ingestion of bacteria including *Vibrio* spp., *E. tarda*, *Lactococcus garvieae*, and *Plesiomonas shigelloides* in untreated water from aquatic animal habitats can also cause illness (Lowry and Smith, 2007; Weir et al, 2012b).

#### Antimicrobial use, stewardship, and resistant pathogens in aquatic animal species

It is important to practice antimicrobial stewardship principals when treating aquatic animals to prevent the development of antimicrobial-resistant pathogens (Broens and van Geijlswijk, 2018; OE et al, 2009; Weir et al, 2012b). Ornamental fish have been associated with the spread of antimicrobial-resistant bacterial pathogens, as well as resistance mechanisms (Weir et al, 2012a). A 2011 review of zoonotic bacterial pathogens that caused infections in people found that most reported sporadic human infections were associated with *Mycobacterium marinum* and *Salmonella enterica* serotype Paratyphi B var. L (+) tartrate+ formerly known as the variant Java (Weir et al, 2012a). This review also reported high levels of resistance to amoxicillin, penicillin, tetracycline, and oxytetracycline in *Aeromonas*, *Mycobacterium*, and *Salmonella* bacteria in ornamental fish, a concern given the large size and global reach of this industry.

Reports of people self-medicating with antimicrobials intended for aquatic use have prompted FDA to formally advise against this practice (Ornamental Fish Drugs and You, 2021; Zhang et al, 2020). To practice and promote antimicrobial stewardship, some pet industry retailers have elected to not sell antimicrobials for aquatic life and other species or to sell them in forms that are not easily consumed by people (Wei-Haas, 2017).

The World Organisation for Animal Health (OIE) Aquatic Animal Health Code provides recommendations for responsible use of antimicrobials in aquatic animals (Antimicrobial Resistance, 2021). FDA CVM has approved several antimicrobial drugs that veterinarians in the United States can prescribe for aquatic animals (Approved Aquaculture Drugs, 2022). FDA CVM, however, recognizes the relative few antimicrobial drugs available for aquatic species and provides flexibility for use of Veterinary Feed Directive drugs in medicated feed for minor species, including all aquatic species (“FDA Releases Draft Guidance,” 2021; Approved Aquaculture Drugs, 2022).

#### Distribution and purchase

The distribution pathways of ornamental fish and invertebrates are complex (Rising Tide Conservation—Saving the Ocean One Fry at a Time, n.d.; “Wild Caught Ornamental Fish,” 2021). Fish shipments move thousands of miles from production sources and countries of origin via holding and transshipment facilities, wholesalers, and retailers. Ornamental fish and invertebrate farms are traditionally small, family-run operations with limited veterinary medical oversight or regulation, but they can vary in size (Weir et al, 2012b). Several large aquatic animal breeding facilities exist in the United States, many of which employ biologists and veterinarians to maintain the health of their animals. These facilities import large quantities of animals from tropical and subtropical regions with Asia being the largest regional exporter (Overview of Global Imports, n.d.). Marine fish and invertebrates are more likely to be wild caught outside the United States and imported, as opposed to freshwater aquatic animals and species. Conservation efforts are underway to develop and promote aquaculture of marine ornamental fish through the collaborative efforts of researchers, public aquaria, hobbyists, and conservation groups (Rising Tide Conservation—Saving the Ocean One Fry at a Time, n.d.). Aquaculture provides a sustainable alternative to wild fish collection, which can stress wild fish populations and damage the coral reefs where they live, increasing the number of marine ornamental fish species available to public aquaria and commercially to hobbyists.

Amphibians such as dart frogs, African dwarf frogs, pac-man frogs, and tree frogs are more commonly captive bred, but others such as the fire-bellied toad and most other toads are wild-harvested. All these imported animals (wild and captive-bred) arrive at distribution facilities that might also be breeders, and are then sold to the public mainly in retail stores or through online sales via ground or air shipping (Appendix D. Industry layout).

For each of these animal distribution pathways, the wholesaler or distributor is a critical link between the collector or producer and the customer. Wholesalers receive ornamental aquatic species from a variety of often-distant sources. The aquatic animals must then be acclimatized to a holding facility, and in some cases trained to eat artificial diets over a period ranging from a few days to a few weeks (“Aquatic Wholesaler Best Management Practices,” 2016).

#### Special exposure settings and occupational exposures

Aquatic exhibits such as those with aquariums and aquatic touch tanks may be associated with increased risk for zoonotic disease transmission. See the NASPHV Compendium of Measures to Prevent Disease Associated with Animals in Public Settings for more information.

Occupational exposures to *Mycobacterium* (which causes fish handlers disease) may occur during cleaning and caring for aquatic species. An outbreak of *Mycobacterium marinum* infections among workers involved contact with fish from a fish market in New York City from 2013–2014 (Yacisin et al, 2017; Sia et al, 2016). Those at risk for occupational exposure include breeders, distributors, or retailers of aquatic species (such as pet store employees), aquaculture workers, fish processors, aquarium and aquatic animal exhibit workers. Fish and other aquatic animals are also commonly found in schools, nursing homes, and daycare settings where staff may have limited or variable training on animal husbandry and zoonotic diseases.

#### Industry regulations

See Appendix G. Selected recommendations, standards, guidelines, and regulations for non-traditional pet species for more information on regulations related to NTPs.

### IV. Backyard poultry

#### Overview

Backyard poultry most commonly includes domestic land fowl (e.g., chickens, turkeys), domestic waterfowl (e.g., ducks, geese), and game birds (e.g., wild turkeys, wild geese, pheasants). People also keep other poultry species such as guinea fowl or swans in backyards. For all backyard poultry, the different life stages (e.g., hatching egg, chick, chicken) present a risk of disease transmission to people, particularly infections with *Salmonella* and *Campylobacter*.

**Backyard poultry are increasingly popular**

A 2014 study estimated that 50 million live poultry are sold annually, generating $50–$70 million in sales.

A total of 1.1% of all US households owned backyard poultry in 2016.

- A total of 15.5 million birds
- This represents a 200% increase since 2006

A 2013 USDA study on urban chicken ownership in 4 major US cities (Denver, Los Angeles, Miami, and New York City) found:

- Of people surveyed, 0.8% owned chickens and 4% of those without were planning to have them within the next 5 years

Membership is increasing among online backyard poultry community forums.

- One forum grew to 436,000 members in 2016 from 50,000 members in 2010

Owning backyard poultry is increasing in popularity (AVMA Pet Ownership and Demographics Sourcebook, 2017; Elkhoraibi et al, 2014; Thompson, 2016), especially backyard chicken flocks in urban and suburban areas. During the COVID-19 pandemic, agricultural stores and media outlets reported record purchasing of poultry during 2020 for egg and meat consumption and to have as pets (Nichols et al, 2021). People keep backyard flocks for many reasons, including as a food source (such as from fresh eggs or meat), companionship, exhibition, hobby, and educational purposes (AVMA Pet Ownership and Demographics Sourcebook, 2017; Backyard Poultry, 2022; Elkhoraibi et al, 2014; “Frost on Chickens,” n.d.; Thompson, 2016). Despite public health recommendations to not have poultry in settings with children younger than 5 years old and adults 65 and older, poultry are sometimes found in daycare or nursing home settings as resident pets. They may also be used in classroom projects (e.g., incubating and hatching eggs to demonstrate the chicken's life cycle).

#### Common risk factors for illness from backyard poultry

The shift in importance of backyard flocks with respect to care and concern for individual birds has led to behaviors that increase the risk of disease transmission from the birds and their environment to people, such as keeping poultry inside the home and kissing or snuggling birds (Basler et al, 2016). Some practices that can increase risk of disease transmission by contaminating the indoor or home environment include allowing backyard poultry to roam freely indoors, cleaning poultry supplies inside the home, wearing the same shoes or clothing while working with backyard poultry or their coop, and improperly or not washing hands after handling the birds or potentially contaminated supplies.

Efforts are underway to use an integrated One Health approach with collaboration between government, including public health and animal health officials, and industry partners to reduce pathogens in poultry and help prevent disease transmission to people. Implementation of prevention measures, including education on risk and reducing exposure, should occur along the backyard poultry supply chain, including the mail-order hatchery industry, feed stores, online retailers, shows, and exhibits, and should include agriculture extension agents, veterinarians, healthcare providers, and backyard flock owners (Anderson et al, 2016; Basler et al, 2014; Behravesh et al, 2014; Gaffga et al, 2012; Loharikar et al, 2013; Loharikar et al, 2012).

#### Zoonotic pathogens

Healthy poultry can carry *Salmonella* and *Campylobacter* bacteria and spread them to people, resulting in illness. Highly pathogenic avian influenza and virulent Newcastle disease may cause serious illness in poultry, and both diseases have established national disease monitoring programs with eradication efforts implemented when birds or flocks test positive.

##### I. *Salmonella*

Despite awareness of the risk of salmonellosis from handling raw poultry, some people are not aware that *Salmonella* can also spread between live poultry and people, and education is needed (Basler et al, 2016, 2014). Poultry are a known source of *Salmonella* infection in people, and transmission to people after contact with backyard poultry or their environment is a well-recognized public health problem (Basler et al, 2016; Gaffga et al, 2012; Loharikar et al, 2013; Loharikar et al, 2012). Baby poultry, particularly chicks and ducklings, have been linked to several recent and extensive outbreaks. Poultry can be infected with *Salmonella* through multiple routes, including by contact with infected birds, vertical transmission from infected hens to their eggs, or from contaminated feed (Anderson et al, 2016; Basler et al, 2014; Behravesh et al, 2014; Gaffga et al, 2012; Loharikar et al, 2013; Loharikar et al, 2012).

***Salmonella* infections associated with backyard poultry**

Poultry commonly carry *Salmonella* as normal flora in their intestinal tract. Poultry may appear healthy while infected, but some *Salmonella* strains can cause illness in poultry. The bacteria are shed in their droppings and can contaminate any surface where the birds live and roam, as well as their feathers, feet, and body (Basler et al, 2016).

A total of 105 reported outbreaks of *Salmonella* outbreaks were identified in the literature review from 1996–2017 associated with contact with backyard poultry, the majority of these occurring since 2007.

During 2020, 1,722 outbreak-associated *Salmonella* illnesses were linked to contact with privately-owned poultry resulting from infections with 12 *Salmonella* serotypes. This was the highest number of backyard poultry-associated outbreaks and outbreak-associated illnesses in a 1-year period on record for the United States (Nichols et al, 2021).

Visit Outbreak of Salmonella Infections Linked to Backyard Poultry | CDC for up-to-date outbreak information

***Occupational risk:*** Agricultural retail store employees and postal workers have been infected during outbreaks (Having et al, 2015; Sidge, 2019)

As the popularity of backyard flock ownership has increased, the number of reported outbreaks of infections with *Salmonella* in people from contact with backyard poultry has also dramatically increased. Human infections result from contact with infected poultry and their environment, including enclosures in which they are kept or transported. A recent study by the Michigan Department of Health and Human Services found that 26% of poultry shipping boxes over a 3-year period were contaminated with *Salmonella*, including four subtypes known to be previously associated with human illness (Habing et al, 2015; Sidge, 2019).

Outbreaks affecting adults and children have increased, with reported cases typically beginning in January and continuing through much of the year (Behravesh et al, 2014). During 2020, data reported to PulseNet, the national laboratory network for enteric disease surveillance in the United States, indicated the highest number of backyard poultry-associated outbreaks with *Salmonella* infections and outbreak-associated illnesses in a 1-year period on record for the United States. It is estimated that because only a small proportion of *Salmonella* infections are diagnosed and reported to public health departments, there are an additional unreported 29 infections for every reported case (Hale et al, 2012; Scallan et al, 2011).

The backyard poultry linked to previous outbreaks traced back to several sources including multiple hatcheries, agricultural feed stores, and internet sites. Facilities with people at higher risk for serious illness from *Salmonella* infection have experienced outbreaks, including school classrooms, daycare centers, and nursing homes.

**Notable settings for outbreaks of infections with *Salmonella* associated with backyard poultry** (Behravesh et al, 2014; Gaffga et al, 2012; Loharikar et al, 2013; Loharikar et al, 2012)

*Salmonella* outbreaks associated with backyard poultry have occurred in a variety of settings. Commonly, these include **homes** where chicks have been allowed in to roam freely or kept in bathrooms, or when there may be cross-contamination of kitchen or dining areas.

In one outbreak, an unlicensed caterer kept baby poultry in the kitchen leading to cross contamination and three foodborne outbreaks.

Outbreaks associated with poultry visiting daycares, nursing homes, and other public settings have also occurred.

**These outbreaks support the recommendation that poultry should not visit or be kept in facilities with people in high-risk groups or where food or beverages for people are prepared or consumed due to risk of cross-contamination.**

##### II. *Campylobacter*

Poultry often carry *Campylobacter* bacteria in their intestinal tract and can appear healthy. In the United States, most illnesses in people occur from eating raw or undercooked poultry or coming in contact with surfaces contaminated by raw or undercooked poultry (Campylobacter and Pets, 2021). Other risk factors for contracting *Campylobacter* infection from poultry-related sources include keeping backyard poultry, butchering poultry for meat, and contact with contaminated eggs (Campylobacter and Pets, 2021). Occupational exposures and resulting infections have been documented in abattoir workers and people working in poultry processing facilities (de Perio et al, 2013).

*Campylobacter* infection in people tends to occur sporadically, with outbreaks rarely reported. A summary of 934 sporadic *Campylobacter* infections in Minnesota residents during 2012–2016 who reported an animal agriculture exposure before their illness revealed that 54% had contact with poultry or their environment (Minnesota Department of health, unpublished data; Klumb et al, 2020). *Campylobacter* infection in people are likely to be underdiagnosed, in part due to changes in laboratory testing (e.g., increase in culture-independent diagnostic testing [CIDT]) (Marder et al, 2017) and difficulty growing and isolating the organism. *Campylobacter* infection in people may also be underreported because it is not a reportable condition in many jurisdictions.

##### III. Avian influenza

Avian influenza refers to infection of birds with influenza type A viruses whose natural hosts include many species of wild waterfowl and domestic water and land birds (Influenza in Animals, 2018). Depending on the pathogenicity and virulence of the virus, waterfowl, such as ducks, geese, shorebirds, and swans, do not always show signs of avian influenza virus (AIV) infection, but poultry flocks can experience a range of illness, from decreased egg production to extremely high death rates. Avian influenza viruses are classified as low pathogenic or highly pathogenic based on the ability of the virus to cause disease in chickens in the laboratory and/or on certain genetic markers of pathogenicity. During 2014–2015 more than 50 million chickens and turkeys died or were destroyed in the most devastating outbreak of infections with highly pathogenic avian influenza A (HPAI) viruses in domestic poultry in the United States to date.

**Outbreaks of infections with avian influenza virus in domestic poultry flocks**

Both low pathogenic avian influenza A (LPAI) and highly pathogenic avian influenza A (HPAI) viruses have been reported in recent years in the United States and around the world.

**2014–2015 Outbreaks of infections with highly pathogenic avian influenza viruses in the United States** (Final Report for the 2014–2015 Outbreak of Highly Pathogenic Avian Influenza (HPAI) in the United States, 2016)

- Most severe foreign animal disease outbreak in US history
- Affected 211 commercial and 21 backyard flocks
- A total of 50 million birds lost or eliminated to stop disease spread
- Almost $1 billion in federal expenditures for depopulation, cleaning, and disinfection of farms, and indemnities for lost birds
- **No human cases of infection with HPAI reported from this outbreak**

AIV transmission from birds to people can occur through direct contact with infected birds, indirect contact with virus-contaminated surfaces, or through inhalation of virus in the air in droplets or dust (Avian Influenza A Virus Infections in Humans, 2022; “How Infected Backyard Poultry Could Spread Bird Flu,” n.d.). In cases of human infection, the spread from one sick person to another is very rare and not sustained. To date, there have been no reports of human illnesses caused by AIV in the United States associated with infected birds (Avian Influenza A Virus Infections in Humans, 2022). One reported AIV infection in the United States was from exposure to AIV-infected cats (Influenza in Cats, 2018; Lee et al, 2017). Cases of illness and death in people in Africa, Asia, and Europe have occurred from several AIV subtypes (mainly H5, H7, and H9), and with rare, isolated reports of limited, non-sustained human-to-human transmission (Liu et al, 2013). Most initial cases of human infection occur after unprotected contact with sick birds (including in live animal market settings) or virus-contaminated surfaces. Because of the possibility that avian influenza viruses could change (primarily by reassortment or mutation) and gain the ability to spread easily between people, monitoring wild birds and domestic poultry for outbreaks and monitoring for human infections and person-to-person spread is extremely important. People who have contact with known or possibly infected birds should take precautions to protect against infection and monitor their health for possible symptoms (Avian Influenza A Virus Infections in Humans, 2022).

##### IV. Avian paramyxovirus (the cause of Virulent Newcastle Disease)

Newcastle disease is a viral disease of birds that can cause a range of illness in birds, from no symptoms to mild illness or up to 100% mortality, depending on the viral strain. Newcastle disease viruses rarely infect people; when they do, they can cause mild to severe conjunctivitis or mild influenza-like symptoms. Reported human cases have occurred only after exposure to particularly high concentrations of virus (Virulent Newcastle Disease (vND), 2021). Virulent Newcastle disease (vND) refers to the most severe form of the disease in poultry, which is only caused by certain viral strains. Although many birds can become infected, wild cormorants and domestic poultry are particularly susceptible. Large outbreaks of vND occur periodically in commercial poultry, and previous outbreaks in the western United States have resulted in large losses of birds. This outbreak is primarily associated with backyard exhibition chickens raised or smuggled illegally for fighting. Hundreds of premises have been affected, including homes with backyard flocks in other states, due to movement of exposed and infected birds (Virulent Newcastle Disease (vND), 2021).

#### Distribution and purchase

The poultry industry includes operations that range from commercial production systems consisting of hundreds of thousands or even millions of birds, to backyard flocks with just a few birds. Birds from each of these systems may encounter each other at certain points in distribution supply chains that can increase the risk of transmitting pathogens, particularly *Salmonella*.

People can acquire poultry of all ages through different venues, including feed stores and other retail locations, poultry hatchery companies, poultry exhibitions at state and county fairs, print or online advertisement by corporate feed stores or private individuals, auction markets, flea markets, private bird swaps, and websites.

Approximately 20 mail-order hatcheries provide the majority of live poultry sold in the United States, and these core hatcheries often supply hatchlings to other resale hatcheries that do not actually hatch poultry (Loharikar et al, 2012; Nichols et al, 2018). Hatchlings are distributed nationally to retail stores, through the US Postal Service for pick-up by owners at their local post office, or directly to homes. Within 24 hours after hatching, baby poultry are shipped in cardboard boxes containing up to 100 birds through the US Postal Service, provided the birds can be delivered within 72 hours of hatching. Data collected from recent outbreaks of *Salmonella* infections linked to backyard poultry indicate that sick people mostly purchased baby poultry through agricultural retail stores, and the agricultural retail stores usually source the birds from a variety of mail-order hatcheries around the United States (Habing et al, 2015; “Hatcheries work with CDC,” 2018; Nichols et al, 2018). Mail-order hatcheries may also source hatching eggs or hatchlings from commercial egg suppliers (Nichols et al, 2018).

The sale of live poultry occurs year-round but peaks during spring/summer months. Typically, outbreaks are seen one to two months after the sales peak. One agricultural retail store has adopted the practice of setting a minimum number of chicks and ducks to be purchased to discourage people from getting them as pets.

Hatcheries may engage in various distribution practices that can increase the risk of cross-contamination of pathogens and decrease the traceability of birds, such as “drop-shipping,” “multiplying,” and “trans-shipping” (Appendix C. Glossary) (Nichols et al, 2018). Feed or other retail stores may purchase birds from one source hatchery but unknowingly receive birds from multiple mail-order hatcheries who drop-ship, trans-ship, or use multiplier birds from outside sources (Nichols et al, 2018). Multiplying and trans-shipping practices can introduce *Salmonella* from other source flocks into the hatchery. Drop-shipping and other similar practices that mix birds from different hatcheries make it difficult to identify the source of the birds in the case of an outbreak (Nichols et al, 2018). Traceback investigation to identify the original source of *Salmonella* in an outbreak is critical for implementing measures to stop the continued spread of *Salmonella* and prevent additional human illness as well as future similar outbreaks from occurring (Nichols et al, 2018; Robertson et al, 2019).

#### Special exposure settings and occupational exposures

People involved in breeding, raising, and transporting backyard poultry, including agricultural retail store staff and volunteers, agricultural/farm staff, and volunteers, petting farm/zoo staff and volunteers, postal and shipping workers, law enforcement, animal control officers, and veterinarians, are at risk of occupational exposure to pathogens. In the case of an outbreak of Newcastle disease or avian influenza in poultry, workers at risk include outbreak responders who may be state or federal veterinarians, animal health technicians or other staff persons, farmers and flock owners, contractors hired for cleaning and disinfection or animal disposal, and others (Information for Specific Groups, 2022). Other people at risk who come in contact with poultry at their place of work or who are involved in cleaning habitats or coops can include teachers, daycare or other childcare providers, camp counselors and volunteers, nursing home or assisted living staff, and photographers. Employers are responsible for providing training and preventive strategies to employees of these locations, as well as students, visitors, parents, or residents, who may not be aware of the disease risks associated with backyard poultry. See the Recommendations section for more information on Safe Handling.

#### Industry regulations

See Appendix G. Selected recommendations, standards, guidelines, and regulations for non-traditional pet species for more information on regulations related to backyard poultry.

### V. Feeder animals

#### Overview

Feeder animals, whether live or frozen, include NTP species such as rats, mice, rabbits, or chicks that are fed to other animals such as reptiles, birds of prey, aquatic animals, zoo animals, and other carnivores. Typical feeder species are covered in the Rodents and other small mammals section and the Backyard poultry section, but feeder animals have unique production and distribution characteristics that warrant a separate section to highlight some of the risks associated with their production and use.

According to the American Pet Products Association (APPA) 2019-2020 National Pet Owners Survey, 63% of snake owners reported using live and frozen feeder rodents and 31% of lizard owners reported buying “live food” other than worms and insects (e.g., roaches, crickets) for their lizards “(Pet Industry Market Size,” 2021).

Like all NTP species, feeder animals can carry zoonotic pathogens while appearing healthy. Feeder animals have been implicated in multiple multistate disease outbreaks, including outbreaks of infections with *Salmonella,* with at least one international outbreak involving multidrug-resistant strains of *Salmonella* (Basler et al, 2014; Cartwright et al, 2016; Goupil et al, 2012; “Notes from the Field: Infections with Salmonella,” 2012; Pet Food Safety, 2022; “Salmonella Typhimurium Infections,” 2014). They may also carry pathogens like LCMV, *Streptobacillus moniliformis* (the cause of rat bite fever), and others that pose zoonotic disease risks.

#### Common risk factors for illness from feeder animals

NTP owners who use feeder animals may not be aware of the risks they pose and may not follow disease prevention principles at home such as appropriate handwashing, having a separate storage area away from human food storage, and other procedures.

Feeder animals are unique in that they are sold live or dead, fresh or frozen. People should feed fresh or frozen dead feeder animals to pets to reduce the risk of injury to the pet as well as the person feeding the pet from a live animal (Pet Food Safety, 2022). To reduce the risk of bites from reptiles, people should be careful not to handle these NTPs after handling any feeder animals and to wash hands thoroughly.

The fur and skin of frozen or live feeder animals may get contaminated with bacteria like *Salmonella* during production and euthanasia when they urinate and defecate (Pet Food Safety, 2022). Feeder animals are not cleaned before they are sold because reptiles will refuse to eat them. This increases the risk of contamination when pet owners handle, store, or thaw these animals. Contamination of refrigerators, freezers, kitchen utensils, countertops, and microwave ovens have been associated with human *Salmonella* infections (“Salmonella, Feeder Rodents, and Pet Reptiles,” 2020). Some, but not all, frozen rodent companies may use irradiation to reduce external contamination (“Salmonella, Feeder Rodents, and Pet Reptiles,” 2020).

Frozen feeder rodents can contaminate human food if they are stored together and can contaminate surfaces where they are thawed or anything they touch. People can get sick from touching contaminated surfaces even if they do not handle the animal directly. In outbreaks related to feeder animals, common risk factors include lack of handwashing after handling feeder animals or feeding reptiles, and cross-contamination of surfaces and utensils used in feeding the animals with those used by people in the household.

#### Zoonotic pathogens

##### I. *Salmonella*

Live and frozen feeder animals have been associated with multiple outbreaks of *Salmonella* infection in the United States. Rodents used as feeder animals were most commonly associated with these outbreaks (“Notes from the Field: Infections with Salmonella,” 2012; “Salmonella Typhimurium Infections,” 2014). In addition to feeder rodents, feeder animals like chicks and other poultry pose a risk of *Salmonella* infection to those caring for NTPs. Freezing does not kill *Salmonella*, and the bacteria have been shown to survive freezing for over 19 months (Cartwright et al, 2016). For more detailed information on *Salmonella* infections see the Rodents and other small mammals and Backyard poultry sections.

Seven outbreaks of *Salmonella* infections associated with frozen feeder rodents were identified in the results of the literature review and NORS data request, including

- A 2010 multistate outbreak that resulted in 34 illnesses in 17 states (“Notes from the Field: Infections with Salmonella,” 2012)
- A 2014 multistate outbreak that resulted in 41 illnesses in 21 states (“Salmonella Typhimurium Infections,” 2014)

Exposure to frozen feeder rodents used as food for pet reptiles was associated with human illness in a 2009 international outbreak of tetracycline-resistant *Salmonella* infections. Only 13% of people sickened in the outbreak investigation were aware of the association between *Salmonella* infection and rats or mice, evidence that there is a need for increased consumer awareness of the risk (Cartwright et al, 2016).

Thorough records and clearly labeled packages are also needed from distributors and producers to allow for efficient identification of potentially contaminated animal products.

In a 2010 outbreak of *Salmonella* infections, illnesses were associated with exposure to pet reptiles and frozen feeder rodents used as feed for pet reptiles (Cartwright et al, 2016; “Salmonella Typhimurium Infections,” 2014) This multistate outbreak was linked to an international outbreak of *Salmonella* in the United Kingdom in 2009, in which feeder mice imported from the United States were found to be the source of human illness.

##### II. Lymphocytic choriomeningitis virus (LCMV)

Live feeder rodents have been associated with human cases of infection with LCMV in the United States, mainly among employees at feeder rodent breeding facilities (Knust et al, 2014). The principal transmission route of LCMV to people is through contact with the urine, feces, saliva, or blood of the natural reservoir species, the house mouse (*M. musculus*) (Knust et al, 2014). For more detailed information on LCMV infections associated with small mammals see Rodents and other small mammals.

Exposure to wild mice was believed to the source of LCMV introduction into a breeding colony that led to a 2012 multistate outbreak linked to a rodent breeding facility, resulting in 4 confirmed cases of aseptic meningitis in employees (Knust et al, 2014). This outbreak illustrates potential occupational health risks related to feeder rodents and the need to educate purchasers and handlers throughout the supply chain. Investigators found that nearly one-third of rodent facility employees tested positive for LCMV antibodies. At each of the two facilities where testing was performed for frozen feeder mice, 21% and 33% of the mice had antibodies to LCMV. To stop transmission of the virus, the facility quarantined shipments, depopulated all live mice, and disposed of frozen mice (Knust et al, 2014).

##### III. *Streptobacillus moniliformis* (the cause of rat-bite fever)

Rat-bite fever is a rare bacterial disease caused by *Streptobacillus moniliformis* that is primarily associated with exposure to colonized rats. Other rodents that can carry the bacteria are mice, guinea pigs, gerbils, and squirrels (“Infection in Animals, Rat-Bite Fever,” 2019). Rat-bite fever is not commonly associated with feeder animals, but it is important to note that there is a risk for rat-bite fever when working with live feeder rodents. Colonized rats usually appear healthy, so symptoms consistent with rat-bite fever that occur in people breeding, distributing, selling, or using rats as feeder rodents should be evaluated and treated properly (Hardin, 2013).

When infected with the bacterium that causes **rat-bite fever**, rodents usually appear healthy. When people who work around or handle rodents develop symptoms consistent with rat bite fever they should seek medical care and let their provider know they have contact with rodents (Hardin, 2013; “Infection in Animals, Rat-Bite Fever,” 2019).

Increased education and awareness of rat-bite fever risks are needed among feeder rodent producers, people caring for rodents, and healthcare providers.

For more detailed information on rat bite fever infections associated with small mammals see Rodents and other small mammals.

#### Distribution and purchase

With increasing popularity and growth of the pet reptile and amphibian industry, the demand for feeder animals has also increased. The feeder animal supply is diverse in production and distribution, but large-scale commercial operations raise the majority of feeder animals (Hardin, 2013). Some large breeding facilities produce thousands of animals weekly. In general, the industry is vertically integrated (multiple stages of production operated by a single company) with commercial breeders supplying affiliated distributors. Most rodents move from large-scale production facilities through distributors to the retail market (Appendix D. Industry layout). Vendors buy and sell these animals based on price and availability.

Often, pet rodents and feeder rodents are supplied by both small- and large-scale production facilities from the same location, and in most national retail stores, these animals live in the same habitat. Some feeder animal operations also sell animals online. Internet commerce makes the purchase of feeder animals more convenient for consumers but may increase the opportunity for multistate zoonotic disease outbreaks (Fuller et al, 2008).

#### Special exposure and occupational health considerations

People involved in breeding, raising, and transporting live or frozen feeder animals are at risk for occupational exposure to disease, including employees of production facilities and pet store employees. Other people who come in contact with live or frozen feeder animals such as consumers who feed these to non-traditional pets may not be aware of the disease risks associated with feeder animals, thus placing themselves at higher risk by not taking preventive measures.

#### Industry regulations

See Appendix G. Selected recommendations, standards, guidelines, and regulations for non-traditional pet species for more information on regulations related to non-traditional pets.

## Recommendations

### I. Overview

Given the number and size of outbreaks associated with NTPs, the information that follows provides One Health partners, government agencies representing public health and animal health officials, NTP industry including breeders, distributors, and retailers (including pet, agricultural, and other retailers where animals are sold), NTP industry groups (professional associations and affiliated non-governmental organizations), human healthcare and veterinary care providers (including veterinarians, physicians, and allied health professionals), and others associated with managing NTP species in public settings resources to prevent zoonotic disease transmission and spread. The recommendations that follow in this Compendium aim to prevent infections in people as well as maintain animal health and welfare. Compliance with these recommendations will benefit all partners through disease prevention and control, which can improve human and animal health, animal welfare, and provide economic benefits. As new information emerges about the transmission, epidemiology, and pathogenesis of specific zoonotic pathogens, this Compendium will be revised and updated to reflect the most current recommendations.

The recommendations in this Compendium should be incorporated into best practices and protocols by One Health partners including

- Government agencies representing public health and animal health officials;
- NTP breeders, distributors, and retailers including pet and agricultural industry employers;
- NTP industry groups, professional associations, and affiliated non-governmental organizations;
- Human healthcare and veterinary care providers; and
- Others associated with managing NTP species in public settings such as schools, zoos, and aquaria.

The following section includes generalized recommendations that apply across all sectors as well as specific recommendations organized by category and developed for each sector where applicable. The recommendations can be tailored to specific settings and incorporated into best practices and protocols. The recommendations apply to all covered NTP species unless otherwise noted. One Health partners and the organizations representing these partners, such as industry groups and professional associations, are encouraged to disseminate this Compendium widely. Reference the Background section of this document for evidence to support these recommendations.

Certain federal agencies and organizations in the United States have standards, recommendations, and guidelines for reducing health risks associated with contact with NTP species. This Compendium uses those existing recommendations as well as information, publications, reports, and guidance documents from multiple organizations, and existing laws and regulations in the United States. Where gaps existed, the committee members and consultant subject matter experts used these and other references to achieve consensus on best recommendations based on available evidence.

### II. General recommendations for all partners

#### A. Awareness and education

***Suggested messaging to share with pet owners and the general public***

A One Health approach to public awareness and education is important because of the wide range of settings in which people interact with NTPs, as well as the large variation in the scale and movement of NTPs through supply chains (Appendix D. Industry layout). All One Health partners involved in the NTP life cycle play an important role in informing animal owners of zoonotic disease risks and the many ways zoonotic diseases can spread from pets and other animals to people (Pickering et al, 2008). Sharing consistent messages across all One Health partners, from industry to animal health and human health organizations, will ultimately help consumers understand the benefits and risks of animal interaction and pet ownership, pick an animal that fits their health status and lifestyle, and safely enjoy interacting with animals in various settings. The following sample messaging can be used by all partners and shared with NTP owners and the general public.

**Consistent messages across all One Health partners will help consumers**

1. Understand the benefits and risks of animal interaction and pet ownership;
2. Pick an animal that fits their health status and lifestyle; and
3. Safely enjoy their pets.

##### Audiences for messaging

Awareness and education efforts should be tailored to anyone who owns or may come in to contact with NTPs or their environments. People might come in contact with NTPs at home or away from home in places such as pet stores, agricultural feed stores, fairs, petting zoos or farms, schools, daycares, camps, long-term care facilities, educational shows, flea markets, animal swap meets, work, and more. Messaging about the risk for more severe illness from NTPs should be tailored to parents/caretakers of children younger than 5 years old and other people at higher risk of illness including adults 65 and older, people with weakened immune systems due to illness or certain medications, and pregnant people.

All relevant partners should work together to communicate to the public and NTP owners, including those who are fostering or adopting NTPs, the following information:

**Veterinarians**

- Discuss responsible pet ownership, choosing an appropriate pet, animal husbandry, and preventive care with current and prospective pet owners.
- Direct staff with occupational risks to relevant resources related to zoonotic disease prevention.
- Provide recommendations on animal husbandry and welfare, biosecurity, disease prevention including zoonoses, and appropriate disease treatment in the case of sick animals or animals suspected to be infected with a zoonotic pathogen. This can include sharing trusted resources like CDC's Healthy Pets, Healthy People website.

**Physicians and allied healthcare providers**

- Routinely ask patients if they interact with animals at home or away from home and what type of animals they come in contact with.
- Discuss disease prevention precautions such as proper handwashing with patients who are NTP owners or at occupational risk of exposure.
- Advise people at higher risk of illness which types of animals to avoid.
- Share trusted resources like CDC's Healthy Pets, Healthy People website to provide patients with more detailed information on protecting their health around animals.

**Government agencies representing public health and animal health officials**

Provide resources and community outreach to inform and educate constituents on relevant local, state, and federal laws related to NTP ownership, zoonotic disease risks associated with NTPs, and evidence-based zoonotic disease prevention methods.

- Share trusted resources with constituents, including CDC's Healthy Pets, Healthy People website, educational resources (Appendix H), and other references provided in this Compendium to provide more detailed information on protecting health.

**Industry leadership** (breeders, distributors/importers, retailers)

- Educate animal owners at each level of the NTP supply chain at which a consumer may be selecting, ordering, purchasing, or adopting an NTP, including online marketing and sales.
- Educate all employees about infection prevention for themselves and customers.
- Educate all employees on how to communicate zoonotic disease prevention information to current and potential pet owners. A retail pet store employee is often the first person an NTP owner may interact with to learn about these pets, thus employee engagement in this process is critical.

**Other relevant One Health partners** (industry groups, associations, and affiliated organizations)

- Participate in efforts to raise awareness among communities and partners.
- Include zoonotic disease information in existing or new clinical briefs, guidance documents, and position statements.

##### General messaging

Include the following types of public awareness messaging:

- Benefits of human-animal interactions and the human-animal bond.
- Animals can sometimes carry harmful germs even when they appear healthy; these germs can make people sick, especially those people at higher risk of illness.
- People can get sick after contact with harmful germs from animals, their habitats, environments where animals live and roam (e.g., terrarium, aquaria, bedding), and supplies (e.g., bowls, habitat accessories, leashes and collars).
- When selecting an appropriate pet for the household, a pet owner should consider
  - animal temperament, behavior, lifespan, husbandry, habitat, diet and exercise needs, cost, and veterinary care needs (How to Stay Healthy Around Pets, 2022);
  - animal source (captive or wild-collected);
  - principles of responsible NTP ownership; and
  - whether the household includes people at higher risk of illness.
- Specific messaging for people at higher risk of infection. Messages should be consistent with CDC's recommendation that people at higher risk for serious illness avoid direct contact with certain animals.
- Simple steps to enjoy NTPs and other animals while minimizing the risks of zoonotic disease transmission.
  - Steps for engaging in safe animal contact and ways to practice proper personal hygiene (e.g., washing hands) and pet hygiene (e.g., cleaning up after pets).

Education resources

**Deliver messages *in multiple formats* (e,g,, posted signage, pamphlets, stickers, and verbal reminders) and *in multiple languages* when relevant to reach the widest possible audience.**

Detailed messages and educational materials are available on CDC's Healthy Pets, Healthy People website as well as printable resources found here:

Print Materials | Healthy Pets, Healthy People | CDC

For public venues, the *Compendium of Measures to Prevent Disease Associated with Animals in Public Settings* contains thorough and detailed recommendations.

More educational resources are found in Appendix H.

Information for current and potential NTP owners should be incorporated into existing communication and education methods. These might include client or patient intake forms, investigative interviews, and other educational outreach. It is especially important that people at higher risk for serious illness from zoonotic diseases and their caregivers are aware of the risks and receive information about preventing zoonotic disease transmission from NTPs.

Educational information should be prominent and delivered in multiple formats, such as signage in a retail location, verbal education delivered during a sale, placards under animal displays, and printed materials for animal owners to take home. Educational information should also be made widely available electronically, such as on websites and social media platforms.

#### Sample messaging for NTP owners and the public

What follows is sample messaging that can be easily shared by all partners to NTP owners and the public. We recommend including a definition of an NTP in addition to the messages below and where possible, replacing the term NTP for the specific animal species.

***Non-traditional pets include rodents (rats, mice, hamsters, gerbils, guinea pigs, prairie dogs), other small mammals such as hedgehogs and ferrets, backyard poultry, reptiles, amphibians, fish, and other aquatic animal species.***

##### Sample messaging

###### Selecting an appropriate non-traditional pet

- Before getting a non-traditional pet, research the pet to make sure it's a good match for you and your family, especially if you have young kids or older adults in your household. Learn about the animal's size, temperament, behavior, lifespan, housing, costs, and feeding needs. You can also talk to a veterinarian—check your area for one who specializes in exotic animals or backyard poultry, depending on the type of animal you're considering.

Information on **picking the right pet** is available on CDC's Healthy Pets, Healthy People website.

**Species-specific considerations** to help you choose a healthy pet are available on AVMA's website.

- Some pets aren't recommended for people at higher risk of illness, such as children younger than 5 years old, adults 65 and older, people with weakened immune systems, and pregnant people.
- If you decide that a non-traditional pet is the right pet for you, it's very important that you learn how to properly take care of it. You should also be aware that these pets can sometimes carry harmful germs that can make you sick. But with routine veterinary care and some simple habits, you can reduce your risk of getting sick from touching or owning a non-traditional pet.
- When considering a non-traditional pet, first check state, local, and property laws and ordinances before purchasing or adopting. Some animals are illegal to own in certain areas, or there might be restrictions on certain species, animal numbers, animal sex (for example, roosters), or housing.
- When choosing any pet, pick one that appears healthy. In general, animals should be lively and alert with clean and intact fur, feathers, or scales. An animal that is abnormally quiet or tired, has discharge from the eyes or nose, has diarrhea or poop on its body, or looks unhealthy might be sick. If one of the animals in an enclosure looks sick, the others may also have been exposed to an infectious disease.
- Take your new non-traditional pet to a veterinarian within a few days to a week for a first check-up, and continue to take your pet to the vet regularly to keep it healthy throughout its life.
- Keep your new pet separated from your other pets for at least a week to ensure they are healthy, even if they don't look sick. The length of time may vary depending on the type of animal, so work with your veterinarian to determine how long to keep new pets separated from existing pets to help prevent your other animals from getting sick.
- Purchase or adopt captive-bred animals because they are more accustomed to people and living in captivity than animals that weren't bred in captivity. They also should have a better-known health history than wild-caught animals.

**Tips for buying or adopting an NTP:**

- Purchase or adopt from reputable sources.
- Ask about
  - Written pet wellness policies and pet care policies
  - Well-defined animal return policy
  - Well-defined policy to follow if your new pet becomes sick within a specific timeframe
- When buying from a retail store, look for
  - Knowledgeable store employees
  - Clean store (with pleasant smell)
  - Clean habitats
  - Adequate lighting in habitats
- Purchase new backyard poultry from a USDA National Poultry-Improvement Plan flock. Participants in this program must meet certain flock health and sanitation standards (Appendix H. Selected education resources)

Things to **avoid** when purchasing or adopting an NTP:

- Internet sales from unknown sources
- Swap meets and flea markets
- Roadside stands
- Retailers selling animals as a side business

###### Specific animal considerations when selecting an NTP

- **Rodents** (guinea pigs, rats, mice, prairie dogs, hamsters, gerbils) **and some other small mammals** (for example, hedgehogs) (Small Mammals, 2019)
-   Pet rodents and some other small mammals are not recommended for households with children younger than 5 years old, adults 65 and older, people with weakened immune systems, or pregnant people because these groups are at higher risk for serious illness from germs these animals can carry.
-   Do not keep pet rodents and other small mammals in childcare centers, nursery schools, primary schools, or other facilities with children younger than 5 years old or facilities that care for adults 65 and older and those with weakened immune systems (such as long-term care facilities and nursing homes).
- **Ferrets**
-   Ferrets are not recommended as pets in households with children younger than 5 years old.
-   Never leave children unsupervised with a ferret, and don't let ferrets roam freely because of the risk for bites, scratches, and unprovoked attacks.
-   Rabies vaccination is recommended for pet ferrets and is required by some states (Compendium of Animal Rabies Prevention and Control, 2016; State Rabies Vaccination Laws, 2021).
- **Reptiles** (turtles, lizards, snakes)
-   Reptiles are not recommended for households with children younger than 5 years old, adults 65 and older, people with weakened immune systems, or pregnant people because these groups are at higher risk for serious illness from germs these animals can carry.
-   Children younger than 5 years old should not handle or touch reptiles, or anything in the area where they live and roam.
-   Do not keep reptiles in childcare centers, nursery schools, primary schools, or other facilities with children younger than 5 years old or facilities that care for older adults and those with weakened immune systems (such as long-term care facilities and nursing homes).
- **Amphibians** (African dwarf frogs, salamanders)**, fish, and other aquatic animals**
-   Amphibians are not recommended for households with children younger than 5 years old, adults 65 and older, people with weakened immune systems, or pregnant people because these groups are at higher risk for serious illness from germs these animals can carry.
-   Do not let children younger than 5 years old touch aquariums or aquarium water or feed fish or other aquatic animals.
-   Do not keep amphibians in childcare centers, nursery schools, primary schools, or other facilities with children younger than 5 years old or facilities that care for older adults and those with weakened immune systems (such as long-term care facilities and nursing homes).
- **Backyard poultry** (chicks, chickens, ducks, ducklings)
-   Backyard poultry are not recommended for households with children younger than 5 years old, adults 65 and older, people with weakened immune systems, or pregnant people because these groups are at higher risk for serious illness from germs these animals can carry.
-   Do not let children younger than 5 years old handle or touch chicks, ducklings, or other live poultry.
-   Do not keep backyard poultry in childcare centers, nursery schools, primary schools, or other facilities with children younger than 5 years old or facilities that care for older adults and those with weakened immune systems (such as long-term care facilities and nursing homes).
- **Feeder animals** (feeder rodents and chicks)
-   Children younger than 5 years old and pregnant people should not touch or handle frozen or live feeder rodents or other feeder animals.
- **Venomous or toxin-producing animals** should not be kept as pets in any setting.
- **Psittacine birds** (parrots, parakeets, and cockatiels)
- Consult Birds Kept as Pets | Healthy Pets, Healthy People | CDC, the NASPHV—Psittacosis and Chlamydiosis (Thomas et al, 2017) and seek veterinary medical advice.

###### Personal hygiene and safe animal contact

- Always wash your hands thoroughly with soap and running water after handling your pet or its food, habitat, or supplies (When and How to Wash Your Hands, 2022).
- Adults should supervise handwashing for young children.
- Washing hands with soap and water is the best way to reduce the number of germs on them. If soap and water are not available, use an alcohol-based hand sanitizer that contains at least 60% alcohol until hands can be properly washed. Alcohol-based hand sanitizers can quickly reduce the number of germs on hands in some situations, but these products are not effective against all types of germs.
- Avoid bites and scratches from non-traditional pets by learning how to approach and hold the animal safely. Many types of germs can spread from animal bites or scratches, even if the wound does not look serious. If you are bitten, scratched, or pecked by a non-traditional pet, immediately wash the wound with warm, soapy water and contact your healthcare provider. If the animal was sick at the time of the bite or shortly afterwards, then it should be seen by a veterinarian.
- Do not kiss, snuggle, or hold non-traditional pets close to your face.
- Do not touch your face or mouth during or after feeding or handling non-traditional pets until you can wash your hands thoroughly.
- Do not eat, drink, use tobacco products, or put other materials in your mouth while handling non-traditional pets.
- Supervise children when interacting with non-traditional pets.
- Do not let children younger than 5 years old handle non-traditional pets or their food, supplies, or habitats.
- If your or your child are sick or have been scratched or bitten by a non-traditional pet, tell your healthcare provider that you and your family have been around NTPs or other animals, whether at home or away from the home.

###### Specific animal considerations for safe handling

- Handle frozen feeder animals with a set of dedicated tongs to prevent bites and scratches from the pet you are feeding.
- Feed dead feeder animals (fresh or frozen) to pets to prevent injury to yourself and your pet from a live feeder animal.
- To prevent bites, don't handle reptiles right after handling feeder animals because the reptile may smell the animal on you and strike or bite.
- Don't touch fish, other aquatic animals, or their water without gloves on. Wear gloves when handling fish, tank water, and any tank equipment (such as when cleaning the tank) and wash hands thoroughly with soap and water immediately after taking off the gloves (Disease Information—Mycobacteriosis, 2020).
- Pregnant people should not clean rodent habitats or supplies. A non-pregnant adult should routinely disinfect rodent cages and accessories, including used bedding, with a 10% bleach solution or a commercial disinfectant (Prevention. Lymphocytic Choriomeningitis (LCM), 2014). If rodents are suspected to have Seoul virus infection, LCMV infection, rat- bite fever, or other infections, the person cleaning the rodent environment should wear a respirator and gloves and cover scratches or open wounds.
- When cleaning poultry coops or enclosures, wear designated clothing, including work gloves, boots/shoes, and coveralls or other protective clothing that is solely used for working with poultry. Use a face mask and protective glasses or goggles when cleaning dusty areas.

###### Preventing contamination of the home and environment

- Keep non-traditional pets and their supplies out of the kitchen or other areas where food is prepared, served, stored, or consumed. Pets can contaminate surfaces in your home with germs—you don't have touch pets to get sick from their germs.
- Clean supplies, including habitats and accessories, outside the house when possible. If supplies are cleaned indoors, use a laundry or utility sink or bathtub and thoroughly clean and disinfect the area right after. Never use food-preparation areas to clean non-traditional pet habitats or anything in their habitats. To avoid cross contamination, use cleaning materials (sponges, brushes, etc.) dedicated for these purposes and keep them separate from those used for routine household cleaning.
- Do not thaw frozen feeder animals in a microwave or in areas also used for human food preparation. Designate dedicated and separate food storage containers for feeder animals and keep them separate from human food and food containers.
- Do not allow non-traditional pets to roam freely in the home.
- Do not allow backyard poultry in the home, even chicks.
- If a non-traditional pet dies, clean and disinfect its habitat thoroughly. Don't put another animal in that habitat without cleaning and disinfecting it.
- Clean all surfaces that have come in contact with a non-traditional pet thoroughly to remove dirt, poop, pee, or other body fluids before disinfecting. Follow instructions on the label for disinfectants like bleach and quaternary ammonium products. Most disinfectants require more than 10 minutes on a contaminated surface to disinfect completely.

###### Specific animal considerations for preventing contamination of the home environment

- Never feed wild animals to non-traditional pets.
- Do not keep wild animals as pets.
- Safely secure coops, habitats, and other non-traditional pet enclosures to prevent wildlife and other animals from getting in.
- Use separate boots/shoes and coveralls or other designated clothing for poultry coop and enclosure cleaning and store them outside of the home or in a garage, shed, or laundry room away from other clothing. Wash these clothes with detergent and hot water.
- Monitor water quality in aquariums and regularly change the water. Keep sick fish and other aquatic animals isolated from all healthy aquatic animals. If you have a sick fish or other aquatic animal, talk to a veterinarian. Promptly remove sick or dying aquatic animals from the tank, and clean and disinfect the tank with a dilute bleach solution before adding new fish. If there are other fish in the tank, move them to a different tank before cleaning and disinfecting.
- Avoid keeping soft corals associated with palytoxin which is a toxin found in zoanthid and palythoa corals.
- Use caution when cleaning tanks containing soft corals- wear gloves and eye protection while cleaning or handling coral, keep coral submerged when moving between tanks, and wash your hands after they have been in the tank.

###### Pregnant people

- Pregnant people are more likely than other people to get sick from germs certain animals can carry, especially rodents. Germs can also spread to unborn babies or to babies during birth.(Anonymous, n.d.)
- If you are pregnant, do not handle rodents, their supplies, or their habitats.
- If you have a pet rodent, have someone else care for the animal until you are no longer pregnant.
- If you are pregnant, consider waiting to adopt a new non-traditional pet because young children are at higher risk for serious illness from germs these animals can carry.
- Some germs, like lymphocytic choriomeningitis virus, can cause serious illness, birth defects, and lifelong disabilities, such as hearing loss or learning problems in children (People at Higher Risk for Illness from Animals, 2022).

###### Responsible animal ownership, including non-traditional pets

- Responsible pet ownership means providing enough time and resources to ensure a good quality of life for a pet, as well as a healthy setting for the people and other pets in the household (Responsible Pet Ownership, n.d.).
- Research pets before adopting or buying.
- Don't buy a pet impulsively.
- Choose a pet that fits your family and lifestyle.
- Prepare the pet's habitat before its arrival to your home.
- Provide the right food, housing, handling, veterinary medical care, and socialization for the pet to keep it healthy.
- Learn how to properly care for a non-traditional pet, including providing healthy food, daily activities, and the right environment.
- Use species appropriate preventive treatment for intestinal parasites and external parasites such as fleas, ticks, and mites.
- Take your new pet to a veterinarian within a few days to a week for a first check-up, and continue regular visits to keep the animal healthy throughout its life.
- Never release a non-traditional pet into the wild. These animals are not adapted to live in the wild and they can damage the ecosystem. If you no longer want or cannot care for a pet, check with local or regional animal rescues, local animal control, veterinarians, pet shops, or state wildlife agencies to find out how to appropriately relinquish a non-traditional pet.
- Releasing a non-traditional pet into the wild cans spread diseases to native wildlife, outcompete and displace native wildlife, and disrupt ecosystems. It can also put people, pets, and livestock at risk for diseases or injury (Hoyer et al, 2017; Morris, 2009; Sweeney, 2018).
- As a pet owner, keep your pet healthy by only using antimicrobials when prescribed by a veterinarian. Do not save and reuse leftover antimicrobials. Only give antimicrobials to the animal(s) to which they were prescribed (“Antibiotic Use is Changing,” n.d.).
- If you handle venom- or toxin-producing reptiles, amphibians, fish, or invertebrate animals, know how to manage venom or toxin exposures in people and how to prevent animal escapes during disasters. You should also promptly alert appropriate authorities if animals escape. National Poison Information Centers (800-222-1222) are important resources if animal-related intoxications occur in people but should not substitute for immediate evaluation at an emergency department.
- Include non-traditional pets in your emergency response plan like you would for any other pet or family member (Pet Safety in Emergencies, 2021).
- Separate sick people from pets to avoid the spread of diseases such as flu or COVID-19 from people to pets (Animals and COVID-19, 2022; Influenza in Animals, 2018).

#### B. Recommendations for handwashing

Handwashing after contact with animals is associated with decreased rates of illness during disease outbreaks linked to animals and is the most important step for reducing disease transmission associated with animal contact (When and How to Wash Your Hands, 2022). Below are handwashing recommendations for anyone who handles NTPs or has indirect contact with their habitat or supplies. More detailed information and messaging to share with non-traditional pet owners and the public can be found in Appendix E. Handwashing.

**A number of printable resources for a variety of settings are available online to encourage handwashing among adults and children after handling non-traditional pets and other animals.**

- National Association of State Public Health Veterinarians
- Proper Hygiene When Around Animals | Etiquette & Practice | Hygiene | Healthy Water | CDC
- CDC Healthy Pets, Healthy People

Always wash hands thoroughly with soap and water right after

- handling any animal, including NTPs
- after touching anything that was in contact with animals
- after handling animal food, supplies, or waste
- after being around animals or spending time in an animal area, even if you didn't touch an animal.

For example, hands should be washed after handling shoes, toys, water bowls, or other items that may have been in an NTP's environment. After handling an NTP, always wash hands immediately before preparing food and before eating (When and How to Wash Your Hands, 2022).

- Running water, soap, and materials to dry hands should be accessible at all public locations where people interact with NTPs and other animals, including handwashing stations and appropriate signage to remind people to wash their hands.
- If soap and water are unavailable, an alcohol-based hand sanitizer that contains at least 60% alcohol can be used to clean hands until soap and water become available. Hand sanitizer is not effective against all germs and is less effective on visibly soiled hands.
- Adults should supervise children to ensure they are using appropriate handwashing techniques, especially after playing with pets at home or visiting locations where animals are present such as pet stores, agricultural stores, or other retailers where animals are sold, fairs, educational farms, petting zoos, nature parks, birthday parties and other animal entertainment exhibits.

Discourage the following in public areas where people at higher risk of severe illness, including children younger than 5 years old, may come in contact with NTPs and other animals (How to Stay Healthy Around Pets, 2021):

- Eating or drinking
- Use of strollers, toys, pacifiers, baby bottles, or spill-proof cups
- Hand-to-mouth behaviors, such as thumb sucking, nail biting, and chewing gum
- Sitting or playing on the ground in animal areas
- Feeding the animals or sharing food with animals
- Any contact with animals if the person has open wounds or underlying illnesses
- Contact with animal waste

#### C. Antimicrobial use, resistance, and stewardship recommendations

Veterinary care providers, human healthcare providers, and industry partners can slow antibiotic resistance by implementing disease prevention strategies and playing a critical role in the practice of antimicrobial stewardship. Recognizing the significance of antimicrobial resistance in both human and veterinary medicine, many organizations have recommendations and are developing best practices for antimicrobial stewardship (Table 1. Antimicrobial Stewardship Resources).

**Table 1.** Antimicrobial Stewardship Resources

Veterinarians	American Veterinary Medical Association (AVMA)	Antimicrobial stewardship definition and core principles https://www.avma.org/policies/antimicrobial-stewardship-definition-and-core-principles
	AVMA	Veterinary checklist for antimicrobial stewardship Veterinary-Checklist-Antimicrobial-Stewardship.pdf (avma.org)
	AVMA	Judicious therapeutic use of antimicrobials Judicious therapeutic use of antimicrobials | American Veterinary Medical Association (avma.org)
	AVMA	Species report cards Antimicrobial-resistant pathogens affecting animal health | American Veterinary Medical Association (avma.org)
	AVMA	Backyard Chickens 101 Backyard chickens 101 | American Veterinary Medical Association (avma.org)
	AVMA	AAAP guidelines for judicious therapeutic use of antimicrobials in poultry AAAP guidelines for judicious therapeutic use of antimicrobials in poultry | American Veterinary Medical Association (avma.org)
	Centers for Disease Control and Prevention (CDC)	Pets and antibiotic resistance Pets & Antibiotic Resistance | Healthy Pets, Healthy People | CDC
	Food and Drug Administration (FDA)	5-year plan for supporting antimicrobial stewardship in veterinary settings (2019–2023) Supporting Antimicrobial Stewardship in Veterinary Settings (fda.gov)
	American College of Veterinary Internal Medicine (ACVIM)	Consensus statement on therapeutic antimicrobial use in animals and antimicrobial resistance ACVIM Consensus Statement on Therapeutic Antimicrobial Use in Animals and Antimicrobial Resistance—Weese—2015—Journal of Veterinary Internal Medicine—Wiley Online Library
	International Society for Companion Animal Infectious Diseases (ISCAID)	Antimicrobial Guidelines Working Group https://iscaid.org/
	University of Minnesota	Antimicrobial Resistance Learning Site https://amrls.umn.edu/
		Handbook of antimicrobial stewardship in companion animal veterinary settings Handbook of Antibiotic Stewardship in Companion Animal Veterinary Settings | Antimicrobial Resistance and Stewardship Initiative (umn.edu)
	World Health Organization (WHO)	WHO guidelines on use of medically important antimicrobials in food-producing animals http://apps.who.int/iris/bitstream/handle/10665/258970/9789241550130-eng.pd
Industry	The Pet Advocacy Network	Recommendations for antimicrobial stewardship in companion animals https://petadvocacy.org/zoonotic-disease-prevention
Human Healthcare Providers	CDC	Antimicrobial threats in the United States, 2019 https://www.cdc.gov/drugresistance/pdf/threats-report/2019-ar-threats-report-508.pdf
	CDC	Core elements of antimicrobial stewardship Core Elements of Antibiotic Stewardship | Antibiotic Use | CDC

Antimicrobials are valuable tools used to fight infections caused by pathogens such as bacteria, viruses, parasites, and fungi in both animals and people. Improper use or overuse of these medications can contribute to the development and spread of drug resistance which reduces the effectiveness of antimicrobials for the treatment of animal, plant, and human illnesses (Antibiotic/Antimicrobial Resistance, 2021; Antimicrobial Resistance, 2021.; Broens and van Geijlswijk, 2018). When pathogenic and commensal organisms are exposed to antimicrobials, strains that are able to survive can multiply. They can then share their resistance mechanisms that allow them to survive with other pathogenic and commensal organisms that may not have been exposed to the drug. Resistance develops faster when antimicrobial exposure is high. Therefore, antimicrobials for medical care and animal health care should only be used when necessary and in an appropriate manner, including the right drug, dose, and duration as prescribed.

Performing diagnostic testing to identify an underlying cause is essential when deciding whether to use an antimicrobial. Not all pathogens require treatment with an antimicrobial. Bacterial culture enables antimicrobial susceptibility testing, which is helpful for surveillance purposes and for selecting appropriate antimicrobial drugs for treatment.

Culture-independent diagnostic testing (CIDT) methods such as tests that detect a small part of a pathogens' DNA or specific antigens are being used to identify pathogens in both people and animals. Because CIDT methods do not yield isolates, full characterization of pathogens, such as serotyping, antimicrobial susceptibility testing, and molecular testing cannot be done (Iwamoto et al, 2015; Marder et al, 2017). Following up on positive CIDT results with culture will provide isolates that can undergo comprehensive molecular testing, such as whole genome sequencing (WGS). WGS can show which human, animal, and environmental isolates are genetically related, and along with epidemiological data, can provide information about the spread of emerging resistance mechanisms, and potentially identify which human cases have a common animal source.

During an outbreak investigation associated with a non-traditional pet, identifying a common animal source aids in timely response which may prevent additional infections. WGS can also help identify emerging antimicrobial-resistant pathogens.

Public health officials should identify experienced animal handlers such as veterinarians or biologists to assist with animal sampling and specimen collection in an outbreak investigation.

##### Antimicrobial stewardship recommendations for veterinarians

Good husbandry and preventive care can improve a pet's health and reduce the need for antimicrobial treatment. Antimicrobials should only be used in NTPs and other animals when prescribed by a veterinarian under a valid veterinary-client-patient relationship (Veterinarian-Client-Patient Relationship (VCPR) FAQ, n.d.) and consistent with stewardship principles as defined by the American Veterinary Medical Association (AVMA). Judicious use of antimicrobials, use of laboratory testing to determine disease etiology, and culture and susceptibility testing to aid in appropriate antimicrobial selection are critical when antimicrobial therapy is warranted (Judicious Therapeutic use of Antimicrobials, n.d.). Antimicrobials should not be used as a substitute for disease prevention measures such as good animal husbandry and sanitation. Several resources exist to help veterinarians practice antimicrobial stewardship (Table 1. Antimicrobial Stewardship Resources) (Handbook of Antibiotic Stewardship in Companion Animal Veterinary Settings, 2022).

It is critical that veterinarians communicate with non-traditional pet owners about appropriate antimicrobial use when discussing diagnostic and treatment plans for their pets, including the importance of culture and sensitivity to ensure the effectiveness of the antimicrobial. Veterinarians should emphasize to owners their role in preserving the effectiveness of all antimicrobials by not requesting antimicrobials when they are not needed, following instructions when antimicrobials are prescribed, and never using a medication in an animal unless prescribed for that animal (“Antibiotic use is Changing,” n.d., Antimicrobial Stewardship Definition and Core Principles, n.d.).

While they have been considered non-traditional pet species for the purpose of this Compendium, backyard poultry are food-producing animals and as such the animals themselves and their products (e.g., eggs) are subject to oversight by USDA and FDA. Treatment of backyard poultry with medically important antimicrobials given in feed falls under the FDA Veterinary Feed Directive rule and must be under the supervision of a veterinarian with a valid veterinary-client-patient relationship (“FACT SHEET: Veterinary Feed Directive Final Rule,” 2022; “FDA Releases Draft Guidance,” 2021).

##### Antimicrobial stewardship recommendations for industry

A critical component of antimicrobial stewardship for all sectors of industry is ensuring adherence to best practices for prevention and management of disease to ensure animals are healthy, thereby reducing the need for antimicrobials.

- Antimicrobials should only be administered to treat, prevent, or control disease and their use should align with antimicrobial stewardship principles. If antimicrobials are needed, they should only be administered under veterinary medical supervision with a valid veterinary-client-patient relationship in any industry setting, regardless of the size or scale of the operation (Veterinarian-Client-Patient Relationship (VCPR) FAQ, n.d.).
- Breeders, distributors, and retailers including pet and agricultural industry employers should ensure that existing biosecurity measures are sufficient to reduce the risk for potential disease transmission between people and animals, and among animals (Montgomery, 2019).
- Pet stores and other facilities that employ animal care workers should provide education and training on handwashing and other infection prevention measures and provide employees with necessary personal protective equipment for cleaning animal areas.
- Pet stores and other businesses that sell directly to the public should educate customers on handwashing and other prevention measures for decreasing disease transmission in the customers preferred language and at a level understood by the public. Species-specific husbandry and dietary requirements should also be provided to help ensure NTPs are maintained at optimal health.
- Facilities with NTP species should take steps, including cleaning, disinfection, and maintenance of equipment and supplies, to limit environmental contamination of NTP housing.

##### Antimicrobial use and *Salmonella*

Reptiles, amphibians, backyard poultry, and small mammals can carry *Salmonella* bacteria in their intestinal tract and shed it in their feces, even if they appear healthy. Thus, there are circumstances where fecal culture of NTPs may be necessary and recommended (e.g., during an outbreak investigation when there is concern about human transmission). Testing or using culture of fecal specimens or of environmental specimens may also be warranted to characterize circulating strains or to develop vaccines. But because *Salmonella* bacteria are shed intermittently in feces, culture will not detect *Salmonella* infection in all animals.

Healthy animals should not be treated with antimicrobial agents with the intent of eliminating *Salmonella* from the intestinal tract because this can lead to a carrier state in the animal, disrupt the animals' gut flora, and increase the risk of antimicrobial resistance. In cases of known infection, it may be difficult to determine if an animal has cleared an infection after antimicrobial therapy because of the potential for intermittent shedding and false negative test results. Veterinarians should discourage antimicrobial treatment of healthy animals for *Salmonella* if animal owners request it, and they should provide education on health effects of inappropriate use of antimicrobials (including disruption of the microbiome/gut flora), and antimicrobial resistance and its potential effects on human and animal health. Safe handling tips to share with animal owners are available in Appendix H. Selected Educational Resources.

#### D. Occupational Health

Employers play an important role in protecting employees by establishing an occupational health plan that considers workplace specific risks. These occupational health recommendations apply to any place of employment where employees and volunteers may have contact with NTP species or have indirect contact with their habitat or supplies. This may include veterinary clinics, pet stores, agricultural retail stores, breeders, distributors including air and ground shipping employees, and others. Industry leaders with well-developed occupational health programs can serve as a resource to help establish an employee health program that includes training on how pathogens are transmitted from NTPs to people, risk factors, and disease prevention (Table 2).

**Table 2.** Zoonotic Disease Prevention Resources for Employers

Centers for Disease Control and Prevention (CDC)	Healthy Pets, Healthy People www.cdc.gov/healthypets
CDC	Influenza Information for Specific Groups | Avian Influenza (Flu) (cdc.gov)
CDC	National Institute for Occupational Safety and Health Veterinary Safety and Health https://www.cdc.gov/niosh/topics/veterinary/biological.html
Iowa State Center for Food Security and Public Health	The Center for Food Security & Public Health http://www.cfsph.iastate.edu/?lang=en
National Association of State Public Health Veterinarians	Compendium of Veterinary Standard Precautions for Zoonotic Disease Prevention in Veterinary Personnel http://www.nasphv.org/Documents/VeterinaryStandardPrecautions.pdf
Pet Advocacy Network	Pet store pro modules: https://www.petstorepro.com/ http://pijac.org/animal-welfare-and-programs/zoonotic-disease-prevention

##### Occupational health plan

Employers can protect employees by establishing an occupational health plan for all employees who will be working with or around NTP species. The plan should include written and verbal instructions for implementation and documented refresher training on a regular basis. Specific plans depend on the NTP species. In general, an occupational health plan should include the following:

- A qualified, designated point of contact for occupational health in the workplace who is responsible for documenting injuries, illnesses, hazards, and exposures.
- A mechanism for reporting work-related illness and injury including bites and scratches. Employees should be encouraged to report all potential zoonotic illnesses to their supervisor or designated occupational health point of contact, and seek medical care, especially for gastrointestinal illness (e.g., salmonellosis), fungal infection (e.g., dermatophytosis), or illness or infection after being bitten or scratched.
- Information on potential exposures and risk to people in high-risk groups such as adults 65 and older and people with a weakened immune system.
- Information for pregnant people about potential risks.

Employees are more likely to stay home and not infect co-workers, clients, or the animals if they receive paid sick leave. If employees or volunteers have questions about their health or immune status, they should talk with their healthcare provider.

##### Education and training for employees

Employers should provide routine education and training for all employees who will be working with or around animals on basic principles of disease prevention. This should include education at the time of hiring and refresher training on a regular basis (e.g., annually), before a change in job duties, or if new procedures are implemented. Training should include information on animal husbandry, handling, feeding, zoonotic disease risks, handwashing, personal protective equipment use, and other biosecurity protocols. Employers can find resources on inclusive communication principles to employ in training materials here.

A retail pet store employee is often the first person a potential NTP owner may interact with to learn about these pets, therefore it is critical for employers to ensure these employees are trained on safe handling, zoonotic disease risks, prevention, and how to communicate this information to pet owners.

- Employees should be aware that NTP species can appear healthy while carrying some zoonotic pathogens such as *Salmonella*.
- Employees should be trained to recognize symptoms of illness in both the NTP species they are working with and in people and what to do if they experience such symptoms.
- Employees should be fully trained to safely handle NTP species and know about zoonotic disease risks that are common or possible with the species they are working with or around (Background section). For example, only employees trained in handling poultry and *Salmonella* prevention should be in contact with poultry displays or housing areas.

##### Biosecurity and personal protective equipment (PPE) in the workplace

Biosecurity refers to measures taken to prevent transmission of pathogens, including bacteria, viruses, fungi, and parasites, to people and animals. Biosecurity measures may be structural, such as physical barriers, or they may be operational, such as procedures, practices (e.g., use of PPE), and policies that aim to prevent disease (Defend the Flock- Biosecurity 101, n.d.). All employees should follow basic biosecurity practices, including handwashing (Appendix E. Handwashing). Facilities should have a written biosecurity protocol in conjunction with or in addition to the employee health plan.

**Examples of biosecurity measures for NTP species:**

- Limit the number of people who have contact with animals.
- Wash hands before and after handling animals, even if using gloves.
- Wear disposable boot covers and use disinfectant footbaths upon entering and leaving an animal area.
- Change clothes before entering or leaving an animal area.
- Clean and disinfect all tools and equipment before moving them between animal areas.

- In industry settings with multiple animal species, employees should work in only one area of a facility. If employees service multiple areas, they should follow biosecurity practices appropriate to each animal or area.
- NTP species should be kept separate from areas where human food is prepared, served, or consumed.
- Staff should not use shared refrigerators or freezers for feeder animals or animal carcasses (such as those awaiting postmortem or removal) and food for human consumption.
- An adequate number of handwashing stations should be placed at locations close to where they should be most heavily used.
- Facilities should have clearly designated areas for animal breeding or handling to reduce risks of environmental contamination and subsequent transmission to humans.

PPE is equipment worn to prevent exposure to hazards that cause workplace injuries and illnesses that may result from direct or indirect animal contact (Personal Protective Equipment—Overview, n.d.). All PPE should be safely designed, constructed, and maintained in a clean and reliable fashion, and fit properly. For PPE to be most effective it should be worn correctly. If PPE is to be used in a workplace, a PPE program should be implemented that addresses the hazards present; selection, maintenance, and use of PPE; employee training; and program monitoring. Employers are required to provide or pay for most types of PPE (1910.132—General requirements, n.d.).

**Examples of PPE to consider when working with NTP species:**

Rodents and other small mammals, including feeder rodents:

- Gloves, cap, and gown (for suspected or confirmed infected or ill animals)
- N95 respirator or equivalent or higher-level respirator (in case of suspected or confirmed infection with LCMV or Seoul virus)
- CDC provides PPE guidance for veterinarians and veterinary staff handling a rat known to be infected with Seoul virus

Reptiles, amphibians, & other aquatic species:

- Gloves, cap and gown (in case of suspected or confirmed infected or ill animals)

Backyard poultry and poultry used as feeder animals:

- Work gloves
- Rubber boots
- Washable or disposable coveralls
- Face mask in dusty environments
- N95 respirator or equivalent or higher-level respirator (suspected or confirmed poultry with respiratory disease)

- Employers should consider the NTP species, setting, potential zoonotic pathogens, and exposure risks depending on job task when selecting the appropriate level of PPE for employees.
- Employees should wear appropriate PPE when working with infected (or potentially infected) animals.
- Gloves may be used in certain situations, such as to protect the handler from bites and scratches or to protect the animals' skin (certain amphibians). Gloves may be used to prevent zoonotic disease transmission when handling an infectious animal, used bedding, or dirty cages (Zoonotic Disease Prevention, n.d.). Gloves may be work gloves for handling animals or disposable (e.g., latex, nitrile, vinyl), depending on the activity.
- Employees should wash hands immediately after removing gloves.
- Employers should require employees to wear a respirator (i.e., N95 or equivalent or higher-level of protection) if one is needed to prevent pathogen transmission (e.g., in the case of suspected or confirmed LCMV or Seoul virus infections in a rattery, or in backyard poultry with possible avian influenza infections).
- Where respirators are required, a respiratory protection program should be implemented that includes medical clearance, training, and fit-testing (1910.134—Respiratory Protection, n.d.).
- Employers should provide employees training on proper use of PPE, with regular refresher training (e.g., annually or if the PPE worn changes). Training should include information on:
  - When PPE is necessary
  - What kind of PPE is necessary
  - How to properly put PPE on, adjust, wear, and remove it
  - The limitations of the PPE
  - Proper care, maintenance, useful life, and disposal of PPE
- Employers should be aware that in emergency situations (e.g., the COVID-19 pandemic) PPE shortages may occur and if so, to consult with their state public health veterinarian for PPE recommendations. An up-to-date list of contact information for state public health veterinarians is available on the National Association of State Public Health Veterinarians (NASPHV) website.

##### Required use of respiratory protection

If hazards in the workplace indicate the need for respiratory protection, the Occupational Safety and Health Administration (OSHA) requires the employer to establish and maintain a written respiratory protection program (1910.134—Respiratory Protection, n.d.).

The main elements of the respiratory protection program include

- Identification of a qualified, designated respiratory protection program manager who is responsible for maintaining and reviewing the program annually.
- A requirement for initial training when employees are hired and annual refresher training (e.g., proper donning, doffing, strap placement).
- A requirement for employees to undergo medical evaluation and clearance prior to initial respirator use and at least annually afterwards. Employees should be encouraged to report any symptoms experienced while wearing a respirator to a medical provider and the respiratory protection program manager.
- Clear instructions for when filtering facepiece respirators are required.
- An agreement that the employer will provide adequate supplies of disposable filtering facepiece respirators at no cost to employees to prevent reuse.
- A respirator fit-testing plan that includes quantitative fit testing which provides an objective measure of respirator fit.
  - If quantitative fit testing cannot be done, use odor or flavor challenges for qualitative fit testing rather than irritant smoke.
    - More information about qualitative fit testing can be found in the ANSI/AIHA/ASSP Z88.10-2010 Respirator Fit Testing Methods Standard and in the OSHA Respiratory Protection Standard 29 CFR 1910.134 Appendix A.
- A requirement that employees be clean shaven when wearing a tight-fitting respirator so a proper seal and fit can be maintained.
- Maintenance information for reusable respirators (such as half-mask elastomeric or powered air-purifying respirators) if they are used, including a plan to ensure that respirators are properly cleaned, maintained, and stored and that the appropriate respirator cartridges are always changed when necessary.
- A plan to ensure medical clearance, appropriate fit testing, and proper training for reusable respirators (such as half-mask elastomeric or powered air-purifying respirators) if they are being used.

##### Voluntary use of respiratory protection

The employer should provide a copy of Appendix D of the OSHA Respiratory Protection Standard to employees who wear respirators voluntarily (when it is not required by the employer) and encourage employees who voluntarily wear respirators to follow all aspects of proper respirator use including being clean shaven when using a tight-fitting respirator to ensure a good seal.

In addition, the employer must establish and implement those elements of a written respiratory protection program necessary to ensure that any employee using a respirator voluntarily is medically able to use that respirator, and that the respirator is cleaned, stored, and maintained so that its use does not present a health hazard to the user.

Guidance and best practices for reducing zoonotic disease risk related to NTPs in industry settings have been developed by partner organizations:

- CDC has online recommendations based on investigations into prior multistate outbreaks and maintains a list of zoonotic outbreaks.
- USDA has online resources for backyard poultry biosecurity.
- National Association of State Public Health Veterinarians (NASPHV) provides recommendations in the *Compendium of Measures to Prevent Disease Associated with Animals in Public Settings, 2017*.
- The Pet Industry Joint Advisory Council (PIJAC), which includes member organizations from all segments of the pet industry, including breeders, distributers, and retailers, has best management practices for industry regarding husbandry, bedding, facilities, nutrition and diet, disease prevention and management, transportation, euthanasia, sanitation, and disposal.

##### Employee vaccination policies and record keeping

Employers should maintain up-to-date emergency contact information and staff records including vaccinations in accordance with ACIP recommendations and rabies virus antibody titers when indicated (e.g., for those employees working with or around animals that can carry rabies). Employee health information should be collected on a voluntary basis and confidentially maintained. Employees should inform their supervisors of changes in health status, such as pregnancy, that may affect work assignments. New employees should receive training regarding the importance of informing their healthcare provider that their work duties involve contact with animals.

Employees should follow ACIP recommendations and any state or local regulations regarding employee vaccination for rabies, influenza virus, tetanus, SARS-CoV-2, and any other relevant vaccine preventable pathogens that may be encountered in the workplace. NASPHV's Compendium of Veterinary Standard Precautions for Zoonotic Disease Prevention in Veterinary Personnel provides more information on employee vaccination in veterinary settings.

### III. Partner-specific recommendations

#### A. Industry: breeders, distributors, and retailers

NTP owners may use breeders, distributors, and retailers (including those that operate online) as a resource even after an NTP is brought home or to another setting. Those in the NTP industry thus play a critical role in ensuring animal and human health and protecting the environment. The following recommendations for industry are from existing guidance and best practices for reducing zoonotic disease risk related to NTPs in industry settings.

##### Record keeping

Breeders, distributors, and retailers should establish and maintain a record system for traceability of animals during movement from breeder to distributor and retailer for all animals.

At a minimum, records should include the following:

- Date and source at arrival/shipment
- Species type, age, and number of animals shipped
- Specific location within facility where shipped animals were housed (if applicable)
- Medications administered, including antimicrobials and parasite prophylaxis
- Medical and death records
- Destination of animals
- Carrier, if traveling by commercial shipment. This may include vehicle or flight identification, time of departure and arrival, and other stops made.

Poultry retailers should inquire with the hatchery about the source of origin of the birds for recordkeeping/traceability (drop shipping).

For feeder animals, records might include lot numbers for packages of feeder animals to assist in minimizing the scope of recall or euthanasia in the case of an outbreak.

##### Animal health and husbandry

###### Routine animal care

Appropriate veterinary medical care of any animal improves animal health and welfare and reduces zoonotic disease risks. Breeders, distributors, and retailers should

- Work with a veterinarian to establish animal health and disease management plan specific to their facility that prioritizes disease prevention and judicious use of antimicrobials, vaccination protocols, parasite prophylaxis, and testing for zoonotic pathogens as recommended and appropriate to the species in question.
- Monitor animals daily for signs of illness and ensure that animals receive appropriate veterinary medical care in a timely manner.
- Consult a veterinarian when an animal in their care shows signs of illness.
- Avoid exhibiting or selling sick animals; animals known to be infected with a zoonotic pathogen; and animals from groups with a recent history of abortion, diarrhea, or respiratory disease.
- Display or sell only animals that are of appropriate age based on the species.
- House animals in a manner that minimizes stress and overcrowding, which helps to decrease shedding of pathogens.

###### Vaccination

Breeders, distributors, and retailers should consult with a veterinarian on vaccine recommendations or requirements for certain NTP species. House all NTPs in a manner that reduces potential exposure to wild animals that can serve as rabies virus reservoirs such as racoons, foxes, coyotes, and other wildlife. Keep mammals up-to-date on rabies vaccinations according to current recommendations. Effective biosecurity is also needed in addition to any vaccination program for disease prevention.

###### Testing and quarantine for zoonotic pathogens

Routine testing for zoonotic pathogens should not replace other prevention measures because NTPs can shed pathogens intermittently. Test results are not always reliable especially for pathogens that are shed intermittently and test results do not account for previous or subsequent infection and transmission.

Small mammals such as hedgehogs and guinea pigs are different than reptiles in that testing programs may be implemented in breeding programs to reduce the risk of *Salmonella* carriage in breeding populations. Breeding animals are tested over time, and those that test positive are removed from breeding.

The backyard poultry industry uses testing programs extensively among breeding stock to prevent strains of *Salmonella* from being passed from hens to chicks.

Testing has been successfully implemented in certain animal populations to help reduce the burden of *Salmonella,* and to target sanitation, vaccination, or other prevention programs.

Breeders, distributors, and retailers should consult with a veterinarian about appropriate testing and quarantine for zoonotic pathogens in newly acquired breeder animals or other animals.

*Species-specific recommendations*

- Employees of rodent breeding facilities of all kinds should be aware of the risks posed by exposure to rodents infected with LCMV (Knust et al, 2014). When LCMV infections or LCMV antibodies are detected in rodents or employees of a rodent breeding operation, all animal-handling personnel should wear protective equipment, including an N95 respirator or equivalent or higher-level protection. PPE has been shown to reduce the risk of infection. Pregnant people and immunosuppressed persons should not handle animals infected or potentially infected with LCMV.

Some commercial laboratories have developed pathogen detection mechanisms and implemented management practices to prevent contact and disease transmission between rodent breeding colonies and wild mice.

- The US Department of Health and Human Services and the Federation of European Laboratory Animal Science Associations recommend routine virologic and serologic monitoring to detect pathogens, including LCMV. Facilities producing rodents for the pet and feeder-rodent industries should adopt these practices to avoid such outbreaks.
- Breeders, distributors, and retailers should establish quarantine procedures for new animals in consultation with a veterinarian, based on the animal species and disease-specific information, including incubation times. Facilities should quarantine animals surrendered to retail locations, breeders, or any other establishment.
- Rattery owners should quarantine any newly acquired rats for 4 weeks and test these rats for antibodies to Seoul virus before allowing them to comingle with other rats. Commercial laboratories can perform Seoul virus testing of rodent blood samples, and comparisons of results from shared samples have been concordant with CDC laboratory results (Kerins, 2019).

###### Preventing cross-contamination

Contamination of surfaces and other objects with infective pathogens can occur after contact with animals, equipment, housing materials, and other supplies. Such cross-contamination has been linked to reported zoonotic disease outbreaks in people such as *Salmonella* infection. Therefore, it is important to follow cleaning and disinfection recommendations in addition to the following:

- All retailers should document and train staff on cleaning and disinfection protocols.
- Facilities that raise multiple species of animals should establish separate areas for each species. For example, rodents and reptiles should be physically separate and where practical, employees should not work in both facilities or move between facilities without adherence to appropriate established biosecurity protocols.

*Species-specific recommendations*:

- Rodents:
  - To prevent transmission to humans, CDC recommends euthanasia in accordance with AVMA guidelines (AVMA Guidelines for the Euthanasia of Animals, n.d.) of all rats in facilities with human or rat Seoul virus infections. Facilities should obtain further guidance on methods to eradicate Seoul virus from infected ratteries from local or state health departments (Kerins, 2019).
  - Facilities breeding and selling feeder animals should designate specific rooms for feeder animal activities including euthanasia, packaging, and shipping. They should not allow eating, drinking, or smoking in animal rooms or designated areas for euthanasia, packaging, and shipping of feeder animals. Additionally, they should keep animal feed and medications as well as animal carcasses in refrigerators or freezers separate from human food. They should consider measures to reduce zoonotic pathogens in feeder animals, such as irradiation for frozen feeder animals (Electronic Code of Federal Regulations, n.d.).

###### Cleaning and disinfection

Breeders, distributors, and retailers should tailor cleaning and disinfection practices to specific situations. When a particular organism is present, breeders, distributors, and retailers should refer to additional guidance regarding specific disinfectants (Disinfection—CFSPH, n.d.; Selected EPA-Registered Disinfectants, 2022).

- Staff using appropriate personal protective equipment (e.g., eye wear or a face shield to avoid splashes to the eyes and PPE recommended by the disinfectant manufacturer) should clean all surfaces thoroughly to remove organic matter before disinfection. Dusty material should be moistened before removal to reduce aerosolized dust. Animals should also be moved to avoid injuries from the disinfectants.
- Disinfectants such as bleach and quaternary ammonium compounds should be used in accordance with the manufacturer label. Commercial formulations based on chemicals with disinfecting capability must be registered with EPA or cleared by FDA. In most instances, a given product is designed for a specific purpose and should be used in a certain manner. Therefore, users should read labels carefully to ensure they select the correct product for the intended use and apply it efficiently. Most compounds require at least 10 minutes of contact time with a contaminated surface to achieve the desired result.
- Breeders, distributors, and retailers should establish a cleaning and disinfection protocol and schedule for all animal habitats and supplies. They should post these or make them available to employees. Staff should clean and disinfect cages, racks, feeders, watering devices, and other equipment between new batches of animals to keep them clean and free of contamination. Housing areas for animals should be clean and free from equipment, other animals, dirt, and debris. Staff should not use cleaning items such as mops, brooms, and buckets in more than one animal room or housing area to avoid spreading pathogens from one room or area to another. They should dispose of animal waste and dead animals safely and in compliance with local ordinances.
- Breeders, distributors, and retailers should establish safety, hygiene, and infection control protocols and education for all staff on zoonotic disease risks, transmission routes, and prevention measures such as handwashing (Appendix E. Handwashing).

*Species-specific recommendations*

- Rodents:
  - To help reduce the risk of zoonotic disease transmission from feeder animals, some breeders irradiate frozen feeder rodents at the request of some national retail chains (“Salmonella, Feeder Rodents, and Pet Reptiles,” 2020). The Pet Advocacy Network collaborated with One Health partners to develop best management practices for breeders and distributors of feeder rodents (Hardin, 2013). These include guidelines for proper cleaning procedures, habitat, diet, breeding, and shipment procedures, all aiming to reduce the risk of zoonotic disease transmission.
  - Safe handling instructions that are added to packages of frozen feeder rodents may help reduce the risk of *Salmonella* infection (Cartwright et al, 2016).
  - Rodent producers and distributors should keep detailed sales and shipment records and clearly label packages of frozen feeder rodents to allow for more efficient identification and traceback of potentially contaminated products.
- Backyard poultry:
  - Retail stores should carefully evaluate the *Salmonella* control plan of mail-order hatcheries that they purchase from and check to see if they participate in USDA's National Poultry Improvement Plan (Nichols et al, 2018).
  - Retail stores should understand the source hatcheries' practices for drop-shipping, multiplying, and trans-shipping, which can increase the risk of *Salmonella* introduction (Appendix C. Glossary and Appendix D. Industry layout) (Nichols et al, 2018).
  - Retailers should keep animals from different shipments separated and not comingle birds of different ages.
  - Poultry displays in retail stores should be in areas that can be adequately cleaned and disinfected.
  - Poultry displays in retail stores should have barriers to prevent the public from having contact with poultry and to keep poultry away from high traffic areas.
  - A handwashing station with soap, water, and paper towels or hand sanitizer should be in close proximity to poultry for sale and available to staff and the public.
  - High-touch surfaces and poultry enclosures should be cleaned and disinfected regularly and between batches of birds with chemicals that kill *Salmonella* bacteria and avian influenza viruses, and are safe around animals (Nichols et al, 2018).

*Cleaning and disinfection resources for poultry retailers and hatcheries:*

- *USDA Best Management Practices Handbook: A Guide to the Mitigation of* Salmonella *Contamination at Poultry Hatcheries*
- USDA Biosecurity for the Birds, Defend the Flock.
- Backyard Poultry | Healthy Pets, Healthy People | CDC
- Preventing Human Salmonella Infections Resulting from Live Poultry Contact through Interventions at Retail Stores

All hatcheries, including mail-order hatcheries, should participate in USDA's National Poultry Improvement Plan (NPIP) *Salmonella* Monitoring Program

All hatcheries that share eggs or birds should verify participation in the *Salmonella* Monitoring Program.

###### Prevent parasite and pathogen introduction

To prevent parasite introduction, it is important to maintain appropriate barriers to prevent entry of or contact with wild rodents, insects, birds, reptiles, and other animals. Breeders, distributors, and retailers should communicate biosecurity measures to all staff to minimize the risk of pathogen introduction from wild animals.

- Staff should not return escaped animals to the colony unless captured immediately to avoid exposure of the colony to introduced zoonotic pathogens from wild animals.
- Animal feed should be stored in sealed, pest-proof containers and stored off the floor to avoid attracting wild animals.

*Species-specific recommendations*

- Imported reptiles can introduce exotic ticks with potential negative impacts on other pets, agricultural animal industries, and human health (Burridge and Simmons, 2003; Florida's Nonnative Fish and Wildlife, n.d.; Mendoza-Roldan et al, 2020; Pettit, 2018; Sweeney C, 2018; Turtles | Bringing an Animal into U.S., 2022; Unger F et al, 2017). Breeders, distributors, and retailers should follow recommendations to reduce this risk by including tick control in a facility biosecurity plan.

###### Role during outbreak investigations, and reportable or notifiable diseases

Industry plays an important role during an outbreak or case investigation of potential, suspected, and confirmed human illness. Involvement could include assisting federal, state, or local public health departments or departments of agriculture in tracing animal movement to determine the source and extent of the outbreak and implementing prevention practices. Additionally, industry can increase awareness of risks and prevention measures needed among potentially affected consumers, as well as others in the supply chain. Public health officials should include industry notification of an outbreak as part of their standard operating procedures. Industry should be familiar with appropriate points of contact for zoonotic diseases at their state or local public health departments. These points of contact can assist with testing and treatment recommendations, and when to report conditions to local or state authorities.

#### B. Human healthcare and veterinary care providers: A One Health approach

Veterinarians, physicians, and other healthcare providers play an important role in educating animal owners on potential zoonotic disease risks and prevention measures. They are also critical during outbreak or infection investigations, as they can obtain information about animal ownership and animal contact. It is important for healthcare providers to be aware of zoonotic disease risks and to counsel people at higher risk for zoonotic infection or serious consequences from infection. When counseling patients at high risk of serious illness from or infected with a possible zoonotic disease, healthcare providers must consider the human-animal bond as a source of support for their patients. Recommendations should adequately weigh the benefits of interacting with or owning these animals against the risks.

##### Picking the right pet

Together, veterinarians, physicians, and allied healthcare professionals such as nurses can help animal owners select appropriate non-traditional pets, educate them about zoonotic disease risks and prevention measures, and provide advice on who is at higher risk of illness. For species-specific information, please see Appendix F. Guidelines for animals in schools, childcare settings, and long-term care and assisted living facilities, and the General recommendations for all partners, awareness and education, sample messaging for NTP owners and the public section. The sample messaging for NTP owners and the general public has information that can be easily shared with current or potential NTP owners to help them determine the pet that is right for them.

Physicians and other human healthcare professionals can educate patients and their families on the zoonotic disease risks associated with keeping reptiles, amphibians, backyard poultry, and small mammals as pets. They play an important role in advising patients who may have specific health concerns that would make them more vulnerable to a zoonotic disease. Veterinarians can also provide information on zoonotic disease risks as well as animal-specific information such as animal behavior and temperament, husbandry, welfare needs, required veterinary care, and overall pet suitability. Veterinarians serve as a valuable resource for physicians and other healthcare professionals to refer to for advice on counseling patients. Veterinarians can also inform NTP owners that they should tell their healthcare providers about what pets they have at home and any animals they come in to contact with outside the home. Many veterinarians offer a pre-purchase visit for clients who would like to discuss appropriate pets or to examine an animal before it is purchased. However, many times a pet owner will bring an animal to the clinic for an exam after it is purchased, this first visit is also an important opportunity to educate pet owners. Pet industry partners, including retailers, also have resources for pet owners on choosing the appropriate NTP.

To reduce the possibility of illness or injury from NTPs, healthcare professionals should remind parents about matching the size and temperament of a pet to the age and behavior of children, providing close supervision of young children, and educating children about appropriate human-animal interactions (Pickering et al, 2008). People thinking about having children should take pet selection and the pet's lifespan into consideration when thinking about adopting NTPs. These reminders are especially important for patient populations at highest risk of infection or serious consequences of infection, including children younger than 5 years old, adults 65 and older, and people with weakened immune systems, including patients undergoing chemotherapy, organ transplant patients, or pregnant people.

**Human healthcare providers: Ask about pet ownership during a regular check-up or in the case of a suspected infectious disease.**

In addition to mitigating zoonotic disease risks, asking about pet ownership has also been found to open communication with patients. In a recent study, two-thirds of participating primary healthcare providers identified positive effects on practice and on relationships with patients when they asked about pet ownership (Hodgson et al, 2017).

Questions might include the following:

- Do you have pets at home? (Ask specifically about non-traditional pets.)
- How many and what type?
- Who is the primary caretaker for the pets?
- Where are they kept?
- Have they been sick?
- Do they receive routine veterinary medical care?

##### Considerations for suspected cases of zoonotic diseases

As a part of both regular check-ups and in the case of a suspected infectious disease, human healthcare providers should collect a thorough history of animal ownership and animal contact, including in public settings such as exhibits, petting farms or zoos, classrooms, and fairs. Obtaining animal ownership and contact history can lead to specific testing, an accurate diagnosis, and specific treatment recommendations (Day, 2016; Hodgson et al, 2017). It is important to include animal species when taking the history of animal contact (e.g., did you come in contact with rodents, backyard poultry, fish or their habitat etc.?) because in some instances people do not consider these animals as pets, but asking about the species will remind them they should be included (D. Crum, personal communication, Maryland Department of Health).

Upon suspicion or confirmation of a zoonotic disease in a pet, veterinarians should recommend follow up by the pet's owner with a healthcare provider, especially in cases of reported symptoms or illness in owners that may be related to contact with the animal. In these cases, veterinarians should provide information and recommendations on steps that the pet owners can take to reduce the risk of disease transmission from the pet to themselves and anyone else encountering the animal. This includes the information provided above in the General Recommendations section.

##### Role during outbreak investigations, and reportable or notifiable diseases

In the case of a known or suspected zoonotic disease outbreak, it is important for both human and animal healthcare providers to cooperate with authorities conducting the investigation. Healthcare providers and veterinarians should be familiar with appropriate points of contact for zoonotic diseases at their state or local public health departments. These points of contact can assist with testing and treatment recommendations.

Physicians, veterinarians, and other healthcare professionals should be aware of the conditions and diseases they are required to report to local or state public health authorities as well as when to report conditions that may not be specified on the list but should still be reported (e.g., outbreaks of an unknown illness). They should report suspected cases of human or animal illness to the appropriate authority as soon as possible in compliance with local or state laws. Some reporting requirements are statewide, while others are determined by the local health jurisdiction.

#### C. Industry groups, associations, and other affiliated organizations

The NTP industry includes diverse partner organizations, a broad range of business models of varying sizes and types, and hundreds of animal species. Because of this variety, industry groups, associations, and other affiliated organizations (e.g., labor organizations) representing these groups serve as valuable resources to public health agencies in preventing zoonotic disease outbreaks as well as during outbreak investigations and response. Industry groups, associations, and other affiliated organizations should be aware of the conditions and diseases they are required to report to local or state public health authorities as well as when to report conditions that may not be specified on the list but should still be reported (e.g., outbreaks of an unknown illness). These groups should encourage proactive reporting of illness outbreaks among their member organizations and cooperate with outbreak investigations to help limit the spread of disease. Doing so benefits their membership by protecting both animal and human health.

##### Role during outbreak investigations, and reportable or notifiable diseases

During an outbreak or case associated with NTP species, these groups may help facilitate communication and contact tracing of animal movement between those in the pet industry such as breeders, distributors, and retailers with the appropriate government agency. They can help to determine the source and extent of the outbreak, implement prevention practices, and increase awareness among potentially affected consumers as well as others in the supply chain. Effective communication between these groups and government agencies helps to improve both animal and human health and reduce economic impacts of zoonotic disease outbreaks. Access to these networks and building positive relationships can help government agencies during and after an outbreak investigation because many times they include hard-to-reach populations.

##### Role in prevention messaging

Industry groups, associations, and other affiliated organizations can use their knowledge and expertise to collaborate with government agencies to help inform and distribute prevention messaging. Industry groups can develop best practice guidelines for animal species based on current science and public health issues. They should disseminate these messages throughout industry and association networks to reach as many partners as possible with consistent and effective recommendations.

#### D. Government agencies

Government agencies play a critical role in preventing, detecting, and responding to infectious and zoonotic disease outbreaks. Prevention and control of zoonotic diseases associated with NTP species requires a One Health approach by government agencies at all levels—federal, state, local, and tribal—as well as collaboration with multisectoral partners.

##### Education and outreach

Public health agencies, agriculture health agencies, and environmental health agencies should collaborate to develop and disseminate infection prevention messaging, to develop data collection and surveillance programs, and to collaborate during outbreak investigations and response efforts. In the event of a suspected or confirmed outbreak associated with animal contact from an NTP species agencies can notify industry groups or associations to allow them to respond and work with agencies to stop the spread of disease. These industry groups have access to networks of breeders, distributors, and retailers, and access to these networks may be able to help inform the outbreak investigation as well as disseminate information to all involved in the industry and supply chain.

Government agencies should include zoonotic disease prevention education and outreach as part of regular agency duties. These outreach and education efforts can be enhanced by partnering with groups such as community leaders, animal or human healthcare providers, animal or human health professional associations, industry associations or groups, academic institutions including health professions' education programs (e.g., for veterinarians, physicians, physicians' assistants, nurses, and allied health professions), and others. These groups can help to reach a wider range of partner populations, provide subject matter expertise, and provide input on the feasibility of recommendations to help ensure individuals will follow them. Agencies should conduct surveillance activities for human infections associated with contact with NTP species to help evaluate and improve these recommendations.

##### Oversight of regulations or recommendations

Government agencies play a critical role in preventing, detecting, and responding to zoonotic disease outbreaks, including by enforcing regulations, recommendations, or advisories with the goal of preventing and controlling infectious disease threats. Government agencies should encourage or require oversight by the appropriate authority (e.g., local public health or animal health agencies, industry groups, or associations) to ensure compliance with recommendations at various public settings at which NTP species are present, such as backyard poultry retailers, pet stores, petting zoos and farms, schools, daycares, educational animal shows, and others. Agencies should consult *The Compendium of Measures to Prevent Disease Associated with Animals in Public Settings, 2017* for further recommendations for disease prevention for animal exhibitors and venue operators. All appropriate venues should be encouraged or required to ensure compliance with recommendations to reduce the risk to human health from zoonotic diseases transmitted by NTP species.

##### Outbreak response

During an outbreak response, involved sectors should take the following steps as appropriate:

- Conduct thorough epidemiologic, laboratory, and traceback investigations of outbreaks using a One Health approach coordinating across human, animal, and environmental health sectors.
- Follow protocols for collection and laboratory testing of samples from people, animals, and the environment, including molecular subtyping of pathogen isolates.
- Include questions on disease report forms and outbreak investigation questionnaires about exposure to animals and their sources, environments, products, husbandry, and feeding.
- Share outbreak and investigation information across all pertinent agencies, consistent with confidentiality and legal limitations.
- Local and state public health departments should also report all outbreaks of enteric infections resulting from animal contact to the CDC through the National Outbreak Reporting System.
- Local and state public health departments should notify CDC of diseases with severe consequences or that involve multiple states or jurisdictions that are not part of a national reporting system.
- Notify additional relevant partners such as industry and public interest groups and others to promote messaging to partners.

### IV. Summary

As ownership of NTP species is increasing, the number and size of outbreaks associated with NTPs in recent years has also increased. Non-traditional pet owners and members of the public who may come into contact with NTPs should be aware of the potential health risks and understand that even apparently healthy animals can transmit pathogens. The recommendations in this Compendium provide public health professionals, animal health professionals, industry, and healthcare providers (including veterinarians, physicians, and allied health professionals) resources to prevent disease transmission and spread. These recommendations aim to benefit all partners by preventing human infections, maintaining animal health and welfare, and providing economic benefits.

### V. Co-Authors & Acknowledgments

#### Co-authors

**NASPHV Compendium Committee**

- Jennifer A. Brown, DVM, MPH, DACVPM; NASPHV
- John Dunn, DVM, PhD, Co-Chair; NASPHV
- Beth Lipton, DVM, MPH, CPH; NASPHV
- Danielle Stanek, DVM, DACVPM, Co-Chair; NASPHV

**NASPHV Compendium Consultants**

- Kate Varela, DVM, MPH, DACVPM; CDC National Center for Emerging and Zoonotic Diseases (NCEZID)
- Casey Barton Behravesh, MS, DVM, DrPH, DACVPM; CDC NCEZID
- Helena Chapman, MD, MPH, PhD; University of Florida College of Medicine Division of Infectious Diseases and Global Medicine, American Association for the Advancement of Science at NASA Applied Sciences
- Terry H. Conger, DVM, PhD; U.S. Department of Agriculture Animal (USDA) and Plant Health Inspection Service (APHIS) Veterinary Services
- Tiffany Vanover, BBA; International Poultry Retail Consultant
- Thomas Edling, DVM, MSpVM, MPH; American Humane, Pet Advocacy Network Consultant
- Stacy Holzbauer, DVM, MPH, DACVPM; Minnesota Department of Health and CDC Preparedness and Response, Career Epidemiology Field Officer Program
- Angela M. Lennox, DVM, DABVP-Avian, Exotic Companion Mammal; DECZM-Small Mammal; Avian and Exotic Animal Clinic (Indianapolis)
- Scott Lindquist, MD, MPH; Washington State Department of Health
- Suzan Loerzel, DVM, PhD; U.S. Department of Agriculture Animal and Plant Health Inspection Service Animal Care
- Shelley Mehlenbacher, DVM, MPH, DACVPM; U.S. Animal Health Association
- Mark Mitchell, DVM, MS, PhD, DECZM (Herpetology); Louisiana State University School of Veterinary Medicine, Veterinary Clinical Sciences
- Michael Murphy, DVM, JD, PhD; FDA Center for Veterinary Medicine
- Christopher W. Olsen, DVM, PhD; AVMA Council on Public Health and University of Wisconsin-Madison School of Veterinary Medicine Department of Pathobiological Sciences
- Cody M. Yager, DVM, MPH; USDA APHIS Animal Care

## Appendices

### Appendix A. NTP Literature Review and National Outbreak Reporting System (NORS)/Animal Contact Outbreak Surveillance System (ACOSS) Data Request Results

For the purposes of this Compendium, an outbreak was defined as 2 or more unrelated illnesses with an epidemiologic link, and a single illness was considered a case report.

### Literature review inclusion and exclusion criteria (literature review conducted in 2017):

- **Inclusion criteria:**
  - Domestic outbreak (1 or more cases) in people due to animal exposure (both confirmed and unconfirmed)
    - No specific age criteria
  - First illness onset in 1996 or later
  - Animal exposure categories including backyard poultry (chickens, chicks, ducks, ducklings) reptiles, amphibians, small mammals (hamsters, guinea pigs, rats, mice, ferrets, rabbits, gerbils, hedgehogs)
  - Zoonotic pathogens of interest (*Salmonella*, avian influenza virus, LCMV, *Streptobacillus moniliformis*, Seoul virus, and others)
- **Exclusion criteria:**
  - International outbreak
  - Outbreak among animals with no human cases
  - Human cases exposed internationally
  - Outbreaks caused by animal species outside the scope of this Compendium
  - Foodborne disease outbreaks
  - Outbreak that occurred prior to 1996
  - No outbreak or case report information provided

### NORS/ACOSS Data Request

- Reports were included if they met the above criteria and were not already reported in published literature
- If discrepant case data between NORS/ACOSS report and published article, case numbers from NORS/ACOSS were used because they are updated in real time as reported by the jurisdictions
- Limitations of NORS/ACOSS data include that NORS reporting is voluntary (and health agencies may have limited ability to investigate/report these outbreaks), and NORS is a dynamic system, meaning that agencies can add/modify/remove reports as info becomes available. NORS only collects outbreak data, thus the true burden of enteric disease associated with animal contact is unknown (National Outbreak Reporting System (NORS), 2019).
- Limitations of ACOSS data include that there are not etiology confirmation guidelines for animal contact outbreaks as there are for foodborne outbreaks, and ACOSS did not incorporate a variable for confirmed/suspected animal sources until 2017 (Animal Contact Outbreak Surveillance System, 2020).

### Appendix B. Table of Selected Zoonoses

Select zoonotic diseases of small mammals, backyard poultry, reptiles, amphibians, fish, and selected other animals

**Table:**

Disease	Causative agent	Species associated with transmission to humans	Transmission route to humans	Symptoms in humans and clinical signs in animals
*Aeromonas* infection	Bacteria (*Aeromonas* species)	Fish and amphibians	Infection through open wounds and drinking contaminated water	Infection most commonly occurs in immunocompromised individuals and causes diarrhea or blood infections. Amphibians and fish may exhibit limb and fin discoloration and internal bleeding (Reptiles and Amphibians, 2022)
Avian mites	Parasitic mite (*Dermanyssus gallinae*, *Ornithonyssus sylvarium*)	Pigeons or bird nests near the home, pet birds, gerbils (Lucky et al, 2001), live poultry	*D. gallinae* is found in the bird's environment, including nesting materials. *O. sylvarium* spends its life on the host and is transmitted by close contact with infected birds (Boseret et al, 2013).	People most often experience pruritic skin lesions. Birds may exhibit depression, potentially life-threatening anemia, and increased chick mortality (Boseret et al, 2013).
Baylisascaris	Parasitic roundworm (most commonly *Baylisascaris procyonis*)	Raccoons are the definitive host and may spread this parasite to other species, including poultry. Infection has also been observed in a pet kinkajou (Taira et al, 2013). Dogs, though not an NTP, can be infected as an aberrant host and can shed eggs in their feces.	Ingestion of roundworm eggs found in raccoon feces, contaminated food, or objects(Wildlife, 2021)	People may experience severe disease from parasitic migration into the eye (ocular larval migrans), organs (visceral larval migrans), or brain (neural larval migrans) (Wildlife, 2021).
Campylobacteriosis	Bacteria (*Campylobacter*)	Poultry, rodents (including hamsters, guinea pigs, and gerbils) (Small Mammals, 2019). The bacteria have been detected in lizard and chinchilla feces (Turowski et al, 2014; Whiley et al, 2016).	Ingestion of contaminated food, water, or contact with feces from infected animals (Small Mammals, 2019).	People may have diarrhea, cramping, abdominal pain, and fever within 2–5 days after exposure. Many animals, including rodents, can carry the bacteria without any signs of illness; however, some may have diarrhea.
Cheyletiellosis	Parasitic mite (*Cheyletiella* species)	Rabbits, ferrets(Ferrets, 2022).	Direct contact with an infected animal	*Chyletiella* can temporarily infest people, causing skin irritation and itching. Infested animals are often subclinical, but may have hair loss, dandruff, and itching (Small Mammals, 2019).
Chlamydophila	Bacteria (*Chlamydophila psittaci*)	Psittacine birds, poultry (Henrion et al, 2002; Moroney et al, 1998; Thomas et al, 2017; Vanrompay et al, 2007)	Inhalation of aerosolized bird feces or respiratory tract secretions, either from direct contact with infected birds, or indirect environmental exposure (Thomas et al, 2017)	Illness severity in people may range from more common, self-limiting influenza-like-illness to less common fulminant psittacosis. Rarely, maternal or fetal illness and death may occur (Thomas et al, 2017). Birds may have a subclinical infection or have signs ranging from mild upper respiratory disease or nonspecific signs, including diarrhea, and signs of liver disease such as excretion of green to yellow-green urates. In severe cases, death may occur (Thomas et al, 2017).
Cryptococcosis	Fungus (*Cryptococcus neoformans*)	Pigeons most commonly, but may also be present in pet psittacine and passerine birds (Lugarini et al, 2008).	Inhalation of dust and soil containing dried contaminated bird droppings	Symptoms in people can resemble pneumonia, including cough, shortness of breath, and fever. Cryptococcal meningitis can cause headache, fever, and neck pain. Birds' symptoms are usually subclinical (“Birds Kept as Pets,” 2019).
Dermatophytosis (ringworm)	Fungus (commonly *Trichophyton* or *Microsporum* species)	Guinea pigs (Day, 2016; Kraemer et al, 2013), hedgehogs, and rabbits (Cafarchia et al, 2012; Day, 2016; Donnelly et al, 2000; Kraemer et al, 2013; Small Mammals, 2019)	Direct contact with an infected animal's skin or hair, or contaminated fomites.	Humans can have itchy, red, circular lesions with hair loss. Animals may be sub-clinically infected, or have circular areas of pruritic, reddened, skin and hair loss (Small Mammals, 2019).
Eastern equine encephalitis (EEE)	Arbovirus	Reptiles and amphibians,(Graham et al, 2012) birds, emu, horses, and other mammals (Corrin et al, 2021; Veazey et al, 1994)	Mosquito bite	EEE is a rare cause of encephalitis; approximately 30% of people with EEE die. In emus, marked depression, hemorrhagic diarrhea, and emesis.
Envenomation	Venom (secretion containing toxic proteins, enzymes, or other compounds)	Venomous species of snakes, fish, spiders, snails, and centipedes	Bite or sting from a venomous species	The type of venom determines the effect on people, varying from hematologic (coagulative or anemic) to neurologic (often paralytic) signs. The envenomating animal is not affected (Warwick and Steedman, 2012)
Erysipelas	Bacteria (*Erysipelothrix rhusiopathiae*)	Fish, poultry, swine, emus (Eriksson et al, 2009)	Infection usually occurs through open wounds, especially on the hands	Three different syndromes in people are possible: erysipeloid, a generalized cutaneous form, and a septicemic form with endocarditis. Clinical signs in animals varies by species. In birds, it most commonly causes sudden death, swollen hocks, and cutaneous lesions. Healthy fish can carry this bacterium on their scales without any signs of illness (Eriksson et al, 2009).
Giardiasis	Protozoal parasite (*Giardia)*	Relatively uncommon in rodents but has been associated with chinchillas, rats, and mice, and other small mammals such as rabbits (Pantchev et al, 2014)	Ingestion of food or water contaminated with infected feces.	Both people and animals experience diarrhea, greasy stools, and dehydration. People may experience abdominal cramps, nausea, and vomiting lasting up to two weeks (Small Mammals, 2019; Pantchev et al, 2014)
Leptospirosis	Bacteria (*Leptospira*)	Rodents including rats, mice, and other domestic species including cattle, pigs, horses, and dogs (Day, 2016; Friedmann et al, 1973; Himsworth et al, 2013; Leptospirosis, 2019)	Spread through the urine and other bodily fluids of infected animals.	People may be asymptomatic or have non-specific signs including high fever, headache, chills, muscle aches, vomiting, jaundice, abdominal pain, diarrhea, and rash. Disease may occur in two phases, where an initial disease phase is followed by a recovery period, and then a second more severe phase of disease (Day, 2016; Gaudie et al, 2008). Animals may carry and spread the bacteria without any clinical signs (Leptospirosis, 2019).
Melioidosis (aka Whitmore's disease)	Bacteria (*Burkholderia pseudomallei*)	Tropical freshwater fish have recently been associated with human infection (Melioidosis, 2021; Dawson et al, 2021). Sheep, goats, swine, horses, cats, dogs, and cattle are susceptible to infection.	Acquired by inhalation of contaminated dust or water droplets, ingestion of contaminated water, and ingestion of soil-contaminated food or contact with contaminated soil, especially skin abrasions. Recently, tropical freshwater fish have also been identified as a source of infection.	People may have localized infection (pain, swelling, fever, ulceration abscess), pulmonary infection (cough, chest pain, high fever, headache, anorexia), bloodstream infection (fever, headache, respiratory distress, abdominal discomfort, joint pain, disorientation), or disseminated infection (fever, weight loss, stomach or chest pain, muscle or joint pain, headache, central nervous system/brain infection, seizures). In animals, clinical signs vary depending on the site of infection and can range from acute to chronic. Fever, anorexia, swollen glands, or caseous nodules/abscesses. Subclinical infection is common (Low Choy, 2016).
Mycobacteriosis	Bacteria (*Mycobacterium marinum*)	Fish, amphibians, reptiles, hedgehogs (Bouricha et al, 2014; Hashish et al, 2018; Disease Information—Mycobacteriosis, 2020; Riley and Chomel, 2005)	Spread through contaminated water or contact with infected animals	People may experience granulomatous or nodular skin and tissue lesions that may progress to involve tissues. Disseminated infection can occur in immunocompromised patients (Akram and Aboobacker, 2021). Fish and reptiles may be asymptomatic or may be affected by open sores, granulomatous lesions, or deformed bones (Reptiles and Amphibians, 2022; Bouricha et al, 2014).
Newcastle disease	Virus (Newcastle disease virus)	Live poultry, pigeons, parrots, and other birds	Direct contact with large quantities of virus from infected animals, or contaminated materials in their environment including feces	People typically experience mild illness, with conjunctivitis being the most common symptom. Severity and progression of disease in birds depends on the viral strain and species of bird. Some birds may be sub-clinically infected. Infection with mild strains may cause respiratory signs including coughing, gasping, and sneezing. Infection with more severe strains, especially in chickens, may additionally cause decreased egg production, swollen head and neck tissues, watery green or white diarrhea, neurologic signs, and even sudden death (“Virulent Newcastle Disease,” 2021; “Disease Information—Newcastle Disease,” n.d.).
Pasteurellosis	Bacteria (*Pasteurella multocida*)	Rabbits and rodents including hamsters, guinea pigs, rabbits, rats, and mice (Small Mammals, 2019)	Often transmitted from small mammals through animal bites and scratches	Illness in people from infection with *Pasturella* is uncommon but symptoms may include painful wounds and skin infections from animal bites. Most small mammals do not show signs of illness. Some rabbits develop a respiratory disease (“snuffles”), including nasal and ocular discharge. The lungs, skin, and reproductive tracts of rabbits may also be affected (Small Mammals, 2019).
Visceral pentasomiasis	Parasitic lung worm (many genera)	Snakes, crocodilians	People whose diet includes snake meat, workers at Asian snake-farms, snake keepers in zoos and pet shops, veterinarians, and snake owners may be exposed to ova present in snake secretions or meat.	Rare in the United States but may be observed in immigrants from endemic areas and long-term travelers. Symptoms in people vary depending on the organ systems involved. Abdominal pain, cough, septicemia, and death are possible, though most infections are asymptomatic (Tappe and Büttner, 2009). In reptiles, these organisms live in the lungs and cause either subclinical infection or cause secondary bacterial or fungal pneumonia (Paré, 2008).
Plague	Bacteria (*Yersinia pestis*)	Rabbits, cats, and rodent species including squirrels, wood rats, ground squirrels, prairie dogs, chipmunks, mice, and voles (Campbell et al, 2019; “Ecology and Transmission,” 2019; Melman et al, 2018; von Reyn et al, 1976)	Infection occurs from bites from infected rodent fleas or from handling tissues and bodily fluids from plague-infected animals.	Both people and animals can have one of three principal forms of plague: bubonic, septicemic, or pneumonic. In people, bubonic plague is the most common form and begins with sudden onset of high fever, chills, headache, malaise, and myalgia, along with a bubo (a swollen and painful draining lymph node) most commonly in the femoral or inguinal lymph nodes. Plague causes variable disease in rodents, from subclinical infection or mild respiratory signs to severe, rapidly fatal disease.(“Ecology and Transmission,” 2019)
Rabies	Virus (rabies virus)	Affects all mammals, but only rarely occurs in small mammals such as squirrels, rats, mice, hamsters, gerbils, chipmunks, guinea pigs, ferrets, rabbits, and hares. (“Other Wild Animals,” 2021; Schlossberg, 2016.)	Infection occurs through bites, scratches, or other types of exposure of broken skin of mucous membranes to infectious materials from rabid animals.	Human signs occur days to months after exposure and include generalized weakness, fever, headache, confusion, behavioral changes, and delirium (“What are the signs and symptoms of rabies,” 2021; Schlossberg, 2016). Infected animals may present with a variety of signs but most often show early nonspecific signs, acute neurologic signs, and death (Edison et al, 2005; Small Mammals, 2019)
Rat lungworm infection	Parasitic worm (*Angiostrongylus cantonensis*)	Rats are the definitive host	Larvae are passed in rat feces, which develop in mollusk intermediate hosts to become infectious. Humans are infected by consuming the intermediate host or by consuming an amphibian or crustacean that ate an infected mollusk.	People may or may not have symptoms. In those who do, symptoms can include headache, stiff neck, tingling or painful feelings in the skin, low-grade fever, nausea, and vomiting. Rat hosts may lose weight (Jarvi et al, 2017).
Salmonellosis	Bacteria (*Salmonella* species)	Rodents and other small mammals, reptiles, amphibians and other aquatic species, feeder animals (frozen and live), backyard poultry, and other animals (Basler et al, 2016; Behravesh et al, 2014; Bosch et al, 2016; Cartwright et al, 2016; Fuller et al, 2008; Gaffga et al, 2012; Hale et al, 2012; Kiebler et al, 2020; Walters et al, 2016).	Ingestion after contact with infected fecal material, contaminated cages, coops, and bedding, or contaminated fur or feathers or eggs	Most people recover without treatment after 4–7 days of illness including diarrhea, fever, and abdominal cramps. Patients with severe illness may require hospitalization. Rarely, infection may spread from the intestines into the bloodstream, causing systemic illness and death. Animals may be asymptomatic carriers or have symptoms similar to humans (Salmonella Infection, 2015).
Sarcoptic mange	Parasitic mite (*Trixacarus caviae*)	Has been rarely associated with guinea pigs(Eshar and Bdolah-Abram, 2012; Honda et al, 2011; Nath, 2016)	Direct contact	People may be transiently affected with a pruritic local skin reaction. Animals may be sub-clinically infected or exhibit pruritis, erythema, and hair loss (Honda et al, 2011).
Tularemia	Bacteria (*Francisella tularensis*)	Rabbits, hares, rodents (including muskrats, prairie dogs, hamsters, and others), domestic cats and other species (“Tularemia,” 2018; Feldman, 2005; Stidham et al, 2018)	Tick bites, deer fly bites, skin contact with infected animals or infected tissues, ingestion of contaminated water, inhalation of contaminated aerosols or agricultural dusts, and lab exposure (“Tularemia,” 2018)	People may experience symptoms that range from mild to life-threatening and are dependent upon on how the bacteria enter the body, and typically include fever. The most common form of disease is ulceroglandular, manifesting as a skin ulcer at the site of infection, chills, head and muscle pain, prostration, and potential progression to septicemia. Other forms of disease include glandular, oculoglandular, oropharyngeal, pneumonic, and typhoidal. Infected rabbits and rodents are usually found dead, but may exhibit weakness, fever, lymphadenopathy, and abscesses (“Tularemia,” 2018; Feldman, 2005)
Zoonotic influenza	Virus (influenza type A virus)	Poultry, swine, and other animals (“Influenza in Animals,” 2018)	Direct contact with infected animals; indirect contact with virus contaminated surfaces; inhalation of aerosolized virus	People infected with zoonotic animal influenza viruses may have mild to severe symptoms that include fever, cough, sore throat, muscle aches, difficulty breathing, pneumonia, and even death.(“Avian Influenza A Virus Infections,” 2022) Ferrets are very susceptible to infection with influenza viruses, including human seasonal influenza A viruses and avian and swine influenza A viruses. Ferrets can have a variety of symptoms including fever, nasal discharge, sneezing, coughing, decreased appetite, and weakness (Ferrets, 2022) Poultry may have mild to severe illness ranging from decreased egg production to death, depending on the subtype and pathogenicity of the avian influenza virus. (“Influenza in Animals,” 2018) Other animals such as cats and dogs may have mild to severe signs (“Influenza in Animals,” 2018; “Influenza in Cats,” 2018)

### Appendix C. Glossary

**Breeder:** For the purposes of this Compendium, a breeder is any operation, regardless of size, that maintains a population of animals for the purpose of commodification (e.g., sale, trade, or swap) of the animals or their offspring. Some breeders are required to be licensed by USDA or state agencies. Most large breeders sell animals through a supply chain that may include a distributor and where a retailer is the final point of sale; however, some small breeders may sell or trade animals via other venues, such as flea markets, swap meets, and/or social media groups. Breeders are increasingly also selling directly to owners via the internet. They also may trade or sell animals to other breeders. Many large breeders are also distributors and/or importers.

**Commercial shipping:** Shipping of animals through air and ground carriers (e.g., FedEx, UPS, and the US Postal Service).

**Commercial transport:** Transport of animals in vehicles dedicated to this purpose, such as animal delivery vans and/or trucks.

**Distributor/Importer:** For the purposes of this Compendium, a distributor is any operation, regardless of size, that purchases animals from breeders, other domestic distributors, or international sources and houses them on a short-term basis for distribution to retailers and direct sales to owners. Distributors sometimes “cross-dock” animals, meaning they receive animals from a source and immediately distribute them to their next destination without removing them from their shipping containers. Some distributors accept returns of animals from retailers for various reasons (e.g., illness, size, disposition). Animal brokers conduct similar business as animal distributors, including selling and reselling or negotiating the purchase of animals, but they may or may not take possession or control of the animal and therefore may be subject to different regulations (“Licensing and Registration Under the Animal Welfare Act,” 2019).

**Drop-shipping:** Occurs when a hatchery is unable to fill a customer's order from its own supply, so they obtain birds from other hatcheries to fill the order. Drop-shipping can lead to mixing birds from multiple hatcheries in one shipment, thus retail stores and consumers will not know from which hatchery their birds originated and if that hatchery was part of USDA's National Poultry Improvement Plan program (Nichols et al, 2018).

**International importation:** Bringing animals originating in foreign countries into the United States for subsequent sale.

**Multiplying:** Occurs when a hatchery obtains fertile eggs from other hatcheries and incorporates the poultry resulting from those eggs into its breeding stock. Multiplying can introduce *Salmonella* from other flocks into the hatchery (Nichols et al, 2018).

**Multistate outbreak:** For the purposes of this Compendium, a multistate outbreak included in the literature review and NORS data request is defined as an outbreak in which the exposures occurred in more than one state (Appendix B. Reporting Multistate Exposure and Residency Outbreaks, 2017).

**Non-commercial transport:** Transport of animals in vehicles not dedicated to this purpose, such as privately owned vehicles and/or passenger airplanes.

**Owners:** For the purposes of this Compendium, an owner is a person who purchases or otherwise acquires animals for the purpose of keeping them as pets (rather than for breeding, as defined above). Most owners purchase animals directly from retailers, though internet sales are becoming increasingly important. Less commonly, owners acquire animals at swap meets, flea markets, or other venues. Internet sales and non-retailer venues are not subject to federal regulations.

**Retailer:** For the purposes of this Compendium, a retailer is a commercial entity that purchases animals from breeders, distributors, and/or importers for subsequent sale at a physical (“brick-and-mortar”) storefront or via the internet. Physical retail stores are the only points of sale that are subject to federal regulations. Some retailers are increasingly also selling directly to owners via the internet or operate exclusively through online sales. Online sales are not subject to federal regulations. Retailers are also considered pet and agricultural industry employers who are responsible for implementing occupational health recommendations found in this Compendium.

**Roadside stand:** For the purposes of this Compendium, a type of marketing site in which an animal is sold directly to consumers that may be a temporary or semi-temporary structure.

**Transporter:** A person with a commercial business that moves animals from one location to another is considered a transporter under the Animal Welfare Act and must be registered with USDA unless they qualify for an exemption. Examples include airlines and trucking companies (“Licensing and Registration Under the Animal Welfare Act,” 2019).

**Trans-shipping:** Occurs when hatchlings or fertile eggs from commercial hatcheries, mail-order hatcheries, or small-scale producers come through the mail-order hatchery, allowing poultry from multiple hatcheries to be mixed before distribution to customers (Nichols et al, 2018). Trans-shipping practices can introduce *Salmonella* from other flocks into the hatchery.

### Appendix D. Industry Layout

This diagram outlines the common distribution pathways, via sales or trade, of non-traditional pets in the United States pet trade. These movement pathways are important to understand traceback and trace-forward of animal shipments in the event of a disease outbreak.

NTPs may be imported from breeders internationally or obtained from domestic breeders in the United States. The solid lines illustrate the most common distribution pathway whereby a high volume of animals is moved, usually through sales, from breeders via importers or distributors to retailers and then to pet owners. The dashed lines illustrate less common pathways, representing a lower volume of animal movement for either sales or trade (including internet sales) or returns. The dashed lines show that less frequently, animals are sold or traded from the distributor, importer, or domestic breeder directly to the pet owner instead of being sold through a retailer. The dashed lines also indicate a lower volume of animal movement to account for returned animals, whereby an animal may move from the pet owner back to the breeder or retailer; from the retailer, importer, or distributor back to the breeder; or from the retailer back to the distributor.

Generally, NTP breeders, distributors, or importers have written contracts only with the major pet retailers. These contracts represent the largest numbers of animals and highest volume of animal movement (depicted here by the solid lines). The contracts include specifics describing animal costs, shipping, return policy, size, etc. Animals are categorized using visual standards where grade A are considered “perfect” animals with no known abnormalities, followed by grade B and grade C animals. Major pet retailers only take grade A animals. Other animals including grade A not sold, grade B, and C are sold to the smaller retail companies, independent retailers and through internet sales. More information is found in Appendix G. Selected recommendations, standards, guidelines, and recommendations for non-traditional pet species, including information on bringing turtles into the United States (Turtles | Bringing an Animal into U.S., 2022).

Mechanisms of movement include both commercial and non-commercial ground transport as well as commercial air shipping. Most small mammals are delivered to retail stores via non-commercial vehicles owned by the breeders, brokers, distributors, or importers. These non-commercial vehicles service multiple retail stores along a specified route in a region of the country on a weekly basis. Animals are delivered to any retailer on their route that has placed an order including major retailers, smaller retailers, and independent retailers. In general, most animals are packaged for specific stores; however, non-commercial vehicles carry extra animals in case they are needed. Upon the animals' arrival, retail store employees have a specified amount of time to examine and either accept or reject the animals. Rejected animals go back on the truck and continue the route. When vehicles finish their routes, they return the animals that have been rejected or simply not sold to the breeders, distributors, or importers. Larger breeders, distributors, and importers may have multiple species of animals (aquatic, reptile, avian) along with mammals that are all delivered on company vehicles to retail stores.

Other NTP species such as reptiles, amphibians, and backyard poultry may be sold directly from large or small breeders to retail stores and are generally delivered via commercial carriers that will transport live animals.

Some common reasons that animals may be returned to breeders, distributors, or importers from retailers include dermatophytosis (ringworm) in small mammals (most commonly affecting guinea pigs and rats), missing toes, incorrect size (e.g., too small or too large), too young, or incorrect fulfilment (e.g., shipped the incorrect animal). Some breeders accept returns of animals from distributors and retailers; however, it is unusual for a breeder to accept returns directly from an owner. Some breeders will not accept animal returns because it is a biosecurity risk for their population, and once an animal leaves the premises, per biosecurity principles, they should not return. It is common for retailers to accept returns directly from owners, depending on store policy.

### Correct handwashing procedure

- Wet hands with clean, running water (warm or cold work equally well) and apply soap.
- Rub hands together to make a lather and scrub them well (be sure to scrub the backs of hands, between fingers, and under nails); continue rubbing hands for at least 20 seconds (the length of time it takes to sing “Happy Birthday” twice).
- Rinse hands well under running water.
- Dry hands with a clean, disposable paper towel or air dry them.
- Assist young children with washing and drying their hands.

Tools to teach and encourage children to wash their hands can be used in schools, homes, or venues catering to children. (https://www.cdc.gov/handwashing/training-education.html)

### Recommendations regarding hand sanitizers

If soap and water are not available, using an alcohol-based hand sanitizer that is at least 60% alcohol can quickly reduce the number of pathogens (germs) on hands, but it will not eliminate all types of germs. Hand sanitizer is not as effective when hands are visibly dirty or greasy. To improve effectiveness, visible contamination and dirt should be removed before using hand sanitizers.

Even when hand sanitizer is used, hands should always be washed as soon as possible after handling NTPs.

### Correct use of hand sanitizers

- Apply product to the palm of one hand.
- Rub hands together.
- Rub the product over all surfaces of hands and fingers until your hands are dry.

### Establishment and maintenance of handwashing facilities or stations in areas where animals are housed, displayed, or treated (Daly et al, 2017)

- The number of handwashing facilities or stations should be sufficient for the maximum anticipated in attendance, and facilities should be accessible for children (i.e., low enough for children to reach or equipped with a stool) and people with disabilities as well as the general population
- Handwashing facilities and stations should be conveniently located in transition areas between animal and non-animal areas and in employee break rooms, kitchens, and other areas where food is prepared, stored, served, or consumed
- Maintenance of handwashing facilities and stations should include routine cleaning and restocking to ensure an adequate supply of paper towels and soap
- Running water should be of sufficient volume and pressure to remove soil from hands. Volume and pressure might be substantially reduced if the water supply is furnished from a holding tank; therefore, a permanent, pressurized water supply is preferable
- Handwashing stations should be designed so that both hands are free for handwashing by having operation with a foot pedal or water that stays on after hand faucets are turned on
- Liquid soap dispensed by a hand pump or foot pump is recommended
- To encourage handwashing, set the water at a comfortable temperature
- Communal basins, in which water is used by more than 1 person at a time, are not adequate handwashing facilities.
- Handwashing stations should not be used to wash dishes, containers, bedding, or other material in contact with animals or their environment
- Handwashing policies should be included in employee safety guidelines and trainings

In places where human-animal contact occurs, signs regarding proper handwashing practices are critical to reduce disease transmission.

### **Handwashing sign recommendations** (Daly et al, 2017)

- Signs that remind employees and visitors to wash hands should be posted at exits from animal areas (e.g., exit transition areas) and in areas where food is served and consumed
- Signs should be posted that direct all employees and visitors to handwashing stations when exiting animal areas
- Signs with proper handwashing instructions should be posted at handwashing stations and in restrooms to encourage proper practices
- Handwashing signs should be available in multiple age-appropriate and language-appropriate formats

### Appendix F. Guidelines for Animals in Schools, Childcare Settings, and Long-Term Care and Assisted Living Facilities

Schools, childcare settings (daycares or other), and long-term care and assisted living facilities include a higher proportion of people in high-risk groups for serious illness in the case of a zoonotic disease (children younger than 5 years old, and adults 65 and older) than the general population. Some animals should not visit or be housed in these or other locations where high-risk groups congregate.

Parents or guardians should be consulted prior to the introduction of animals in the classroom or on field trips for children of any age. There may be special considerations needed for children who have health conditions that might increase their susceptibility to injury or illness, such as animal allergies, immunocompromising conditions, or special behavioral needs. Some of these considerations might not be known by or apparent to teachers or caregivers. No animal contact can be rendered completely safe, and good judgment must be used to select animals of appropriate species and temperament, in consultation with parents.

Similar considerations should be applied when introducing animals to long-term care and assisted living facilities as residents may have underlying health conditions that compromise their immune system.

**Some animal species are not appropriate for introduction into school or childcare settings, or long-term care and assisted living facilities. These include but are not limited to the following:**

- Animals that pose a high risk for zoonotic disease transmission (e.g., preweaned calves, reptiles, amphibians, rodents, and some other small mammals [e.g., hedgehogs], and backyard poultry) or bites (e.g., ferrets)
- Inherently dangerous animals (e.g., lions, tigers, cougars, and bears)
- Nonhuman primates (e.g., monkeys and apes)
- Mammals that pose a high risk for transmitting rabies (e.g., bats, raccoons, skunks, foxes, mongoose, and coyotes)
- Aggressive or unpredictable wild or domestic animals
- Stray animals with unknown health and vaccination history
- Venomous or toxin-producing animals or insects

### Handwashing

When animals are being used for educational purposes in classrooms with children of any age, or as educational or emotional support purposes in long-term care and assisted living facilities, ensure that appropriate handwashing (Appendix E. Handwashing) is implemented.

### General recommendations

- Animals are effective and valuable teaching aids, and provide comfort and emotional support, but safeguards are required to reduce the risk for infection and injury
- Ensure that teachers and staff know which animal species are inappropriate as residents or visitors to the facility and which animals should not be in direct contact with children or residents of long-term care and assisted living facilities
- Ensure that personnel providing animals for educational purposes are knowledgeable regarding animal handling and zoonotic disease issues.
- People or facilities that exhibit regulated animals to the public should be licensed by the USDA.
- Inform parents of the presence of animals as well as the benefits and potential risks associated with animals in school classrooms. Consult with parents to determine special considerations needed for children who are immunocompromised, have allergies or asthma, or have special behavioral needs.
- Educate children about harmful germs that can spread between animals and people and about proper handwashing technique
- Wash hands right after contact with animals, animal products, or feed, or after being around animal environments
- Supervise human-animal contact, particularly involving children younger than 5 years old
- Display animals in enclosed cages or under appropriate restraints
- Do not allow animals used in schools, daycares, or long-term care or assisted living facilities to roam, fly free, or have contact with wild animals
- Designate specific areas for animal contact. Do not allow food or drink in animal contact areas; do not allow animals in areas where food and drink are stored, prepared, served, or consumed
- Clean and disinfect all areas where animals and animal products have been present. Children should perform this task only under adult supervision.
- Do not clean animal cages or enclosures in sinks or other areas used to store, prepare, serve, or consume food and drinks
- Obtain a certificate of veterinary medical inspection, proof of rabies vaccination, or both according to local and state requirements for the species being exhibited
- Ensure veterinary medical care, including preventive health programs for endoparasites and ectoparasites as appropriate for the species

### Animal-specific recommendations when selecting an NTP

Please see the Animal Contact Compendium and general guidelines for information on species not listed here.

- **Rodents** (guinea pigs, rats, mice, prairie dogs, hamsters, gerbils) **and some other small mammals** (e.g., hedgehogs) (Small Mammals, 2019)
-    Pet rodents and some other small mammal species are not recommended for households with children younger than 5 years old, adults 65 and older, people with weakened immune systems, or pregnant people because these groups are at higher risk for serious illness.
-    Do not keep pet rodents and other small mammals in childcare centers, nursery schools, or other facilities with children younger than 5 years old or facilities that care for adults 65 and older, and those with weakened immune systems (e.g., long-term care facilities and nursing homes).
- **Ferrets**
-    Ferrets (and other mustelids such as mink) are not recommended as pets in households with children younger than 5 years old.
-    Young children should never be left unsupervised with a ferret, and ferrets should not be allowed to roam freely because of the risk for bites, scratches, and unprovoked attacks.
-    Rabies vaccination is recommended for pet ferrets and is required by some states (Compendium of Animal Rabies Prevention and Control, 2016; State Rabies Vaccination Laws, 2021).
- **Reptiles** (turtles, lizards, snakes)
-    Reptiles are not recommended for households with children younger than 5 years old, adults 65 and older, people with weakened immune systems, or pregnant people because these groups are at higher risk for serious illness.
-    Children younger than 5 years old, adults 65 and older, and people with weakened immune systems should not handle or touch reptiles, or anything in the area where they live and roam.
-    Do not keep reptiles in childcare centers, nursery schools, primary schools, or other facilities with children younger than 5 years old or facilities that care for older adults and those with weakened immune systems (e.g., long-term care facilities and nursing homes).
- **Amphibians** (African dwarf frogs, salamanders)**, fish, and other aquatic animals**
-    Amphibians are not recommended for households with children younger than 5 years old, adults 65 and older, people with weakened immune systems, or pregnant people because these groups are at higher risk for serious illness.
-    Do not let children younger than 5 years old touch aquariums or aquarium water or feed fish and other aquatic animals. Children younger than 5 years old are more likely to get sick from exposure to germs like *Salmonella* that may be present in aquatic animal habitats.
-    Do not keep amphibians in childcare centers, nursery schools, primary schools, or other facilities with children younger than 5 years old or facilities that care for older adults and those with weakened immune systems (e.g., long-term care facilities and nursing homes).
- **Backyard poultry** (chicks, chickens, ducks, ducklings)
-    Backyard poultry are not recommended for households with children younger than 5 years old, adults 65 and older, people with weakened immune systems or pregnant people because these groups are at higher risk for serious illness.
-    Do not let children younger than 5 years old handle or touch chicks, ducklings, or other live poultry. Children younger than 5 years old are more likely to get sick from exposure to germs like *Salmonella*.
-    Do not keep backyard poultry in childcare centers, nursery schools, primary schools, or other facilities with children younger than 5 years old or facilities that care for older adults and those with weakened immune systems (e.g., long-term care facilities and nursing homes).
- **Feeder animals** (feeder rodents and chicks)
-    Children younger than 5 years old, adults 65 and older, people with weakened immune systems, and pregnant people should not touch or handle frozen or live feeder rodents or other feeder animals.
- **Venomous or toxin-producing animals** should not be kept as pets in any setting (Warwick and Steedman, 2012).
- **Psittacine birds** (e.g., parrots, parakeets, and cockatiels) consult Birds Kept as Pets | Healthy Pets, Healthy People | CDC and the NASPHV—Psittacosis and Chlamydiosis (Thomas et al, 2017) and seek veterinary medical advice.

### Appendix G. Selected Recommendations, Standards, Guidelines, and Regulations for Non-Traditional Pet Species

Certain federal agencies and organizations in the United States have standards, recommendations, and guidelines for reducing health risks associated with contact with NTP species. The recommendations in this Compendium use those existing recommendations as well as information, publications, reports, and guidance documents from multiple organizations, and existing laws and regulations in the United States.

This information is current as of December 2021; however, information continues to be updated and a review of recent standards, recommendations, and guidelines should also occur when seeking specific information.

Some of these standards, recommendations, and guidelines include state and federal regulations such as the Animal Welfare Act (AWA), the only federal law in the United States that regulates the treatment of certain warm-blooded animals in research, exhibition, and transport, and by dealers. The AWA sets the minimum standard of care and treatment provided for certain warm-blooded animals bred for commercial sale, used in research, transported commercially, or exhibited to the public. At minimum, these standards include providing veterinary care, an appropriate diet, clean and structurally sound housing, proper ventilation and sanitation, and protection from extremes of weather and temperature. The AWA is enforced by the US Department of Agriculture (USDA), Animal and Plant Health Inspection Service, Animal Care Service which licenses and inspects certain animal facilities (“Licensing and Registration Under the Animal Welfare Act,” 2019). The USDA website provides more information on the AWA and which animals are covered under the Animal Welfare Act (usda.gov).

Public health and animal health partners at federal, state, and local levels have implemented surveillance systems and disease prevention programs to identify and prevent cases of *Salmonella* infectionsand outbreaks. *Salmonella* infection in people is a nationally notifiable disease, i.e., physicians and health laboratories are required to report cases to local health departments in accordance with procedures established by each state. However, the source of exposure in the case of an animal source is not routinely confirmed or collected in a standardized way. NORS and ACOSS data are voluntarily reported.

Another federal law, the Public Health Service Act, bans the sale of turtles with a shell length of less than 4 inches (10.16cm). The ban was initiated in response to increasing reports of *Salmonella* infection associated with contact with small turtles and is administered by FDA's Center for Veterinary Medicine (Salmonella and Turtle Safety, 2021). Additionally, some states have exercised their own authority to establish laws that limit the sale of small turtles, regulate how they are sold, establish state authority to test and destroy turtles or turtle eggs infected with salmonellosis, and limit the places where turtles may be kept (e.g., childcare facilities and medical facilities may not keep turtles in some states) (Menu of State Turtle-Associated Salmonellosis Laws, n.d.). CDC limits imports of turtles, terrapins, and their viable eggs; turtles with a shell length of less than 4 inches (10.16cm) and turtle eggs may not be imported to the United States for any commercial purpose (Turtles | Bringing an Animal into U.S., 2022).

Backyard poultry ownership is regulated at the local level in the context of an ordinance (Tobin et al, 2015). Local ordinances typically refer to which sex and how many poultry are allowed. To prevent *Salmonella* contamination of eggs, FDA issued “the egg rule” on July 9, 2009 which requires egg producers to implement measures to prevent *Salmonella* Enteritidis from contaminating eggs on the farm and from further growth during storage. However, the “egg rule” does not apply to the backyard poultry industry and thus hatcheries, including mail-order hatcheries, are not included under the “egg rule” (“21 CFR Parts 16 and 118 Prevention of Salmonella Enteritidis,” 2009). Since cases of *Salmonella* infection have increased in backyard poultry owners, public health has worked with hatcheries to implement enhanced biosecurity, education for poultry owners, and to develop *Salmonella* control programs. The National Poultry Improvement Plan (NPIP) is a voluntary state-federal cooperative testing and certification program administered by USDA for poultry breeding flocks, baby chicks, hatching eggs, hatcheries, and dealers. NPIP covers several poultry diseases, including programs to monitor *Salmonella* bacteria in poultry (National Poultry Improvement Plan (NPIP), 2020). USDA's Defend the Flock program provides biosecurity recommendations to prevent disease transmission for commercial and backyard poultry owners (Defend the Flock—Biosecurity 101, n.d.).

Following the 2003 monkeypox outbreak in the United States, CDC and FDA jointly banned importation of African rodents in 2003, but it is currently legal to sell captive-bred African rodents and prairie dogs in the United States. Wild-caught prairie dog pups are legal to sell as pets in many states. CDC's African Rodent Importation Ban provides more information on what rodents may or may not be imported to the United States and under what circumstances (“African Rodent Importation Ban,” 2015).

Several federal agencies oversee animal importation; more information is available here: Bringing Pets and Wildlife into the United States | U.S. Customs and Border Protection (cbp.gov)

In June 2021 FDA issued guidance to bring OTC medically important antibiotics for animals under veterinary oversight over a two-year period.

CDC has issued recommendations for preventing transmission of zoonotic pathogens from reptiles, amphibians, backyard poultry, and rodents and other small mammals to people (How to Stay Healthy Around Pets, 2021). In addition to this Compendium, NASPHV has developed guidance and compendia for human exposure to *C. psittaci*, rabies virus, *C. burnetii*, novel (including variant) zoonotic influenza A viruses, exposures to zoonotic pathogens in veterinary settings, and measures to reduce the risk of zoonotic disease transmission in public settings (Compendium of Animal Rabies Prevention and Control, 2016; Daly et al, 2017; Thomas et al, 2017).

### Appendix H. Selected Educational Resources

**Table:**

Association of Reptile and Amphibian Veterinarians (ARAV)	Resources for owners https://arav.org/for-owners/
American Veterinary Medical Association (AVMA)	**Species-specific considerations** to help choose a healthy pet are available on AVMA's website: https://www.avma.org/resources/pet-owners/petcare/selecting-pet-your-family
AVMA	Antimicrobial stewardship definition and core principles: ^*^ https://www.avma.org/policies/antimicrobial-stewardship-definition-and-core-principles
CDC	Detailed messages and educational materials: https://www.cdc.gov/healthypets/
CDC	Avian influenza: https://www.cdc.gov/flu/pdf/avianflu/avian-flu-transmission.pdf
University of Minnesota	Antimicrobial Resistance Learning Site: https://amrls.umn.edu/
University of Minnesota	Handbook of Antimicrobial Stewardship in Companion Animal Veterinary Settings https://arsi.umn.edu/handbook-antimicrobial-stewardship-companion-animal-veterinary-settings
Pet Advocacy Network	Healthy Herp Handling: https://pijac.org/HealthyHerpHandling
Pet Advocacy Network	Feeder rodent resources https://pijac.org/sites/default/files/pdfs/FeederRodentIndustryBMPSept2013.pdf
Pet Advocacy Network	Retailer resources https://pijac.org/animal-welfare-and-programs/zoonotic-disease-prevention/retailerresources/#stickers
USDA	Defend the Flock: backyard poultry resources https://www.aphis.usda.gov/aphis/ourfocus/animalhealth/animal-disease-information/avian/defend-the-flock-program/defend-the-flock-program

^*^ See Table 1. for additional resources on antimicrobial stewardship
